# Rainfall and Substrate Origin Driven Change of Ruminant Manure Microbiomes in Cyprus

**DOI:** 10.1111/1758-2229.70429

**Published:** 2026-10-06

**Authors:** P. Pissaridou, K. Philippou, A. Phokas, J. Rodosthenous, C. Hadjimichael, M. Omirou

**Affiliations:** ^1^ Department of Agrobiotechnology Agricultural Research Institute Nicosia Cyprus

**Keywords:** environmental stress, manure management, manure storage, microbial community dynamics, ruminant manure

## Abstract

Microbial communities drive the stabilisation of organic matter during manure storage; however, the combined influence of substrate origin and sudden rainfall occurrences on these communities remains poorly understood. For the first time in Cyprus, we investigated the effects of manure type (dairy cow vs. goat) and a drying–rainfall simulation pulse on the community composition, diversity, and functional potential during manure storage, using 16S rRNA metabarcoding and shotgun sequencing. Under dry conditions, substrate composition set the baseline, with the more structurally heterogeneous goat manure supporting significantly higher diversity than dairy cow manure. Rainfall simulation then acted as a dominant environmental filter, sharply reducing diversity and driving compositional convergence across manure types. This shift coincided with a rapid rise of fast‐responding, stress‐tolerant taxa, primarily Bacillota, and an increase in functions linked to sporulation and rapid growth. Ordination and dispersion analyses further showed that substrate origin modulates stability: after rainfall simulation, cow‐manure communities exhibited greater compositional displacement and greater variability than those from goat manure. Overall, the efficacy and stability of manure storage systems emerge from the interplay between initial physicochemical template and dynamic moisture stress. Optimising large‐scale operations will require management approaches that consider substrate characteristics, microbial community structure, and moisture dynamics.

## Graphical abstract

Simulated rainfall triggered strong ecological filtering in ruminant manures, overriding substrate‐driven differences between dairy cow and goat manure. While the more fibrous goat manure supported higher initial microbial diversity, rewetting led to community convergence dominated by spore‐forming bacteria. Enrichment of sporulation (*spm*A, *spm*B, *ytf*J) and stress‐response (*mec*A, *zup*T, *reg*X3, *ppk*, *rpl*L) genes reflects adaptive strategies to fluctuating moisture. Moisture, therefore, plays a key role in determining microbial resilience during the early stages of composting.

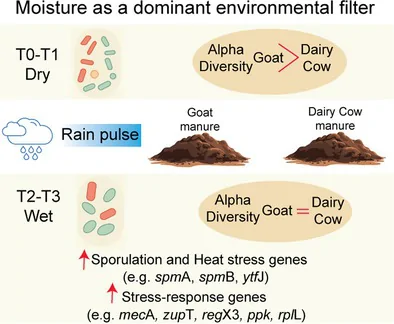

## 1 Introduction

Microbial communities play a central role in the decomposition of organic matter during composting, driving critical biogeochemical processes such as carbon mineralisation, nutrient transformation, and the stabilisation of organic waste (Insam and de Bertoldi 2007; Ryckeboer et al. 2003). The structure and function of these communities are influenced by various ecological filters, notably the substrate's chemical composition and prevailing environmental conditions. Among these, the type of manure and moisture availability are particularly influential; yet, their combined effects on microbial community dynamics during manure storage remain poorly characterised.

In Mediterranean countries such as Cyprus, summers are long and dry but punctuated by abrupt, sometimes intense rainfall events, which are now more frequent due to global warming (Zittis et al. 2021). When such events intrude on stored manure, they impose a rapid rainfall simulation pulse on a dry baseline. This pulse can reorganise pore connectivity, alter oxygen diffusion and redox potential, solubilise otherwise protected organic substrates, and mobilise nutrients and metals (Bastida et al. 2008). The net effect is a short, sharp shift in selective pressures that can favour copiotrophic or stress‐tolerant fast‐responding, moisture‐adapted microorganisms and alter the inferred functional potential of the microbial community (Evans and Wallenstein 2012; Placella et al. 2012). Although moisture has long been recognised as a master variable in composting, the specific scenario of unintended summer rainfall during storage, with its brief timing, spatial patchiness, and interaction with pre‐existing dryness, remains understudied.

Additionally, ruminant manures differ substantially in physical and chemical properties because of species‐specific digestive processes and variations in diet composition. For example, goat manure typically has a higher dry matter content and greater structural complexity due to higher intake of fibrous plant residues compared to dairy cow manure (Bernal et al. 2009; Gao et al. 2010). These differences may influence microbial colonisation patterns and niche diversity by altering the availability and heterogeneity of organic substrates. While previous studies have examined microbial succession in composts derived from different feedstocks (Chandna et al. 2013; Neher et al. 2013), few have directly compared how substrate origin modulates microbial ecology under controlled manure storage conditions.

Therefore, we investigated how manure type (dairy cow vs. goat) and rainfall simulation (dry vs. wet periods) interact to shape microbial community composition and diversity during manure storage under real farming conditions. Specifically, we asked: (1) How does the initial substrate influence alpha and beta diversity during manure storage? (2) What are the temporal patterns of microbial succession under distinct moisture conditions? and (3) How do substrate origin and moisture conditions interact to influence microbial succession patterns during rainfall simulation? We characterised microbial communities over time using 16S rRNA metabarcoding and Shotgun Sequencing, and applied diversity analyses and ordination to examine the relative contributions of substrate and moisture to community structure, dynamics, and affected metabolic processes. Our results provide new insights into how substrate to environment interactions influence microbial ecology during manure storage, with implications for the design and management of composting practices.

## 2 Materials and Methods

### 2.1 Site Location and Experimental Design

Measurements were carried out at the Agricultural Research Institute of Cyprus (35° 08′ 25″ N—33°23′02″ E, elevation 170 m) in Lefkosia during the summer of 2023 (July–August). Manure from lactating Holstein‐Friesian dairy cows and Damascus (Shami) goats was collected from the experimental barn of the Agricultural Research Institute (Mavrogenis et al. 2006). Dairy cows and goats were fed a diet of maize and barley grass, hay and concentrates (17% crude protein), with copper supplementation provided exclusively to the dairy cows.

### 2.2 Sample Collection and Processing

Manure samples were collected at four time points: Day 0 (T0), Day 14 immediately before rainfall simulation (T1), Day 18 following rainfall simulation (T2), and Day 27 at the end of the experiment (T3). At each time point, manure was sampled at 0–20 cm depth from four locations within each manure‐filled chamber and combined into one composite sample. Four independent chambers were used per manure type, providing four biological replicates per animal × timepoint combination. During rainfall simulation, 4 L of water was uniformly applied to each chamber, and post‐rainfall sampling was performed 3 days later to allow microbial responses to develop. Samples were homogenised, passed through a 2‐mm sieve and stored appropriately until analysis. T0–T1 were subsequently classified as the dry period and T2–T3 as the wet period.

Physicochemical characteristics and gaseous emissions from the same experimental platform were evaluated independently as part of a companion study focused on greenhouse gas and ammonia emissions under simulated rainfall conditions (Philippou et al., forthcoming). Detailed procedures are provided in the Supplementary Methods SM1.1.

### 2.3 Manure DNA Extraction and Quantification

Total DNA was extracted from homogenised manure using the DNeasy PowerSoil Pro Kit on the automated QIAcube platform (Qiagen), following the manufacturer's protocol. Three DNA extractions were performed per chamber. DNA concentration was quantified using the Quant‐iT PicoGreen dsDNA Assay Kit (Thermo Scientific), and DNA purity was assessed using a NanoDrop ND‐2000 spectrophotometer. Detailed DNA extraction procedures are provided in the Supplementary Methods SM1.2.

### 2.4 DNA Library Preparation and Next Generation Sequencing

Bacterial community composition was characterised by amplification of the V3–V4 region of the 16S rRNA gene followed by sequencing on an Illumina NextSeq 2000 platform. Libraries were prepared according to the Illumina 16S Metagenomic Sequencing Library Preparation protocol, with negative and mock‐community controls included during library preparation.

Shotgun metagenomic libraries were prepared from 200 ng of total environmental DNA using the Illumina DNA Prep (M) Tagmentation protocol. Libraries were quality‐controlled, quantified, pooled equimolarly and sequenced on the Illumina NextSeq 2000 platform. Detailed primer sequences, amplification conditions, library preparation procedures and sequencing parameters are provided in the Supplementary Methods SM1.3.

### 2.5 Bioinformatic Analysis

16S rRNA amplicon sequences were processed using the DADA2 pipeline (Callahan et al. 2016, Callahan et al. 2017), including quality filtering, denoising, paired‐read merging and chimera removal. Taxonomy was assigned against SILVA v138 using a bootstrap threshold of 75%. Alpha and beta diversity analyses were performed in R using microbiome, vegan and phyloseq, while differential abundance and related microbial community analyses were conducted using microeco and associated R packages.

Shotgun metagenomic sequences were processed using the standard MetaBakery workflow with default pipeline parameters. HUMAnN gene‐family abundances (CPM) were merged with UniProt annotations and curated to remove unmapped/unclassified features, entries lacking a UniProt primary accession, missing CPM values and zero‐variance features. Samples were grouped a priori into dry (T0–T1) and wet (T2–T3) periods for downstream analyses. Detailed bioinformatic processing, filtering and annotation procedures are provided in the Supplementary Methods SM1.4.

### 2.6 Statistical Analysis

Statistical analyses were conducted in R v4.3.2. Alpha diversity (Shannon, Observed and Simpson indices) was calculated from rarefied ASV data, with Shannon diversity used as the primary endpoint. Differences were assessed using linear mixed‐effects models followed by estimated marginal means and Tukey‐adjusted pairwise comparisons. Beta diversity was evaluated using Bray–Curtis dissimilarity and Principal Coordinates Analysis (PCoA), with group differences tested by PERMANOVA (999 permutations) and multivariate dispersion assessed using betadisper.

Differential ASV abundance between dry and wet periods was analysed separately for each manure type using DESeq2 following low‐prevalence filtering, with Benjamini–Hochberg correction for multiple testing. Indicator taxa were identified using permutation‐based multipattern analysis.

For shotgun metagenomic data, moisture‐associated differences in gene abundance were evaluated using feature‐level tests and two‐factor linear models incorporating moisture, animal type and their interaction. Estimated marginal means were used for post hoc contrasts, and all feature‐wise analyses were corrected using the Benjamini–Hochberg FDR method. Heatmaps and other visualisations were generated from CPM‐normalised gene abundances. Statistical significance was set at *p* < 0.05 unless otherwise specified. Full statistical procedures, filtering criteria, model specifications and visualisation methods are provided in the Supplementary Methods SM1.5.

## 3 Results

To guide changes in manure‐management practices, we first profiled the dominant microbial taxa (i.e., the key players) in dairy cow and goat manure using 16S rRNA gene amplicon sequencing. The manure microbiota of both dairy cows and goats were dominated by the phyla Bacillota, Actinomycetota, and Pseudomonadota (Figure 1A). In dairy cow manure, Bacillota ranged from 46.3% to 50.7%, followed by Actinomycetota (22.5%–31.1%) and Pseudomonadota (13.8%–22.4%). Bacteroidota was present at lower relative abundance (2.7%–6.3%), while minor contributions were observed for Chloroflexota (0.7%–0.8%) and Planctomycetota (up to 0.2%). In goat manure, Bacillota showed a higher relative abundance (52.3%–63.6%) than in cow manure, with Actinomycetota (18.2%–23%) and Pseudomonadota (8.5%–15.3%) following. Bacteroidota reached up to 9.9%, and the remaining phyla contributed less than 1%–2.5% of the community. Overall, this initial descriptive analysis reveals only minor differences in the microbial composition of the two manure types examined.

**FIGURE 1:**
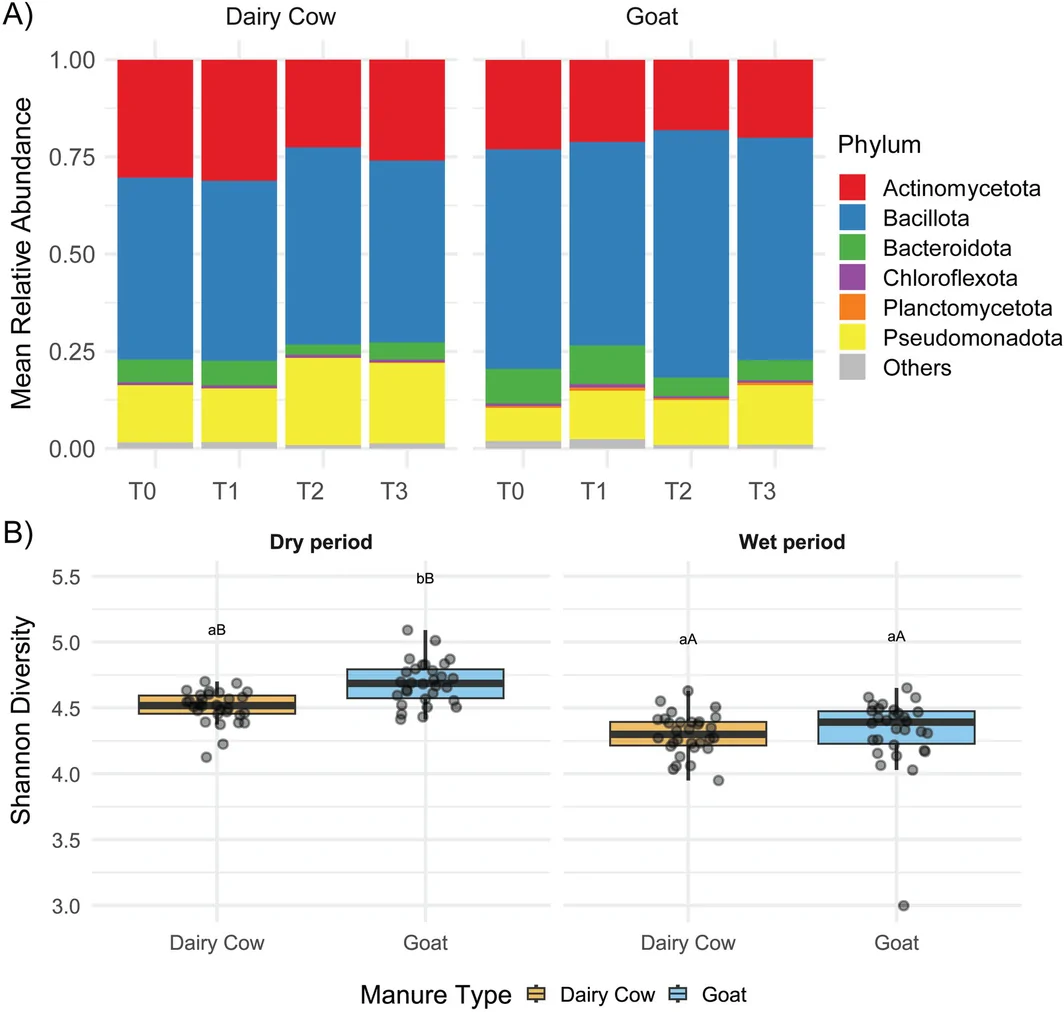
Microbial community composition and diversity responses to rainfall simulation in dairy cow and goat manure. (A) Mean relative abundance of dominant bacterial phyla across time points in dairy cow and goat manure. Bars represent the average community composition per manure type and time point. Only the six most abundant phyla are shown individually; all others are grouped as ‘Others’. Time points T0 and T1 represent the dry period, while T2 and T3 correspond to the wet period following water addition. (B) Shannon diversity of microbial communities in dairy cow and goat manure across Dry and Wet periods. Boxplots represent rarefied alpha diversity estimates for each manure type and moisture condition, with jittered points showing individual replicates. Letters indicate statistically significant differences based on post‐hoc comparisons from a linear mixed‐effects model: lowercase letters (e.g., a, b) denote differences between manure types within the same period, while uppercase letters (e.g., A, B) denote differences between periods within the same manure type (*p* < 0.05, Tukey‐adjusted).

To further investigate the microbiota composition of the two manure types, a Shannon diversity analysis was performed using a linear mixed‐effects model. The analysis revealed significant main effects of manure type and moisture condition, as well as a significant interaction between the two factors (Figure 1B). During the dry period (T0–T1), microbial diversity was significantly higher in goat than dairy cow manure, with estimated marginal means of 4.69 and 4.51, respectively (*p* = 0.0012). This difference, however, was no longer evident under wet conditions (T2–T3), where diversity in goat and cow manure was 4.32 and 4.30, respectively (*p* = 0.73). Both manure types exhibited a reduction in diversity during the transition from dry to wet conditions. Still, the decline in goat manure was more pronounced, as indicated by the significant negative interaction term (*β* = −0.166, *t* = −2.32). These findings suggest that goat manure initially supports a more diverse microbial community, but this advantage is diminished under high‐moisture conditions. The chamber effect was negligible, indicating consistent patterns across the experimental replicates.

Further analysis using Principal Coordinates Analysis (PCoA) with Bray–Curtis dissimilarity (Figure 2) confirmed clear separation in microbial community composition between dairy cow and goat manure, primarily along PCo1 (38.7% variance), with dairy cow and goat forming distinct clusters. The moisture period further structured the communities, particularly along the PCo2 axis (19.1% variance). These patterns match the PERMANOVA results, which confirmed significant impacts of manure type (*R* ^2^ = 29.2%, *p* = 0.001), moisture condition (*R* ^2^ = 7.9%, *p* = 0.001), and their interaction (*R* ^2^ = 2.2%, *p* = 0.001) on microbial composition (Table S1). Notably, the change in moisture conditions and the PCoA dispersal are larger in dairy cow manure than in goat manure, indicating lower compositional stability after rainfall simulation in dairy cows. Multivariate dispersion tests (betadisper) corroborated this, showing significant differences in variability between manure types (*p* = 0.002) and for the interaction between manure and period (*p* = 0.001). In contrast, the moisture period alone did not differ in dispersion (*p* = 0.318) (Table S1). Overall, these results suggest that manure type is the dominant, consistent driver of community structure, while moisture's influence is context‐dependent and interacts with substrate‐specific traits to shape community variability.

**FIGURE 2:**
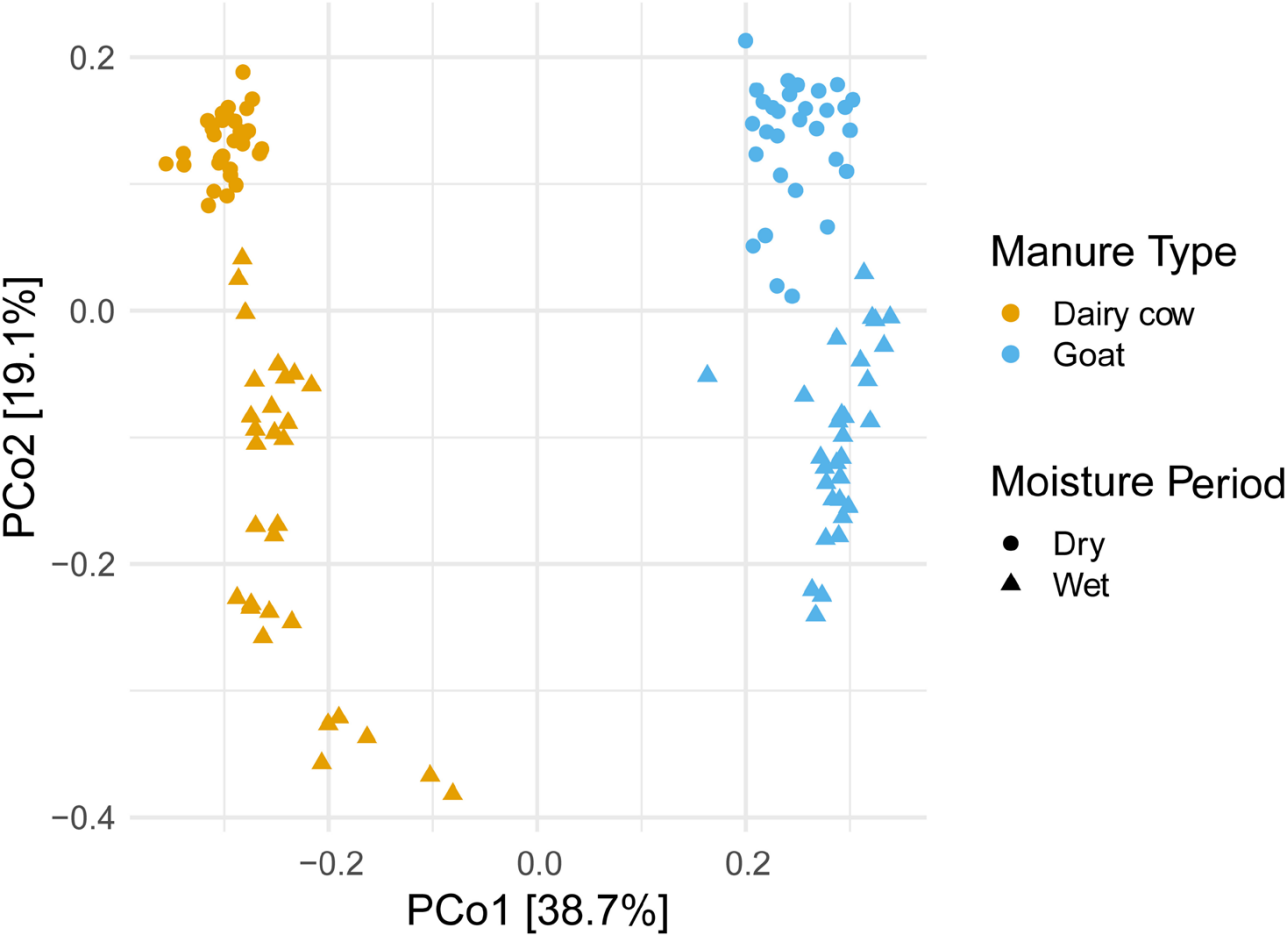
Principal coordinates analysis (PCoA) of microbial community composition in dairy cow and goat manure across dry and wet periods, based on Bray–Curtis dissimilarity. Each point represents a sample, coloured by manure type and shaped according to the moisture period. Axis labels indicate the percentage of variance explained by each principal coordinate (PCo1: 38.7%, PCo2: 19.1%).

Additionally, to quantify the differences observed, we performed a Venn diagram analysis. Venn diagrams highlight distinct retention vs. turnover patterns between periods for the two manure types (Figure 3). In dairy cow manure (Figure 3A), 373 ASVs were shared, with 615 dry‐only and 378 wet‐only ASVs (total = 1366). In goat manure (Figure 3B), overall richness was higher (total = 2013), with 504 shared, 1046 dry‐only, and 463 wet‐only ASVs. Thus, both manures retain a comparable core (shared ≈25%–27% of ASVs), but goat manure showed a broader baseline pool (more dry‐only ASVs; ~52% of total), consistent with higher diversity under dry conditions. By contrast, dairy cows exhibit a relatively larger wet‐only fraction (28% vs. 23% in goat manure), consistent with the greater dry to wet compositional shift observed in the PCoA. Together, these patterns suggest (i) goat manure harbours greater baseline richness but a large dry‐restricted contingent, and (ii) rainfall simulation restructures dairy cow communities more strongly, increasing the set of ASVs detectable only under wet conditions. The complete list of shared and unique ASVs, together with their taxonomic assignments, is provided in Table S2.

**FIGURE 3:**
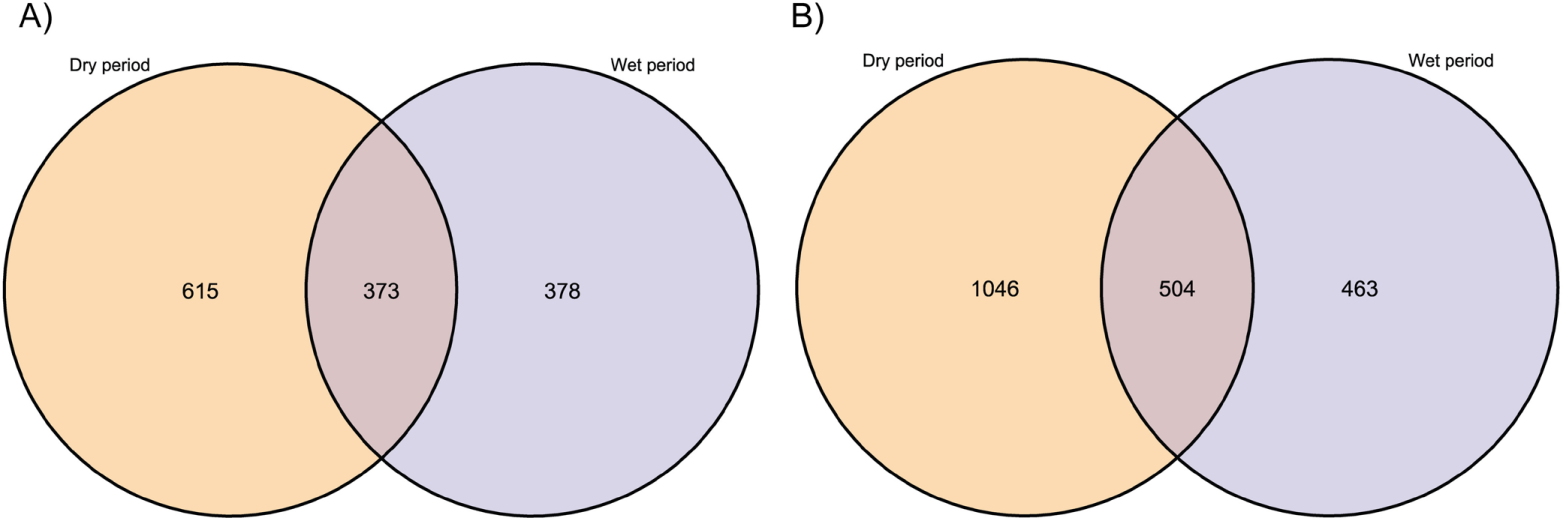
Venn diagrams showing the number of shared and unique ASVs between dry and wet periods in (A) Dairy cow and (B) Goat manure. The central overlap represents ASVs detected in both moisture conditions, while non‐overlapping regions indicate ASVs exclusive to either the dry or wet period. Supplementary Table 2 shows the corresponding ASVs and their taxonomy.

To determine which ASVs persist across varying moisture conditions and their significance within the community, we calculated the cumulative relative abundance of shared ASVs, reflecting the overall proportion of the microbial community comprised of ASVs present during both dry and wet periods. This metric enables us to evaluate whether the shared taxa remain dominant in the community or are displaced by taxa specific to the prevailing conditions after moisture fluctuations. In dairy cow manure (Figure 4A), shared ASVs represented a notably larger fraction of the community in dry conditions (mean = 88.6%) compared to wet conditions (mean = 81.6%, *p* = 0.043). Furthermore, this proportion was considerably more stable across samples during the dry period, while it showed significant variability in wet conditions. These results suggest that although numerous taxa persisted across different moisture conditions, their relative abundance and ecological impact can change substantially following rainfall simulation. This highlights that shifts in community composition due to moisture variation are influenced not just by the loss or addition of taxa but also by changes in the abundance and dominance of enduring members.

**FIGURE 4:**
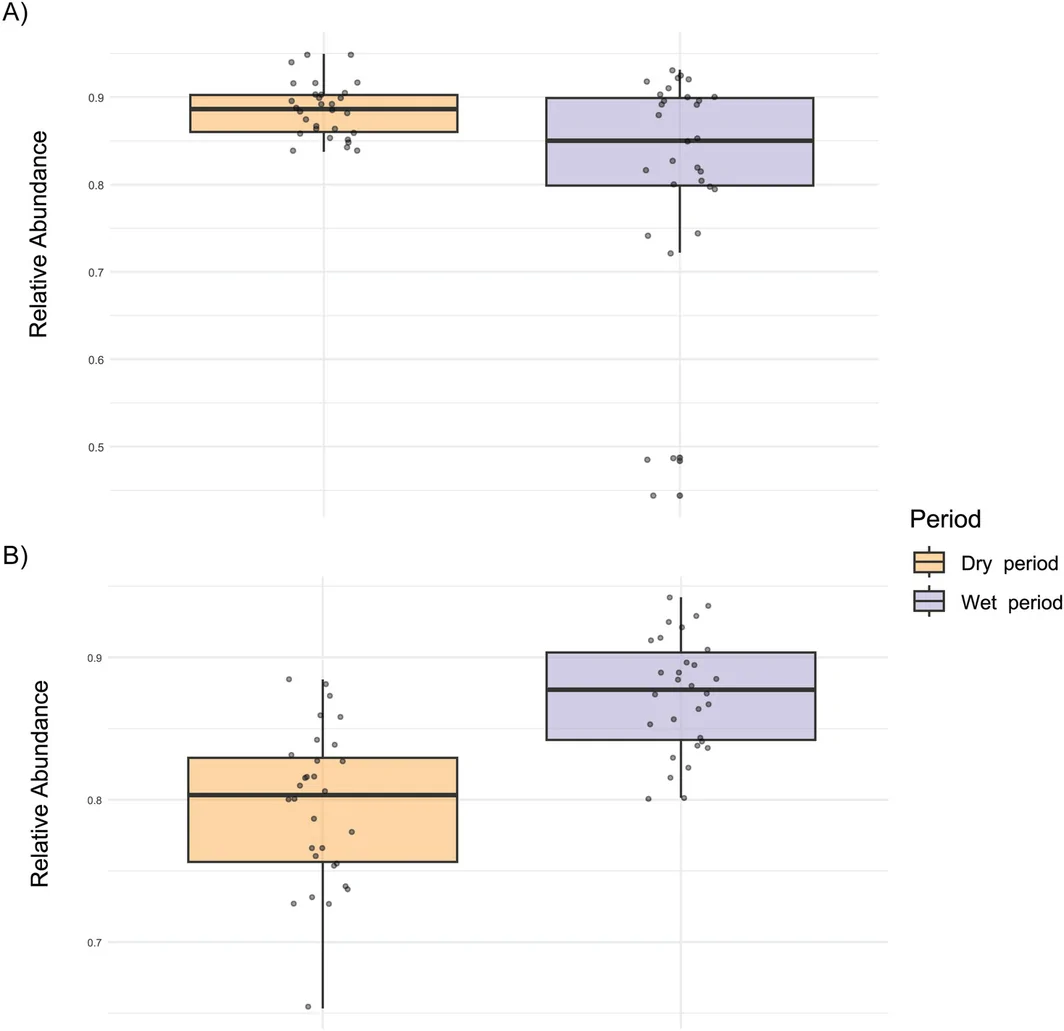
Contrasting persistence of shared ASVs in dairy cow and goat manure across dry and wet periods. (A) Cumulative relative abundance of shared ASVs between dry and wet periods in dairy cow manure. Each point represents the relative abundance of ASVs detected in both periods within an individual sample. Shared ASVs contributed a significantly larger and more consistent portion of the microbial community under dry conditions (mean = 88.6%) than under wet conditions (mean = 81.6%; *p* = 0.043), indicating that rainfall simulation reduced the dominance of persistent taxa and increased community structural variability. (B) Cumulative relative abundance of ASVs shared between dry and wet periods in goat manure. Each point represents the relative abundance of shared ASVs within a single sample. Shared ASVs contributed significantly more to the microbial community in wet samples (mean = 87.4%) compared to dry samples (mean = 79.5%; *p* < 0.000001).

In goat manure (Figure 4B), the overall relative abundance of ASVs shared between dry and wet periods was notably greater in wet samples (mean = 87.4%) compared to dry samples (mean = 79.5%; *p* < 0.001). This suggests that shared ASVs gained dominance after rainfall simulation, leading to a more consistent relative contribution to the community across samples. Therefore, the addition of moisture to goat manure appears to promote a more uniform and stronger community already present in dry conditions, likely indicating an environment‐driven convergence in community structure.

Interestingly, this trend differs for dairy cow manure, where shared ASVs were more prevalent and stable under dry conditions but showed reduced consistency after rainfall simulation. These differing reactions across manure types suggest that moisture affects microbial community structure unevenly, depending on the substrate's specific microbial ecology and resource characteristics. In goat manure, moisture may boost the persistence, relative abundance, or competitive edge of core taxa. Conversely, in dairy cow manure, it may disrupt an otherwise stable community by shifting dominance to a more variable set of taxa that respond to moisture changes.

A differential abundance analysis revealed 15 ASVs that were significantly enriched in dairy cow manure following rainfall simulation (Figure 5A). During the dry period, five ASVs showed higher abundance, predominantly linked to the phyla Bacillota, Bacteroidota, and Pseudomonadota. Notably, Solibacillus (ASV365) and UCG‐005 (ASV263) are stress‐tolerant genera frequently found in low‐moisture conditions, reinforcing the idea that the dry phase promotes the growth of oligotrophic or resilient taxa. Conversely, 10 ASVs were markedly enriched during the wet period, with fold changes often exceeding log_2_FC > 10. These ASVs were primarily from the Bacillota and Pseudomonadota, including genera such as Thermobacillus, Melghirimyces, Ornithinibacillus, and Saccharomonospora. These genera are characterised by rapid growth, metabolic adaptability, and the ability to thrive in nutrient‐rich, moist environments, traits commonly associated with copiotrophic taxa. Importantly, several wet‐enriched ASVs belonged to the Bacillaceae family, a group known for its members' capacity for sporulation and metabolic versatility, enabling them to adapt quickly to moisture fluctuations. The observed log_2_ fold changes (up to +28) highlight the strong ecological prevalence of a few taxa following rainfall simulation, which may contribute to the noted decrease in alpha diversity and increase in community heterogeneity. These trends underscore moisture's role as an environmental filter, shaping bacterial dominance hierarchies and modifying community structure and the relative abundance of functional guilds tied to substrate availability and stress resilience.

**FIGURE 5:**
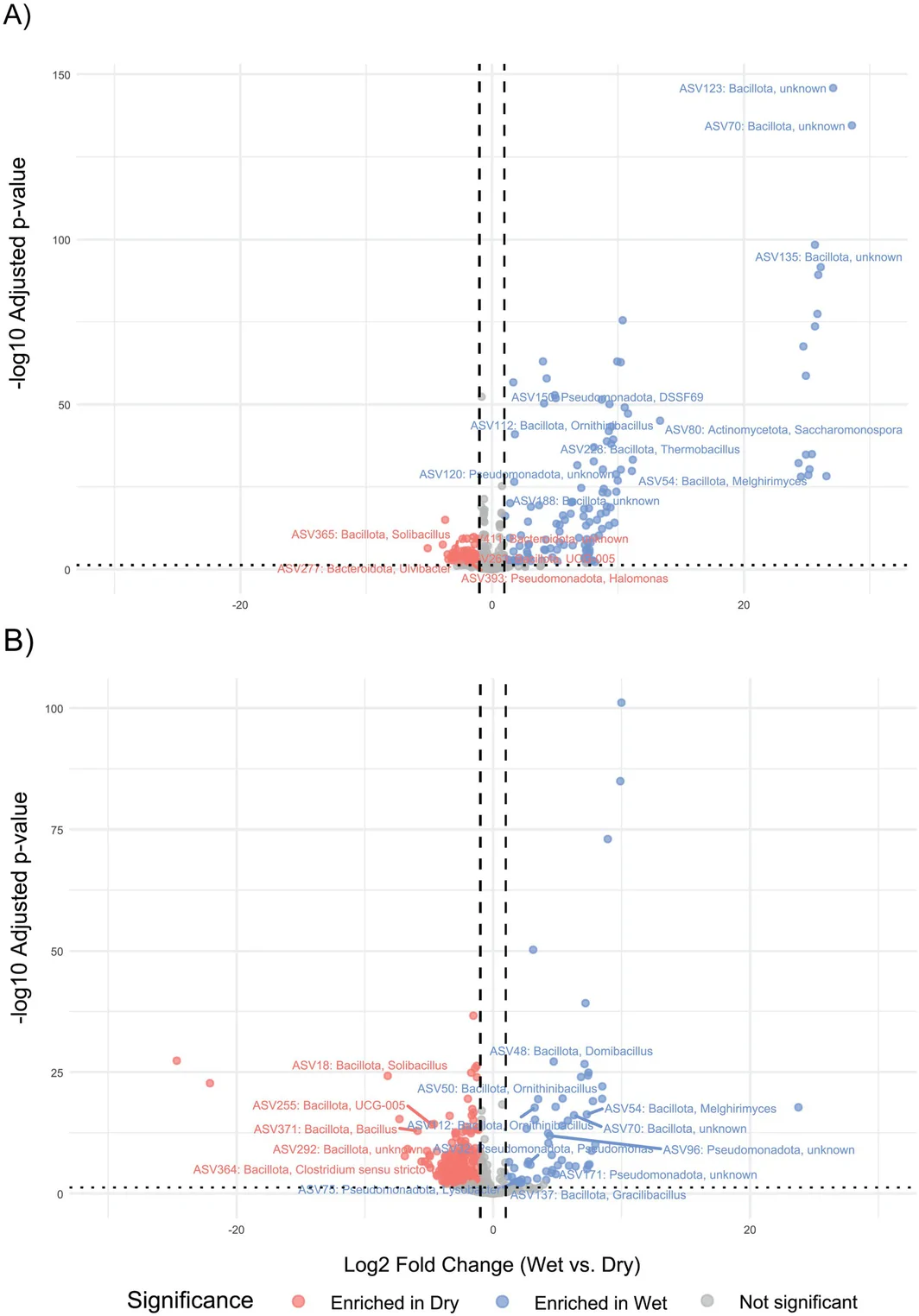
Volcano plot of differential ASV abundance between dry and wet periods in dairy cow and goat manure. Each point represents an ASV, coloured by its statistical significance and enrichment direction. Labelled points denote the most reliable ASVs enriched in either condition (*p*adj < 0.01, |log_2_ fold change| > 2, base mean > 100), with labels showing ASV ID, phylum, and genus. ASVs enriched in the dry period are shown on the left (negative fold change), and those enriched in the wet period on the right. (A) Differential Abundance of Dairy Cow Manure (B) Differential Abundance of Goat Manure.

Goat manure (Figure 5B) exhibited a more conservative response to rainfall simulation compared with dairy cow manure, with overall lower fold changes and fewer taxa showing strong directional shifts. A total of 13 ASVs differed significantly between wet and dry conditions. Of these, seven were enriched under dry conditions, largely affiliated with the *Bacillota* and *Pseudomonadota*. The dominant dry‐enriched genera included *Solibacillus* (ASV18) and *Clostridium sensu stricto 1* (ASV364), both known for endospore formation and desiccation tolerance, suggesting that drought periods favoured taxa adapted to osmotic stress and low substrate turnover. In contrast, six ASVs were enriched following rainfall simulation, mainly belonging to *Bacillota* (e.g., *Dombalicus*, *Meghirimyces*, *Ornithinibacillus*) and *Pseudomonadota*. These moisture‐responsive lineages are associated with rapid microbial restructuring and versatile degradation pathways, consistent with a shift toward copiotrophic dynamics in response to increased moisture. However, compared with cow manure, the magnitude of change (maximum log_2_FC ≈ 12) was smaller, and no single taxon dominated the response, implying a more even microbial restructuring rather than a sharp bloom of specific opportunists. This subdued but coordinated adjustment suggests that the goat‐manure community, initially drier and richer, undergoes a milder compositional reorganisation upon rainfall simulation, is less sensitive to changes in moisture availability, but remains responsive to transient increases in water availability.

To better understand moisture‐related changes in the microbial community, we performed shotgun metagenomic analysis. A targeted differential‐abundance screen identified a focused set of genes that were significantly higher under wet conditions after multiple testing correction (FDR ≤ 0.05), whereas few features were enriched under dry conditions. Many of the wet‐associated signals included resistance/transport functions and locus‐tagged genes, consistent with the rapid growth and stress‐response traits expected under higher hydration (Figure 6). The per‐feature boxplots summarise CPM for each top moisture‐responsive gene across all samples in the dry and wet periods. For most panels, the median and interquartile range shift upward during the wet period, and the *p*‐values printed above each panel indicate significant differences in group means for many genes. We also report Δ, defined as the difference in group means (Δ = mean(wet) − mean(dry), CPM units), which captures the direction and magnitude of the moisture effect. Positive Δ values with small *p*‐values point to genes that are consistently more abundant when moisture is higher, in line with the heatmap's wet‐enriched blocks (Figure 6). These per‐feature contrasts complement the global heatmap by quantifying the direction and magnitude of moisture effects for the genes most strongly associated with the wet period. Overall, many features exhibit higher relative abundance in wet conditions, with a stronger shift in goat, suggesting differential moisture‐associated responses between manure types.

**FIGURE 6:**
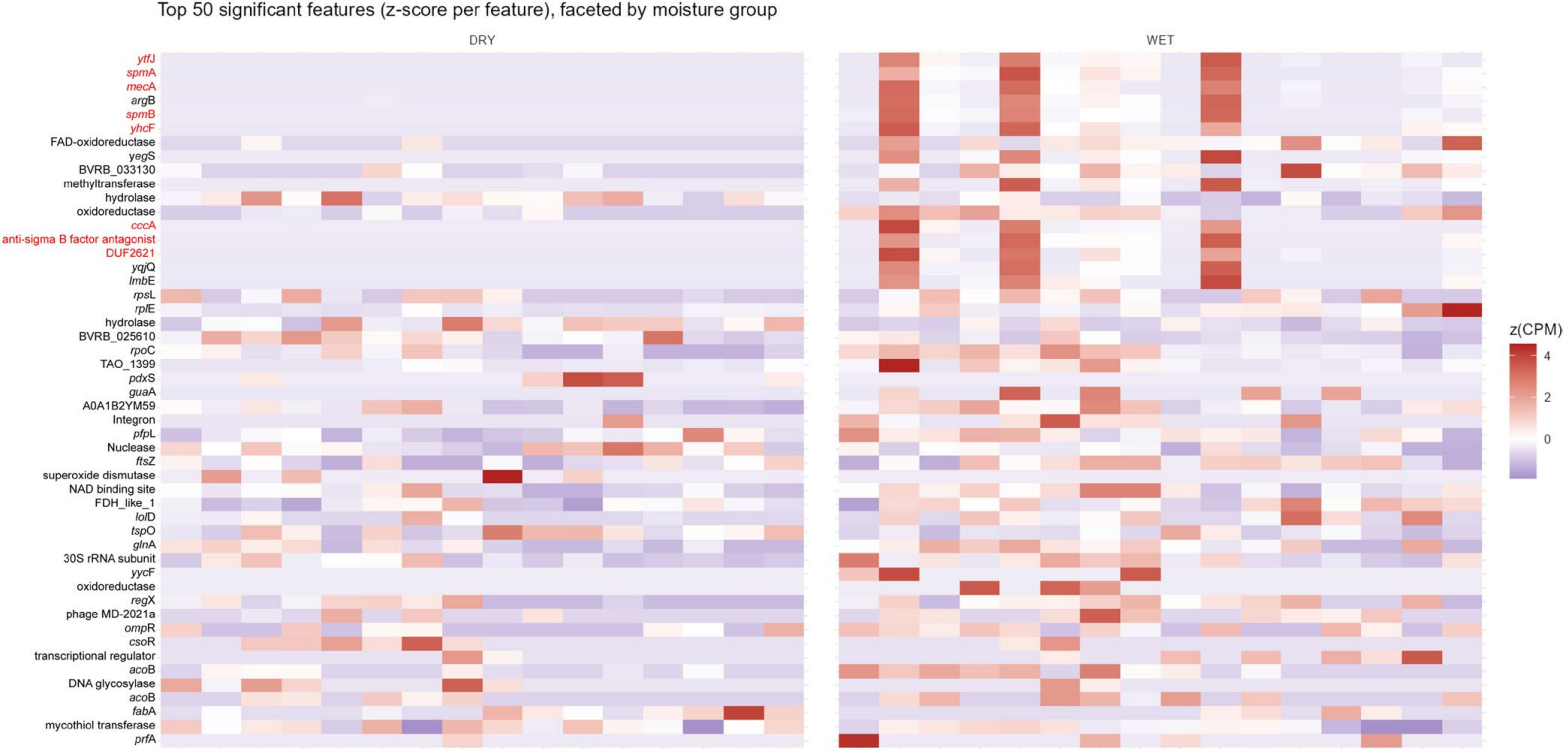
Top LM‐ranked genes across moisture and animals (z‐score heatmap). Rows are the top genes selected by the linear model (ranked by FDR for the moisture main effect); columns are individual samples. Panels are faceted by animal (top: cow; bottom: goat) and by moisture (dry on the left block, wet on the right block). For each feature, CPM values were *z*‐scored within the row; colours show relative abundance (red = above the feature's mean across all samples, white = near mean, light purple = below mean). The column order within each facet is determined by total abundance. Interpretation should be made within rows (not across rows), comparing dry versus wet periods per animal to assess moisture effects, and comparing animals to assess animal effects/interaction. In red are the genes that are important throughout the paper.

Across the top‐ranked genes identified, wet period samples generally show higher CPM than dry period samples within each animal (Figure 7, Table S3), consistent with a strong main effect of moisture. The magnitude of this dry to wet period increase is often larger in goats than in dairy cows, indicating a moisture by animal interaction for a subset of features (Figures 7 and 8). Notably, high abundance does not imply differential change; several abundant panels show overlapping distributions of dry/wet periods, underscoring that some features remain comparatively stable across moisture conditions.

**FIGURE 7:**
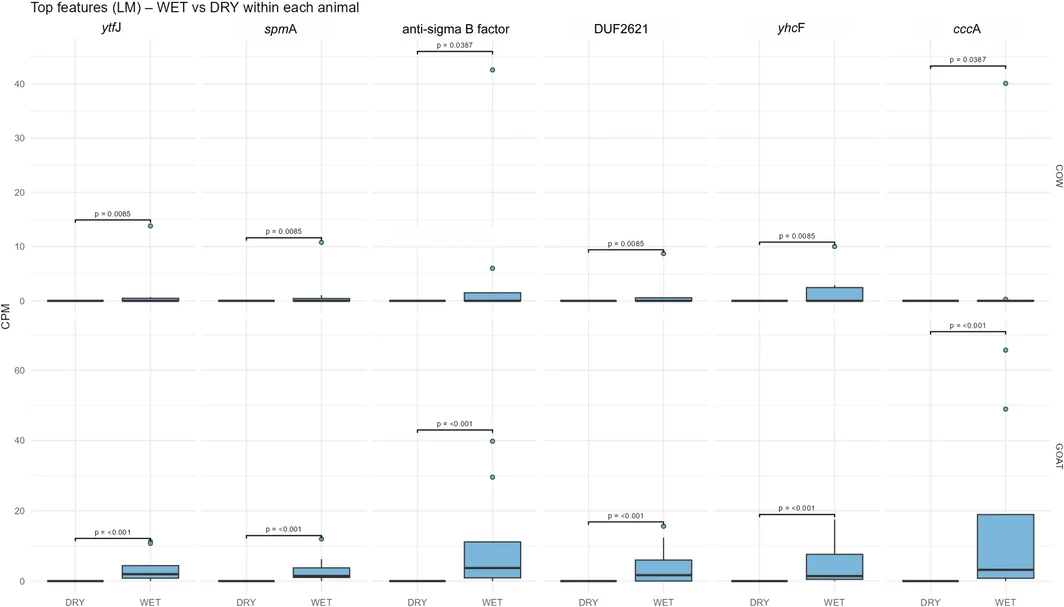
Dry versus wet period gene abundance by animal (boxplots). Panels (columns) show individual top‐ranked genes; rows facet by animal (top: dairy cow, bottom: goat). For each feature, CPM values across replicates are plotted for dry and wet periods: the line is the median, boxes are the interquartile range (Q1–Q3), whiskers extend to 1.5× IQR, and points denote outliers. *Y*‐axes use free scales per panel, interpreted by comparing dry versus wet periods within the same panel, not across panels.

**FIGURE 8:**
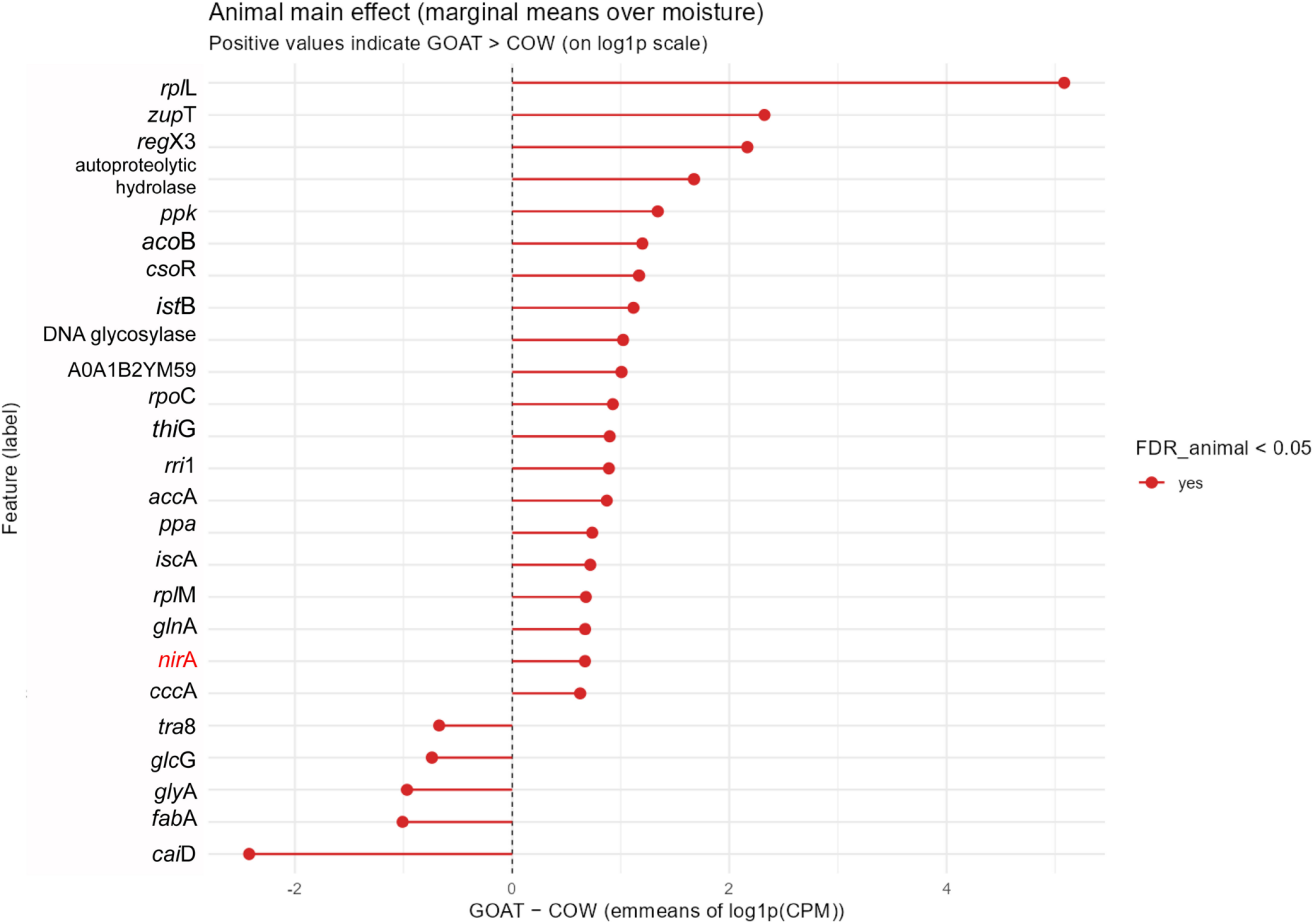
Main effect of animals on gene abundance (Goat – Cow, LM marginal means). Each dot/segment represents one protein (facet labels on the left). The x‐axis is the difference in linear‐model marginal means (averaged over moisture) on the log1p(CPM) scale: Values > 0 indicate the protein is higher overall in goat than in dairy cow; values < 0 indicate higher in dairy cow. Segments extend from 0 to the observed difference; the vertical dashed line marks no animal difference. Points are coloured by animal main‐effect significance (red = FDR_animal < 0.05, grey = not significant). Features are ordered by the magnitude of the goat to cow difference, emphasising the strongest animal biases (e.g., *rpl*L, *zup*T, *reg*X3 toward goat; *fab*A, *gly*A toward dairy cow). In red is the gene that was not identified in the previous analyses.

To summarise the animal main effect, a goat–cow lollipop of LM marginal means (averaged over moisture) was performed. This analysis reveals that the most significant differences favour goats, with the largest positive offsets for genes such as *rpl*L, *zup*T, *reg*X3, AUC31_04925, and *ppk*; a smaller set (e.g., A6048_04875 (*cai*D), *fab*A—indirectly heat‐responsive gene, *gly*A) exhibits higher relative abundance in dairy cows (Figure 8, Table S3). The assimilatory nitrite reductase *nir*A displayed a significant animal main effect, being more abundant in goats than in dairy cows (FDR_animal < 0.05) (Figure 8). Together, these patterns indicate that moisture generally increases gene abundance, with animal‐specific modulation of effect size, while a subset of genes shows minimal change across conditions.

Overall, the heatmaps, boxplots and goat to cow contrasts quantify gene‐level responses to moisture and pinpoint the functions most sensitive to hydration during early‐stage manure storage. The results support the central principle that hydration drives microbial restructuring, shaping both community composition and inferred functional potential, with wet period conditions favouring functions associated with copiotrophic taxa and stress‐response capacities. Collectively, the figures quantify feature‐specific responses to hydration and identify candidate functions most moisture‐sensitive during early‐stage manure storage. Together with the animal manure microbial differences, they support the premise that hydration is a strong ecological filter, shaping both community composition and inferred functional potential, with wet conditions favouring copiotrophic and stress‐response capacities.

## 4 Discussion

This study provides new insights into how short‐term moisture perturbations influence microbial community structure and function in stored manures. Simulated rainfall triggered strong ecological filtering, reducing diversity while enriching spore‐forming, copiotrophic, and moisture‐tolerant taxa. At the same time, functional shifts included enrichment of stress‐ and resistance‐associated genes. In the following discussion, we interpret these findings in the context of microbial life‐history strategies, substrate and host differences, and the implications for emission dynamics under Mediterranean rainfall regimes.

While soil and manure are distinct environments and should not be quantitatively compared, our results show parallel responses to moisture. Specifically, a rainfall simulation pulse acts as a strong environmental filter, decreasing diversity and promoting the growth of fast‐growing, moisture‐adapted taxa, a qualitative pattern also observed in soils. The increased number of unique ASVs in dry goat manure aligns with the alpha‐diversity findings, where goat manure exhibited notably higher Shannon diversity than dairy cow manure during dry periods, consistent with reports that substrate composition and fibre‐rich inputs can support broader niche space and higher baseline richness in ruminant manures (Wan et al. 2021). Rainfall simulation in both types of manure leads to a significant drop in shared ASVs and overall richness, implying that adding moisture creates a strong environmental filter, favouring a narrower range of moisture‐tolerant or copiotrophic taxa (Meng et al. 2019; Ge et al. 2022; Wang et al. 2022; Aguilar‐Paredes et al. 2023; Fu et al. 2023). This filtering is consistent with the observed decrease in alpha diversity in wet conditions, as functionally redundant or competitively dominant taxa become more prevalent. In contrast, more specialised or stress‐adapted taxa are lost (Ge et al. 2022).

Moisture altered the abundance of specific functional genes, suggesting changes in the microbiome's potential activities under wet versus dry conditions. These patterns mirror the community shifts reported earlier (reduced Shannon diversity after rainfall simulation; PERMANOVA separation by moisture), suggesting that rehydration not only reorganises taxonomic composition but also concentrates inferred functional potential into a smaller set of moisture‐associated functions. Across animals, rainfall simulation acted as a strong ecological filter, shifting both taxonomic composition and functional potential. The boxplots and heatmaps show consistent increases in wetness over dryness for many genes, with a larger dry‐to‐wet separation in goat, indicating that animal manure type modulates moisture‐driven functional shifts. This pattern aligns with work on soil, showing that drying to rewetting events reconfigure microbial communities and inferred functional potential, favouring faster growth and altered resource use when water becomes available (Meisner et al. 2021; Zhu et al. 2023).

As several soil studies suggest, the decrease in Shannon diversity observed when transitioning from dry to wet conditions in both manure types is consistent with moisture‐driven environmental filtering. Specifically, water addition can alter oxygen availability, solubility, and redox state, selecting for taxa that tolerate (or exploit) wetter, lower‐oxygen microhabitats (Pett‐Ridge and Firestone 2005; Weil et al. 2020; Siedt et al. 2023). After water addition, community structure often shifts and richness/evenness can decline relative to constant moisture, reflecting the dominance of a subset of moisture‐adapted taxa (Wang et al. 2022; Nie et al. 2025).

Additionally, under dry conditions, goat manure showed higher microbial diversity than dairy cow manure, possibly because its more fibrous and compositionally diverse substrate creates a wider array of ecological niches for ruminant gut microbes. Goats typically consume more fibrous plant material, resulting in manure with a richer niche environment, which promotes the coexistence of a diverse range of microbial taxa. Several studies have discussed how the chemical and physical substrate properties of different animal manures influence their ecological roles, and that mammalian gut microbiomes, and thus their faecal communities, differ substantially due to host evolution, diet, and digestive physiology, justifying our initial difference in diversity between cow and goat manure (Ley et al. 2008; Wan et al. 2021). As mentioned earlier, introducing water during rainfall simulation is likely to alter critical environmental factors, such as oxygen levels, substrate solubility, and redox potential. This can spike respiration due to a surge in CO_2_, known as the Birch effect, which selects for fast‐growing, moisture‐adapted taxa, including copiotrophs, while decreasing community evenness. This reduction could also imply the preferential enrichment of a limited group of previously low‐abundance taxa that can grow quickly in high‐moisture environments, thus resulting in the competitive exclusion of slower‐growing species (Cordero et al. 2023; Singh et al. 2023; Smith et al. 2023). Together, these observations support the view that the physical and chemical properties of the manure matrix set the baseline diversity under dry conditions, whereas moisture acts as a strong ecological filter that overrides initial differences by favouring moisture‐adapted, rapidly responding taxa (Weil et al. 2020).

As shown above, Bacillota was consistently dominant across both manure types (Figure 1A). Differential‐abundance analysis further showed that several Bacillota ASVs were significantly enriched following rainfall simulation, particularly in dairy cow manure (Figure 5A), indicating a marked moisture‐associated shift in the relative abundance of specific members of this phylum. Metagenomic data confirmed this functional shift, where several spore‐associated (i.e., important in spore heat resistance) genes, *spm*A, *spm*B, and *ytf*J, were significantly enriched in the wet period (FDR ≤ 0.05), consistent with a greater contribution of spore‐forming *Firmicutes* (Bacillota) under wetter conditions (Figure 6). While sporulation is classically viewed as a response to stress, spore‐forming *Firmicutes* carry a conserved sporulation gene set and can toggle between vegetative growth and sporulation depending on environmental cues; thus, the observed wet period enrichment likely reflects active life‐cycle dynamics of spore formers during early manure storage rather than a simple ‘dry‐stress only’ response (Filippidou et al. 2016; Galperin et al. 2022). More specifically, Filippidou et al. (2016) have identified direct links between extreme environmental conditions (such as desiccation and water addition) and the prevalence of endospore‐forming *Firmicutes*, establishing the mechanism behind their dominance in our manure storage system. More recently, Galperin et al. (2022) provided a molecular context by detailing the sporulation gene set (including the *spm* and *ytf* genes detected in this study), thereby reinforcing the functional capability of the enriched taxa.

We also observed significant enrichment of *mec*A during wet periods (FDR ≤ 0.05; Figure 6). Additional sequence and conserved‐domain analyses identified this gene as a MecA genetic competence negative regulator (PRK02899), a regulatory protein involved in competence development and stress adaptation in Firmicutes, rather than the classical methicillin‐resistance determinant described in Staphylococcus species (Hahn et al. 1998; Persuh et al. 2002). Its enrichment under wet conditions is therefore more consistent with moisture‐associated regulatory and adaptive responses accompanying microbial community restructuring following rainfall simulation, as illustrated in Figure 6.

Several goat‐biased genes in the lollipop analysis (e.g., *zup*T, *ppk*, *regX*3, *rpl*L) indicate metal and phosphate homeostasis, energy buffering, and translational capacity that differ by manure substrate. Notably, RegX3 is a phosphate‐responsive regulator in *Actinobacteria/Mycobacteria*; its signal here suggests phosphate‐linked regulation may vary with manure type and moisture conditions (Park et al. 2019; Mahatha et al. 2020).

The indicated goat favoured the abundance of *nir*A, suggesting an animal‐specific modulation. Biochemically, NirA is a cytosolic, ferredoxin‐ (or NAD(P)H‐) dependent assimilatory nitrite reductase that generates NH_4_^+^ for biosynthesis, distinguishing it from respiratory (denitrifying) nitrite reductases (Moreno‐Vivián et al. 1999; Fenn et al. 2021; Besson et al. 2022). The observed goat bias and the general wet over dry pattern for many proteins are consistent with well‐known rewetting pulses that mobilise inorganic N and are associated with increased microbial nitrogen assimilation potential in rewetted substrates (Fierer and Schimel 2002; Lu et al. 2020; Wang et al. 2022).

A limitation of the present study is that shotgun metagenomic sequencing provides information regarding inferred functional potential rather than direct microbial activity or gene expression. Consequently, interpretations of microbial function and ecological adaptation should be viewed as associations that reflect contrasting moisture conditions rather than as direct measurements of metabolic processes. In addition, detailed physicochemical assessment associated with this experimental setup is being evaluated separately in a companion emissions‐focused study, limiting direct assessment of how moisture‐mediated physicochemical changes contributed to the observed microbial restructuring patterns (Philippou et al., forthcoming).

In summary, our results demonstrate that even brief rainfall events can rapidly reshape the microbial ecology of stored manures, acting as a pulse disturbance that reorganises both taxonomic and functional dimensions of the microbiome. The transition from dry to wet conditions drives a predictable sequence of responses: loss of diversity through environmental filtering, enrichment of sporulation‐associated and stress‐response‐related functions, and reconfiguration of carbon‐ and nitrogen‐cycling pathways. While these dynamics differ in magnitude between cow and goat substrates, their direction is consistent, highlighting moisture as a universal driver of microbial restructuring across ruminant manures. Together, these findings suggest that transient rewetting pulses disproportionately affect microbial resilience and biogeochemical function, with implications for greenhouse‐gas mitigation and managing moisture exposure during manure storage in seasonally dry climates.

## 5 Conclusion

This study provides insight into how initial substrate heterogeneity and moisture conditions are associated with microbial community restructuring during early‐stage manure storage. We demonstrated that initial microbial diversity and composition are influenced by the physicochemical properties of manure, with more complex, fibrous goat manure supporting higher initial diversity than dairy cow manure. However, the subsequent rainfall simulation acted as a major environmental driver, driving community convergence in both groups through significant enrichment of spore‐forming Bacillota. This enrichment of stress‐tolerant copiotrophs, supported by increased abundance of sporulation‐associated and stress‐response‐related genes, reflects patterns consistent with adaptive responses to moisture fluctuations. Ultimately, this research reveals that the substrate origin significantly dampened the compositional shift following rainfall simulation, with the goat manure community exhibiting superior homeostasis (resilience) compared to the cow manure. Optimising the efficacy and stability of large‐scale manure storage systems requires new models that integrate the physicochemical properties of the starting feedstock, the functional traits of the microbial community, and dynamic moisture conditions. Future investigation should therefore focus on functionally linking these stability metrics to carbon and nutrient cycling rates to optimise moisture management protocols and deploy bioaugmentation management strategies.

## Author Contributions

**P. Pissaridou:** data curation, formal analysis, investigation, validation, writing – original draft. **K. Philippou:** investigation, methodology, writing – review and editing. **A. Phokas:** investigation, writing – review and editing. **J. Rodosthenous:** investigation, writing – review and editing. **C. Hadjimichael:** investigation, writing – review and editing. **M. Omirou:** conceptualization, investigation, visualization, project administration, funding acquisition, writing – original draft, writing – review and editing.

## Funding

This work was supported by the European Union in the framework of NextGenerationEU (C2.1I8).

## Conflicts of Interest

The authors declare no conflicts of interest.

## Supporting information


**Table S1:** Summary of statistical analysis.
**Table S2:** ASV table for Venn diagram analysis.
**Table S3:** Functional annotation of significant genes (Figures 8–10).

## Data Availability

Raw reads were deposited and are publicly available in the NCBI Sequence Read Archive (SRA) under the BioProject accession number PRJNA1354638.
