# Supplementary material for: Rainfall and Substrate Origin Driven Change of Ruminant Manure Microbiomes in Cyprus
Source: Environ Microbiol Rep. 2026 Oct 6;18(5):e70429. doi: 10.1111/1758-2229.70429 (PMC13641882; doi:10.1111/1758-2229.70429)
Supplement: Supplementary file 1 [file EMI4-18-e70429-s001.docx]

**Supplementary Methods**

*SM1.1 Sample collection and processing*

Manure samples from a depth of 0-20 cm were collected from four locations within each manure-filled box using a spatula and sealed in sterile plastic bags at four distinct time points: prior to the experiment (Day 0, T0), before rainfall simulation (Day 14, T1), after rainfall simulation (Day 18, T2) where 4L of water were added, and at the end of the experiment (Day 27, T3). During the rainfall simulation event, 4 L of water were uniformly applied to each manure-filled box to simulate an episodic summer rainfall event representative of semi-arid Mediterranean conditions. The wet period sampling was performed after 3 days to allow the microbial community's response to develop. Therefore, 1 composite sample was collected per chamber/time, and 4 biological replicates were collected for each animal type. Samples were then homogenised in a food blender and passed through a sieve (2 mm). At each sampling time point, one composite sample was collected from each independent manure chamber. Four independent biological replicate chambers were used per manure type, resulting in four biological replicates for each animal × timepoint combination. Rainfall simulation treatments were applied uniformly across chambers using a standardised simulated summer rainfall protocol designed to reflect episodic precipitation events typical of semi-arid Mediterranean conditions.

For downstream analyses, sampling points T0–T1 were classified as the dry period, whereas T2–T3 were classified as the wet period following simulated rainfall application.

In parallel with the microbiome analyses presented here, physicochemical and gaseous emission measurements associated with the same experimental platform were also monitored as part of a companion study focused on greenhouse gas and ammonia emissions under simulated rainfall conditions. These analyses included physicochemical characterisation and environmental monitoring relevant to manure moisture dynamics during storage (Philippou Katerina *et al.*, 2026).

*SM1.2 Manure DNA Extraction and Quantification*

For DNA isolation, homogenised manure samples were spread on clean paper and divided into four quarters. Aliquots from each quarter were thoroughly mixed and placed in 1.5 mL microcentrifuge tubes for DNA extraction. Three replicates were collected from each sample. The remaining sample material was placed in 50 mL centrifuge tubes and stored at -20 °C. The QIAcube IRT protocols for the DNeasy PowerSoil Pro Kit (Qiagen) were used for the DNA extraction. Briefly, a 150 mg manure sample was homogenised in 1 mL lysis buffer CD1 using the Qiagen TissueLyser II for 13 mins at 20 Hz/sec, centrifuged at 15,000 x g for 1 min, and 600 µL of supernatant was then processed with the automated QIAcube® instrument as per manufacturer’s instructions. DNA was eluted in 100 μL buffer AE and stored at -20 °C. Three DNA samples were isolated from each chamber. DNA concentration was determined using the Quant-iT™ PicoGreen™ dsDNA Assay Kit (P7589, Thermo Scientific, USA) as per the manufacturer’s instructions, and DNA quality was assessed by reading absorbance ratios at 230, 260 and 280 nm in NanodropTM ND-2000 (Thermo Scientific, USA).

*SM1.3 DNA library preparation and Next Generation Sequencing*

The bacterial community composition was analysed by amplifying and sequencing the hypervariable V3-V4 region of the 16S rRNA gene using the primer set Forward (5'-TCGTCGGCAGCGTCAGATGTGTATAAGAGACAGCCTACGGGNGGCWGCAG-3') & Reverse (5’-GTCTCGTGGGCTCGGAGATGTGTATAAGAGACAGGACTACHVGGGTATCTAATCC-3'). The DNA libraries were prepared according to Illumina's 16S library preparation protocol (Illumina, San Diego, CA, USA). Briefly, the amplicon PCR (first step) consisted of 5 ng/μl DNA, 1 μM of each primer, and 2X KAPA HiFi HotStart ReadyMix. PCR reactions were performed using a Biometra Thermal Cycler (Analytik Jena) under the following conditions: 95 °C for 3 min; 25 cycles of 95 °C for 30 s, 55 °C for 30 s, and 72 °C for 30 s, followed by a final extension at 72 °C for 5 min. The amplification products were purified using the Agencourt AMPure XP system (Beckman Coulter, Brea, CA, USA), according to the manufacturer's instructions. Then, a second PCR was performed to apply dual indices and Illumina sequencing adapters Nextera XT Index Primer (Illumina), according to the 16S Metagenomic Sequencing Library Preparation protocol (PCR conditions: 95 °C for 3 min; 8 cycles of 95 °C for 30 s, 55 °C for 30 s, 72 °C for 30 s and 72 °C for 5 min). The resulting libraries were purified using the Agencourt AMPure XP system (Beckman Coulter, Brea, CA, USA), and quality control was performed on a TapeStation 2200 platform (Agilent Technologies, Santa Clara, CA, USA). Finally, all barcoded libraries were quantified using the Qubit fluorometer (Invitrogen), normalised and pooled equimolarly. A negative control with no DNA template and a mock sample with sixteen known bacteria were also included. Following library preparation, 20 µl of 650 pM 16S library with 10% (v/v) PhiX spike-in library was loaded onto the flow cell from a NextSeq 1000/2000 P1 Reagents 600-cycle kit. Sequencing was performed on the NextSeq 2000 System (Illumina, San Diego, CA, United States).

Shotgun library preparation was prepared from the extracted total environmental DNA (200 ng per sample) using the Illumina DNA Prep (M) Tagmentation (24 samples, IPB) according to the manufacturer's protocol. Briefly, DNA was subjected to enzymatic tagmentation, a process that simultaneously fragments the DNA and adds adapter sequences. The fragmented, adapter-ligated DNA was then amplified via PCR using barcoded index primers to incorporate the full adapter sequence and sample-specific dual indexes, enabling multiplexing. Following amplification, the resulting libraries were purified using AMPure XP beads to remove primer dimers and unbound reagents. The quality and size distribution of the final libraries were assessed using a TapeStation, and concentrations were quantified via Qubit dsDNA High Sensitivity Assay. Libraries were then pooled at equimolar concentrations and sequenced on a NextSeq 2000 platform using a NextSeq 1000/2000 P2 Reagents 300M (600 cycles) kit.

*SM1.4 Bioinformatic Analysis*

16S NGS result analysis was performed using the DADA2 pipeline, which uses the amplicon sequence variants (ASVs) (Callahan *et al.*, 2016, 2017). Briefly, the read quality profiles were evaluated using the plotQualityProfile function. Quality filtering, denoising, merging and removing chimeric sequences were applied to the dataset. Taxonomic assignment was performed using the Silva 138 Database with a bootstrap threshold of 75% (Callahan *et al.*, 2017).

Alpha diversity analysis was performed using the microbiome package. The pime package was used to identify the prevalence of 90% of bacteria in the manure samples relevant to the study's treatments. Beta diversity, calculated using Principal Coordinates Analysis (PCoA) based on Bray–Curtis dissimilarity, was performed with the vegan package and plotted using the phyloseq package (McMurdie and Holmes, 2013). The microeco package was used for differential abundance test and functional prediction (Liu *et al.*, 2021). All analyses were performed using R version 4.3.2 (2023-10-31).

Shotgun result analysis was performed initially using the standard MetaBakery workflow with default pipeline parameters. Raw per-feature abundances (CPM) from HUMAnN were merged with UniProt annotations and curated before inference. Rows flagged as unmapped/unclassified or without a UniProt primary accession were removed; features with missing CPM values or zero variance across samples were excluded from hypothesis testing. Samples were assigned a priori to moisture groups (dry: T0–T1; wet: T2–T3). For each protein (with a unique label), we tested the difference in group means using a two-sample Welch’s t-test (two-sided). We report the unadjusted p-value, the Benjamini–Hochberg FDR across all tested proteins, and the effect size Δ = mean(wet) − mean(dry) (CPM units). For visualisation, boxplots display medians and interquartile ranges with outliers; p-values and Δ are annotated above each panel. Heatmaps were constructed from the top-ranked proteins (lowest FDR), with CPM z-scored per feature to emphasise relative shifts across samples; columns are ordered by group (dry and wet). All analyses were performed in R using the data. table, dplyr, tidyr, ggplot2, scales, and ggsignif (Hadley Wickham, 216AD; Wickham *et al.*, 2019; Ahlmann-Eltze and Patil, 2021; Barrett T *et al.*, 2025; Wickham H *et al.*, 2025).

*SM1.5 Statistical analysis*

All analyses were conducted in R 4.3.2. Data handling and plotting used phyloseq, data.table/dplyr/tidyr/tibble, ggplot2/ggrepel/ggpubr/ggvenn, and modeling packages lme4/lmerTest, emmeans, vegan, DESeq2, and indicspecies. Unless noted, significance was evaluated at p < 0.05 with Benjamini–Hochberg FDR correction where applicable.

A single phyloseq object was used throughout. For the alpha diversity calculations, the Shannon, Observed, and Simpson indices were computed on the rarefied object; Shannon served as the primary endpoint. Differences were tested with a linear mixed-effects model (lme4/lmerTest). Estimated marginal means and Tukey pairwise contrasts were obtained with emmeans; compact letter displays were used for figure annotations.

Beta diversity and ordination on the rarefied object were computed using Bray–Curtis dissimilarities and visualised with Principal Coordinate Analysis (PCoA). Group differences were assessed by PERMANOVA (adonis2, 999 permutations). Multivariate dispersion was evaluated with betadisper and permutest.

Differential abundance was performed separately for each animal on the non-rarefied object. Low-prevalence taxa were filtered (ASV retained if counts > 10 in > 5 samples). A DESeq2 model with design ~ period was fit, and wet vs dry contrasts were extracted. ASVs with padj < 0.01 were deemed significant. Volcano plots displayed log₂FC versus −log₁₀(FDR); stricter cut-offs (e.g., |log₂FC| > 2 with baseMean thresholds) were used only for figure labelling.

Indicator taxa for the four groups were identified using multipatt with 999 permutations. Significant indicators were summarised at α = 0.05; for box-plot displays, we further required a statistic (stat)≥ 0.85 and a mean relative abundance > 1% in the focal group.

HUMAnN gene-family outputs were merged with UniProt annotations using Gene Family as the join key. Duplicated entries within each file were collapsed by summing sample CPMs and keeping the first non-empty annotation fields. The resulting tables were joined and filtered to remove rows with unmapped or incomplete data (i.e., no UniProt accession or missing CPM values). All CPM columns were converted to numeric values; missing or non-numeric entries were set to zero. Only rows with non-zero totals across samples were retained.

For each merged table, the total CPM per gene and per organism was calculated to generate stacked bar plots of sample composition by organism, heatmaps of the top features (row-wise z-scored CPM), and summary pies showing mapped/unmapped proportions, known versus uncharacterized protein names, and the presence of UniProt accessions.

The top-abundant genes were identified by total CPM across all samples or within each genus. Feature-level univariate statistics (non-parametric) were performed for each feature, Wilcoxon rank-sum tests compared wet vs dry groups (all animals pooled). Output metrics included raw p, FDR, median difference, and log₂(mean wet / mean dry). Features were ranked by FDR and effect magnitude. Volcano plots display log₂ fold-change vs. −log₁₀(FDR). Two-factor linear models were fitted using the lm() function. Type II ANOVA (car::Anova) provided p-values for moisture, animal, and their interaction. Post-hoc estimated marginal means (emmeans) were used to compute the contrast wet to dry (moisture main effect) and to estimate animal marginal means averaged over moisture. Corresponding FDRs were reported as FDR_moisture, FDR_animal, and FDR_interaction. Effect direction and magnitude – For significant animal main effects (FDR < 0.05), marginal mean differences (goat − cow) were visualised with lollipop and dumbbell plots. Positive values indicate higher relative abundance in goat manure.

Boxplots (faceted) show CPM distributions for the top features ranked by Wilcoxon or LM FDR, annotated with p-values (ggsignif). Heatmaps display z-scored CPM across samples, faceted by moisture (dry/wet) or by the animal × moisture combination. All feature-wise analyses were corrected using the Benjamini–Hochberg FDR method. Effect sizes are reported as log₂FC (Wilcoxon) or estimated marginal mean differences (LM).

**Supplementary Figures and Tables**

Supplementary Table 1: Summary of Statistical Analysis

| Permutation test for adonis under reduced model | | | | | | | | | | | | | | |
| --- | --- | --- | --- | --- | --- | --- | --- | --- | --- | --- | --- | --- | --- | --- |
| Number of permutations: 999 | | | | | | | | | | | | | | |
| adonis2(formula = bray_dist ~ Animal * Period, data = metadata, permutations = 999, strata = metadata$Chamber) | | | | | | | | | | | | | | |
|  | | | | | | | | | | | | | | |
|  | Df | SumOfSqs | R2 | F value | Pr(>F) |  |  |  |  |  |  |  | |  |
| Model | 3 | 11.702 | 0.39242 | 24.974 | 0.001 | *** |  |  |  |  |  |  | |  |
| Residual | 116 | 18.118 | 0.60758 |  |  |  |  |  |  |  |  |  | |  |
| Total | 119 | 29.82 | 1 |  |  |  |  |  |  |  |  |  | |  |
|  |  |  |  |  |  |  |  |  |  |  |  |  | |  |
| anova(disp_animal) | | | | | | | | | | | | |  | |
| Analysis of Variance Table | | | | | | | | | | | | |  | |
| Response: Distances | | | | | | | | | | | | |  | |
|  |  |  |  |  |  |  |  |  |  |  |  |  | |  |
|  | Df | Df Sum Sq | Mean Sq | F | Pr(>F) |  |  |  |  |  |  |  | |  |
| Groups | 1 | 0.0409 | 0.0409 | 11.561 | 0.00092 | *** |  |  |  |  |  |  | |  |
| Residuals | 118 | 0.41746 | 0.00354 |  |  |  |  |  |  |  |  |  | |  |
|  |  |  |  |  |  |  |  |  |  |  |  |  | |  |
| permutest(disp_animal, permutations = 999) | | | | |  |  |  |  |  |  |  |  | |  |
| Permutation test for homogeneity of multivariate dispersions | | | | | | |  |  |  |  |  |  | |  |
| Number of permutations: 999 | | | | | | |  |  |  |  |  |  | |  |
| Response: Distances | | |  |  |  |  |  |  |  |  |  |  | |  |
|  |  |  |  |  |  |  |  |  |  |  |  |  | |  |
|  | Df | Sum Sq | Mean Sq | F | N.Perm | Pr(>F) |  |  |  |  |  |  | |  |
| Groups | 1 | 0.0409 | 0.0409 | 11.561 | 999 | 0.002 | ** |  |  |  |  |  | |  |
| Residuals | 118 | 0.41746 | 0.00354 |  |  |  |  |  |  |  |  |  | |  |
|  |  |  |  |  |  |  |  |  |  |  |  |  | |  |
| anova(disp_period) | | | | | | | | | | | | | | |
| Analysis of Variance Table | | | | | | | | | | | | | | |
| Response: Distances | | | | | | | | | | | | | | |
|  |  |  |  |  |  |  |  |  |  |  |  |  | |  |
|  | Df | Df Sum Sq | Mean Sq | F | Pr(>F) |  |  |  |  |  |  |  | |  |
| Groups | 1 | 0.002569 | 0.00257 | 1.0617 | 0.3049 |  |  |  |  |  |  |  | |  |
| Residuals | 118 | 0.285544 | 0.00242 |  |  |  |  |  |  |  |  |  | |  |
|  |  |  |  |  |  |  |  |  |  |  |  |  | |  |
| permutest(disp_period, permutations = 999) | | | | | | | | | | | | | | |
| Permutation test for homogeneity of multivariate dispersions | | | | | | | | | | | | | | |
| Number of permutations: 999 | | | | | | | | | | | | | | |
| Response: Distances | | | | | | | | | | | | | | |
|  |  |  |  |  |  |  |  |  |  |  |  |  | |  |
|  | Df | Sum Sq | Mean Sq | F | N.Perm | Pr(>F) |  |  |  |  |  |  | |  |
| Groups | 1 | 0.002569 | 0.00257 | 1.0617 | 999 | 0.318 |  |  |  |  |  |  | |  |
| Residuals | 118 | 0.285544 | 0.00242 |  |  |  |  |  |  |  |  |  | |  |

Supplementary Table 2: ASV table for Venn diagram analysis

| ASV No | In Dry | In Wet | Region | Genus | Family |
| --- | --- | --- | --- | --- | --- |
| ASV1 | TRUE | TRUE | Shared | *Corynebacterium* | Corynebacteriaceae |
| ASV10 | TRUE | TRUE | Shared | *Solibacillus* | Planococcaceae |
| ASV1002 | TRUE | TRUE | Shared | *unknown* | Sphingobacteriaceae |
| ASV1004 | TRUE | TRUE | Shared | *Agathobacter* | Lachnospiraceae |
| ASV1012 | TRUE | TRUE | Shared | *Atopostipes* | Carnobacteriaceae |
| ASV102 | TRUE | TRUE | Shared | *Facklamia* | Aerococcaceae |
| ASV103 | TRUE | TRUE | Shared | *Lysinibacillus* | Planococcaceae |
| ASV104 | TRUE | TRUE | Shared | *Dietzia* | Dietziaceae |
| ASV1041 | TRUE | TRUE | Shared | *Sporosarcina* | Planococcaceae |
| ASV105 | TRUE | TRUE | Shared | *Aliidiomarina* | Idiomarinaceae |
| ASV106 | TRUE | TRUE | Shared | *Romboutsia* | Peptostreptococcaceae |
| ASV1071 | TRUE | TRUE | Shared | *Lysobacter* | Xanthomonadaceae |
| ASV108 | TRUE | TRUE | Shared | *Oceanobacillus* | Bacillaceae |
| ASV1082 | TRUE | TRUE | Shared | *Terrisporobacter* | Peptostreptococcaceae |
| ASV109 | TRUE | TRUE | Shared | *Alkalibacterium* | Carnobacteriaceae |
| ASV1096 | TRUE | TRUE | Shared | *Thiopseudomonas* | Pseudomonadaceae |
| ASV11 | TRUE | TRUE | Shared | *Aliidiomarina* | Idiomarinaceae |
| ASV111 | TRUE | TRUE | Shared | *Tissierella* | Family XI |
| ASV1115 | TRUE | TRUE | Shared | *Pseudactinotalea* | Cellulomonadaceae |
| ASV1127 | TRUE | TRUE | Shared | *Treponema* | Spirochaetaceae |
| ASV114 | TRUE | TRUE | Shared | *Alkalibacterium* | Carnobacteriaceae |
| ASV1141 | TRUE | TRUE | Shared | *Bacteroides* | Bacteroidaceae |
| ASV1147 | TRUE | TRUE | Shared | *Proteiniclasticum* | Clostridiaceae |
| ASV115 | TRUE | TRUE | Shared | *Marinobacter* | Marinobacteraceae |
| ASV1152 | TRUE | TRUE | Shared | *Prevotellaceae UCG-001* | Prevotellaceae |
| ASV116 | TRUE | TRUE | Shared | *unknown* | Fodinicurvataceae |
| ASV118 | TRUE | TRUE | Shared | *unknown* | JG30-KF-CM45 |
| ASV1182 | TRUE | TRUE | Shared | *Bacteroides* | Bacteroidaceae |
| ASV119 | TRUE | TRUE | Shared | *Desemzia* | Carnobacteriaceae |
| ASV12 | TRUE | TRUE | Shared | *Enteractinococcus* | Micrococcaceae |
| ASV121 | TRUE | TRUE | Shared | *Corynebacterium* | Corynebacteriaceae |
| ASV122 | TRUE | TRUE | Shared | *Halomonas* | Halomonadaceae |
| ASV1221 | TRUE | TRUE | Shared | *Marinimicrobium* | Cellvibrionaceae |
| ASV124 | TRUE | TRUE | Shared | *Pseudomonas* | Pseudomonadaceae |
| ASV1251 | TRUE | TRUE | Shared | *Prevotellaceae UCG-004* | Prevotellaceae |
| ASV126 | TRUE | TRUE | Shared | *unknown* | Cyclobacteriaceae |
| ASV128 | TRUE | TRUE | Shared | *Atopostipes* | Carnobacteriaceae |
| ASV1297 | TRUE | TRUE | Shared | *Lacticigenium* | Carnobacteriaceae |
| ASV13 | TRUE | TRUE | Shared | *Jeotgalicoccus* | Staphylococcaceae |
| ASV130 | TRUE | TRUE | Shared | *Escherichia-Shigella* | Enterobacteriaceae |
| ASV132 | TRUE | TRUE | Shared | *Halomonas* | Halomonadaceae |
| ASV136 | TRUE | TRUE | Shared | *unknown* | Micrococcaceae |
| ASV1366 | TRUE | TRUE | Shared | *unknown* | unknown |
| ASV1368 | TRUE | TRUE | Shared | *Trueperella* | Actinomycetaceae |
| ASV138 | TRUE | TRUE | Shared | *unknown* | Xanthomonadaceae |
| ASV139 | TRUE | TRUE | Shared | *Flaviflexus* | Actinomycetaceae |
| ASV1397 | TRUE | TRUE | Shared | *unknown* | Flavobacteriaceae |
| ASV14 | TRUE | TRUE | Shared | *Salinicoccus* | Staphylococcaceae |
| ASV1404 | TRUE | TRUE | Shared | *unknown* | Ruminococcaceae |
| ASV1407 | TRUE | TRUE | Shared | *unknown* | unknown |
| ASV141 | TRUE | TRUE | Shared | *Yaniella* | Micrococcaceae |
| ASV1419 | TRUE | TRUE | Shared | *unknown* | unknown |
| ASV143 | TRUE | TRUE | Shared | *Pseudactinotalea* | Cellulomonadaceae |
| ASV1431 | TRUE | TRUE | Shared | *unknown* | Balneolaceae |
| ASV1444 | TRUE | TRUE | Shared | *unknown* | Sandaracinaceae |
| ASV145 | TRUE | TRUE | Shared | *Corynebacterium* | Corynebacteriaceae |
| ASV147 | TRUE | TRUE | Shared | *Alkalibacterium* | Carnobacteriaceae |
| ASV148 | TRUE | TRUE | Shared | *Solibacillus* | Planococcaceae |
| ASV149 | TRUE | TRUE | Shared | *unknown* | unknown |
| ASV1496 | TRUE | TRUE | Shared | *Acinetobacter* | Moraxellaceae |
| ASV15 | TRUE | TRUE | Shared | *Clostridium sensu stricto 1* | Clostridiaceae |
| ASV1519 | TRUE | TRUE | Shared | *Kurthia* | Planococcaceae |
| ASV1525 | TRUE | TRUE | Shared | *Acinetobacter* | Moraxellaceae |
| ASV1529 | TRUE | TRUE | Shared | *Ercella* | Hungateiclostridiaceae |
| ASV154 | TRUE | TRUE | Shared | *Salinimicrobium* | Flavobacteriaceae |
| ASV1540 | TRUE | TRUE | Shared | *Atopostipes* | Carnobacteriaceae |
| ASV155 | TRUE | TRUE | Shared | *Brachybacterium* | Dermabacteraceae |
| ASV1550 | TRUE | TRUE | Shared | *Leucobacter* | Microbacteriaceae |
| ASV156 | TRUE | TRUE | Shared | *Oceanobacter* | Saccharospirillaceae |
| ASV157 | TRUE | TRUE | Shared | *Aliidiomarina* | Idiomarinaceae |
| ASV158 | TRUE | TRUE | Shared | *Facklamia* | Aerococcaceae |
| ASV16 | TRUE | TRUE | Shared | *Halomonas* | Halomonadaceae |
| ASV160 | TRUE | TRUE | Shared | *Flavobacterium* | Flavobacteriaceae |
| ASV161 | TRUE | TRUE | Shared | *Acinetobacter* | Moraxellaceae |
| ASV1623 | TRUE | TRUE | Shared | *unknown* | UCG-010 |
| ASV1629 | TRUE | TRUE | Shared | *Altererythrobacter* | Sphingomonadaceae |
| ASV163 | TRUE | TRUE | Shared | *Turicibacter* | Erysipelotrichaceae |
| ASV165 | TRUE | TRUE | Shared | *Planococcus* | Planococcaceae |
| ASV166 | TRUE | TRUE | Shared | *Clostridium sensu stricto 1* | Clostridiaceae |
| ASV1679 | TRUE | TRUE | Shared | *Alloprevotella* | Prevotellaceae |
| ASV169 | TRUE | TRUE | Shared | *unknown* | JG30-KF-CM45 |
| ASV1696 | TRUE | TRUE | Shared | *Marvinbryantia* | Lachnospiraceae |
| ASV17 | TRUE | TRUE | Shared | *Marinobacter* | Marinobacteraceae |
| ASV1715 | TRUE | TRUE | Shared | *unknown* | Nocardioidaceae |
| ASV172 | TRUE | TRUE | Shared | *unknown* | Planococcaceae |
| ASV173 | TRUE | TRUE | Shared | *unknown* | Bacillaceae |
| ASV174 | TRUE | TRUE | Shared | *Salinimicrobium* | Flavobacteriaceae |
| ASV175 | TRUE | TRUE | Shared | *Ornithinimicrobium* | Intrasporangiaceae |
| ASV177 | TRUE | TRUE | Shared | *Truepera* | Trueperaceae |
| ASV1774 | TRUE | TRUE | Shared | *Vagococcus* | Vagococcaceae |
| ASV178 | TRUE | TRUE | Shared | *unknown* | Bacillaceae |
| ASV18 | TRUE | TRUE | Shared | *Solibacillus* | Planococcaceae |
| ASV181 | TRUE | TRUE | Shared | *Alkalibacterium* | Carnobacteriaceae |
| ASV1829 | TRUE | TRUE | Shared | *unknown* | unknown |
| ASV184 | TRUE | TRUE | Shared | *unknown* | unknown |
| ASV185 | TRUE | TRUE | Shared | *Aliidiomarina* | Idiomarinaceae |
| ASV1856 | TRUE | TRUE | Shared | *unknown* | unknown |
| ASV186 | TRUE | TRUE | Shared | *unknown* | Planococcaceae |
| ASV187 | TRUE | TRUE | Shared | *Turicibacter* | Erysipelotrichaceae |
| ASV189 | TRUE | TRUE | Shared | *unknown* | Planococcaceae |
| ASV19 | TRUE | TRUE | Shared | *Turicibacter* | Erysipelotrichaceae |
| ASV190 | TRUE | TRUE | Shared | *Solibacillus* | Planococcaceae |
| ASV191 | TRUE | TRUE | Shared | *Ornithinimicrobium* | Intrasporangiaceae |
| ASV194 | TRUE | TRUE | Shared | *unknown* | Planococcaceae |
| ASV199 | TRUE | TRUE | Shared | *Clostridium sensu stricto 1* | Clostridiaceae |
| ASV2 | TRUE | TRUE | Shared | *Salinicoccus* | Staphylococcaceae |
| ASV20 | TRUE | TRUE | Shared | *Paeniclostridium* | Peptostreptococcaceae |
| ASV200 | TRUE | TRUE | Shared | *Treponema* | Spirochaetaceae |
| ASV201 | TRUE | TRUE | Shared | *Acholeplasma* | Acholeplasmataceae |
| ASV2024 | TRUE | TRUE | Shared | *Rikenellaceae RC9 gut group* | Rikenellaceae |
| ASV203 | TRUE | TRUE | Shared | *Aliicoccus* | Staphylococcaceae |
| ASV204 | TRUE | TRUE | Shared | *unknown* | Rhodothermaceae |
| ASV205 | TRUE | TRUE | Shared | *Cellulosilyticum* | Lachnospiraceae |
| ASV206 | TRUE | TRUE | Shared | *Marinimicrobium* | Cellvibrionaceae |
| ASV207 | TRUE | TRUE | Shared | *unknown* | JG30-KF-CM45 |
| ASV208 | TRUE | TRUE | Shared | *Aliidiomarina* | Idiomarinaceae |
| ASV209 | TRUE | TRUE | Shared | *unknown* | Carnobacteriaceae |
| ASV21 | TRUE | TRUE | Shared | *Gracilibacillus* | Bacillaceae |
| ASV211 | TRUE | TRUE | Shared | *unknown* | Flavobacteriaceae |
| ASV215 | TRUE | TRUE | Shared | *Cellvibrio* | Cellvibrionaceae |
| ASV2193 | TRUE | TRUE | Shared | *Acinetobacter* | Moraxellaceae |
| ASV22 | TRUE | TRUE | Shared | *Dietzia* | Dietziaceae |
| ASV222 | TRUE | TRUE | Shared | *Isoptericola* | Promicromonosporaceae |
| ASV225 | TRUE | TRUE | Shared | *unknown* | JG30-KF-CM45 |
| ASV226 | TRUE | TRUE | Shared | *Brumimicrobium* | Crocinitomicaceae |
| ASV23 | TRUE | TRUE | Shared | *Pseudomonas* | Pseudomonadaceae |
| ASV230 | TRUE | TRUE | Shared | *unknown* | JG30-KF-CM45 |
| ASV232 | TRUE | TRUE | Shared | *Flaviflexus* | Actinomycetaceae |
| ASV2329 | TRUE | TRUE | Shared | *unknown* | Sphingobacteriaceae |
| ASV238 | TRUE | TRUE | Shared | *unknown* | Fodinicurvataceae |
| ASV239 | TRUE | TRUE | Shared | *Bacillus* | Bacillaceae |
| ASV24 | TRUE | TRUE | Shared | *Atopostipes* | Carnobacteriaceae |
| ASV241 | TRUE | TRUE | Shared | *Bacteroides* | Bacteroidaceae |
| ASV242 | TRUE | TRUE | Shared | *Marinobacter* | Marinobacteraceae |
| ASV245 | TRUE | TRUE | Shared | *Succinivibrio* | Succinivibrionaceae |
| ASV247 | TRUE | TRUE | Shared | *Aliidiomarina* | Idiomarinaceae |
| ASV25 | TRUE | TRUE | Shared | *Corynebacterium* | Corynebacteriaceae |
| ASV250 | TRUE | TRUE | Shared | *Halomonas* | Halomonadaceae |
| ASV2501 | TRUE | TRUE | Shared | *Lysinibacillus* | Planococcaceae |
| ASV253 | TRUE | TRUE | Shared | *Fastidiosipila* | Hungateiclostridiaceae |
| ASV254 | TRUE | TRUE | Shared | *Pelagibacterium* | Devosiaceae |
| ASV2545 | TRUE | TRUE | Shared | *unknown* | Dysgonomonadaceae |
| ASV2560 | TRUE | TRUE | Shared | *Atopostipes* | Carnobacteriaceae |
| ASV258 | TRUE | TRUE | Shared | *Ulvibacter* | Flavobacteriaceae |
| ASV26 | TRUE | TRUE | Shared | *Corynebacterium* | Corynebacteriaceae |
| ASV261 | TRUE | TRUE | Shared | *Salinicoccus* | Staphylococcaceae |
| ASV263 | TRUE | TRUE | Shared | *UCG-005* | Oscillospiraceae |
| ASV264 | TRUE | TRUE | Shared | *unknown* | Bacteroidales RF16 group |
| ASV267 | TRUE | TRUE | Shared | *Atopostipes* | Carnobacteriaceae |
| ASV268 | TRUE | TRUE | Shared | *Ammoniphilus* | Paenibacillaceae |
| ASV269 | TRUE | TRUE | Shared | *unknown* | Bacillaceae |
| ASV27 | TRUE | TRUE | Shared | *Salinimicrobium* | Flavobacteriaceae |
| ASV270 | TRUE | TRUE | Shared | *Planococcus* | Planococcaceae |
| ASV273 | TRUE | TRUE | Shared | *unknown* | Xanthomonadaceae |
| ASV274 | TRUE | TRUE | Shared | *Iamia* | Iamiaceae |
| ASV275 | TRUE | TRUE | Shared | *Alcanivorax* | Alcanivoracaceae1 |
| ASV277 | TRUE | TRUE | Shared | *Ulvibacter* | Flavobacteriaceae |
| ASV278 | TRUE | TRUE | Shared | *Acinetobacter* | Moraxellaceae |
| ASV28 | TRUE | TRUE | Shared | *Aliicoccus* | Staphylococcaceae |
| ASV280 | TRUE | TRUE | Shared | *Oceanobacillus* | Bacillaceae |
| ASV281 | TRUE | TRUE | Shared | *Solibacillus* | Planococcaceae |
| ASV282 | TRUE | TRUE | Shared | *unknown* | JG30-KF-CM45 |
| ASV283 | TRUE | TRUE | Shared | *Lysinibacillus* | Planococcaceae |
| ASV286 | TRUE | TRUE | Shared | *Corynebacterium* | Corynebacteriaceae |
| ASV288 | TRUE | TRUE | Shared | *Amphibacillus* | Bacillaceae |
| ASV289 | TRUE | TRUE | Shared | *Aequorivita* | Flavobacteriaceae |
| ASV29 | TRUE | TRUE | Shared | *Corynebacterium* | Corynebacteriaceae |
| ASV290 | TRUE | TRUE | Shared | *unknown* | unknown |
| ASV291 | TRUE | TRUE | Shared | *unknown* | unknown |
| ASV293 | TRUE | TRUE | Shared | *Pseudomonas* | Pseudomonadaceae |
| ASV295 | TRUE | TRUE | Shared | *Tessaracoccus* | Propionibacteriaceae |
| ASV299 | TRUE | TRUE | Shared | *unknown* | unknown |
| ASV3 | TRUE | TRUE | Shared | *Garicola* | Micrococcaceae |
| ASV30 | TRUE | TRUE | Shared | *unknown* | Micrococcaceae |
| ASV305 | TRUE | TRUE | Shared | *Eionea* | Cellvibrionaceae |
| ASV308 | TRUE | TRUE | Shared | *Halolactibacillus* | Bacillaceae |
| ASV312 | TRUE | TRUE | Shared | *Amphibacillus* | Bacillaceae |
| ASV318 | TRUE | TRUE | Shared | *unknown* | Balneolaceae |
| ASV32 | TRUE | TRUE | Shared | *Pseudomonas* | Pseudomonadaceae |
| ASV320 | TRUE | TRUE | Shared | *unknown* | Cellvibrionaceae |
| ASV321 | TRUE | TRUE | Shared | *TM7a* | Saccharimonadaceae |
| ASV323 | TRUE | TRUE | Shared | *Bacteroides* | Bacteroidaceae |
| ASV327 | TRUE | TRUE | Shared | *Salipaludibacillus* | Salisediminibacteriaceae |
| ASV328 | TRUE | TRUE | Shared | *unknown* | Cyclobacteriaceae |
| ASV33 | TRUE | TRUE | Shared | *unknown* | Xanthomonadaceae |
| ASV330 | TRUE | TRUE | Shared | *[Ruminococcus] torques group* | Lachnospiraceae |
| ASV332 | TRUE | TRUE | Shared | *Atopostipes* | Carnobacteriaceae |
| ASV338 | TRUE | TRUE | Shared | *Salinicoccus* | Staphylococcaceae |
| ASV339 | TRUE | TRUE | Shared | *Amphibacillus* | Bacillaceae |
| ASV341 | TRUE | TRUE | Shared | *Prevotellaceae UCG-004* | Prevotellaceae |
| ASV342 | TRUE | TRUE | Shared | *unknown* | Guggenheimella |
| ASV343 | TRUE | TRUE | Shared | *unknown* | Cellvibrionaceae |
| ASV345 | TRUE | TRUE | Shared | *UCG-005* | Oscillospiraceae |
| ASV348 | TRUE | TRUE | Shared | *UCG-005* | Oscillospiraceae |
| ASV349 | TRUE | TRUE | Shared | *unknown* | JG30-KF-CM45 |
| ASV35 | TRUE | TRUE | Shared | *Marinimicrobium* | Cellvibrionaceae |
| ASV352 | TRUE | TRUE | Shared | *unknown* | Bacillaceae |
| ASV355 | TRUE | TRUE | Shared | *Altererythrobacter* | Sphingomonadaceae |
| ASV356 | TRUE | TRUE | Shared | *unknown* | unknown |
| ASV36 | TRUE | TRUE | Shared | *Atopostipes* | Carnobacteriaceae |
| ASV37 | TRUE | TRUE | Shared | *Salinicoccus* | Staphylococcaceae |
| ASV373 | TRUE | TRUE | Shared | *unknown* | Bradymonadaceae |
| ASV378 | TRUE | TRUE | Shared | *Phascolarctobacterium* | Acidaminococcaceae |
| ASV38 | TRUE | TRUE | Shared | *Corynebacterium* | Corynebacteriaceae |
| ASV384 | TRUE | TRUE | Shared | *unknown* | Bacillaceae |
| ASV39 | TRUE | TRUE | Shared | *Planococcus* | Planococcaceae |
| ASV390 | TRUE | TRUE | Shared | *Georgenia* | Bogoriellaceae |
| ASV391 | TRUE | TRUE | Shared | *Lysinibacillus* | Planococcaceae |
| ASV392 | TRUE | TRUE | Shared | *Atopostipes* | Carnobacteriaceae |
| ASV393 | TRUE | TRUE | Shared | *Halomonas* | Halomonadaceae |
| ASV394 | TRUE | TRUE | Shared | *Bacteroides* | Bacteroidaceae |
| ASV4 | TRUE | TRUE | Shared | *Salinicoccus* | Staphylococcaceae |
| ASV40 | TRUE | TRUE | Shared | *Enteractinococcus* | Micrococcaceae |
| ASV401 | TRUE | TRUE | Shared | *Oceanobacillus* | Bacillaceae |
| ASV403 | TRUE | TRUE | Shared | *TM7a* | Saccharimonadaceae |
| ASV404 | TRUE | TRUE | Shared | *Ornithinimicrobium* | Intrasporangiaceae |
| ASV407 | TRUE | TRUE | Shared | *Lacticigenium* | Carnobacteriaceae |
| ASV41 | TRUE | TRUE | Shared | *Ornithinimicrobium* | Intrasporangiaceae |
| ASV411 | TRUE | TRUE | Shared | *unknown* | Bacteroidales RF16 group |
| ASV414 | TRUE | TRUE | Shared | *unknown* | unknown |
| ASV415 | TRUE | TRUE | Shared | *Idiomarina* | Idiomarinaceae |
| ASV419 | TRUE | TRUE | Shared | *unknown* | Balneolaceae |
| ASV42 | TRUE | TRUE | Shared | *Dietzia* | Dietziaceae |
| ASV421 | TRUE | TRUE | Shared | *Bhargavaea* | Planococcaceae |
| ASV423 | TRUE | TRUE | Shared | *Truepera* | Trueperaceae |
| ASV427 | TRUE | TRUE | Shared | *unknown* | Marinilabiliaceae |
| ASV428 | TRUE | TRUE | Shared | *Solibacillus* | Planococcaceae |
| ASV429 | TRUE | TRUE | Shared | *UCG-005* | Oscillospiraceae |
| ASV43 | TRUE | TRUE | Shared | *Atopostipes* | Carnobacteriaceae |
| ASV435 | TRUE | TRUE | Shared | *Fermentimonas* | Dysgonomonadaceae |
| ASV44 | TRUE | TRUE | Shared | *Atopostipes* | Carnobacteriaceae |
| ASV441 | TRUE | TRUE | Shared | *unknown* | Planococcaceae |
| ASV442 | TRUE | TRUE | Shared | *unknown* | unknown |
| ASV444 | TRUE | TRUE | Shared | *Nocardiopsis* | Nocardiopsaceae |
| ASV45 | TRUE | TRUE | Shared | *Halomonas* | Halomonadaceae |
| ASV451 | TRUE | TRUE | Shared | *Fastidiosipila* | Hungateiclostridiaceae |
| ASV452 | TRUE | TRUE | Shared | *unknown* | Xanthomonadaceae |
| ASV454 | TRUE | TRUE | Shared | *unknown* | Lachnospiraceae |
| ASV459 | TRUE | TRUE | Shared | *Halolactibacillus* | Bacillaceae |
| ASV46 | TRUE | TRUE | Shared | *unknown* | Carnobacteriaceae |
| ASV460 | TRUE | TRUE | Shared | *Oceanobacillus* | Bacillaceae |
| ASV47 | TRUE | TRUE | Shared | *Alkalibacterium* | Carnobacteriaceae |
| ASV478 | TRUE | TRUE | Shared | *Aequorivita* | Flavobacteriaceae |
| ASV482 | TRUE | TRUE | Shared | *W5053* | Family XI |
| ASV484 | TRUE | TRUE | Shared | *Gracilibacillus* | Bacillaceae |
| ASV4858 | TRUE | TRUE | Shared | *UCG-005* | Oscillospiraceae |
| ASV487 | TRUE | TRUE | Shared | *Prevotellaceae UCG-003* | Prevotellaceae |
| ASV4871 | TRUE | TRUE | Shared | *unknown* | unknown |
| ASV49 | TRUE | TRUE | Shared | *Enteractinococcus* | Micrococcaceae |
| ASV490 | TRUE | TRUE | Shared | *Lysinibacillus* | Planococcaceae |
| ASV492 | TRUE | TRUE | Shared | *unknown* | Bacillaceae |
| ASV493 | TRUE | TRUE | Shared | *Akkermansia* | Akkermansiaceae |
| ASV5 | TRUE | TRUE | Shared | *Lysinibacillus* | Planococcaceae |
| ASV503 | TRUE | TRUE | Shared | *Atopostipes* | Carnobacteriaceae |
| ASV504 | TRUE | TRUE | Shared | *Atopostipes* | Carnobacteriaceae |
| ASV506 | TRUE | TRUE | Shared | *unknown* | Cellvibrionaceae |
| ASV507 | TRUE | TRUE | Shared | *Tessaracoccus* | Propionibacteriaceae |
| ASV51 | TRUE | TRUE | Shared | *Georgenia* | Bogoriellaceae |
| ASV511 | TRUE | TRUE | Shared | *Bacteroides* | Bacteroidaceae |
| ASV512 | TRUE | TRUE | Shared | *unknown* | Demequinaceae |
| ASV517 | TRUE | TRUE | Shared | *Amphibacillus* | Bacillaceae |
| ASV52 | TRUE | TRUE | Shared | *unknown* | Peptostreptococcaceae |
| ASV525 | TRUE | TRUE | Shared | *UCG-005* | Oscillospiraceae |
| ASV528 | TRUE | TRUE | Shared | *Atopostipes* | Carnobacteriaceae |
| ASV529 | TRUE | TRUE | Shared | *UCG-005* | Oscillospiraceae |
| ASV53 | TRUE | TRUE | Shared | *Ornithinimicrobium* | Intrasporangiaceae |
| ASV530 | TRUE | TRUE | Shared | *unknown* | Ruminococcaceae |
| ASV532 | TRUE | TRUE | Shared | *Flavobacterium* | Flavobacteriaceae |
| ASV534 | TRUE | TRUE | Shared | *Cellvibrio* | Cellvibrionaceae |
| ASV545 | TRUE | TRUE | Shared | *Fibrobacter* | Fibrobacteraceae |
| ASV547 | TRUE | TRUE | Shared | *unknown* | Bacillaceae |
| ASV55 | TRUE | TRUE | Shared | *unknown* | Micrococcaceae |
| ASV553 | TRUE | TRUE | Shared | *unknown* | unknown |
| ASV56 | TRUE | TRUE | Shared | *Alkalibacterium* | Carnobacteriaceae |
| ASV57 | TRUE | TRUE | Shared | *Georgenia* | Bogoriellaceae |
| ASV576 | TRUE | TRUE | Shared | *Pseudohongiella* | Pseudohongiellaceae |
| ASV5784 | TRUE | TRUE | Shared | *unknown* | Rhodobacteraceae |
| ASV58 | TRUE | TRUE | Shared | *unknown* | Planococcaceae |
| ASV587 | TRUE | TRUE | Shared | *Lysinibacillus* | Planococcaceae |
| ASV589 | TRUE | TRUE | Shared | *Candidatus Saccharimonas* | Saccharimonadaceae |
| ASV59 | TRUE | TRUE | Shared | *Nocardiopsis* | Nocardiopsaceae |
| ASV590 | TRUE | TRUE | Shared | *Alistipes* | Rikenellaceae |
| ASV594 | TRUE | TRUE | Shared | *unknown* | JG30-KF-CM45 |
| ASV598 | TRUE | TRUE | Shared | *Lachnospiraceae UCG-010* | Lachnospiraceae |
| ASV600 | TRUE | TRUE | Shared | *Coprococcus* | Lachnospiraceae |
| ASV602 | TRUE | TRUE | Shared | *unknown* | Flavobacteriaceae |
| ASV61 | TRUE | TRUE | Shared | *Salinicoccus* | Staphylococcaceae |
| ASV615 | TRUE | TRUE | Shared | *Georgenia* | Bogoriellaceae |
| ASV62 | TRUE | TRUE | Shared | *Aliicoccus* | Staphylococcaceae |
| ASV621 | TRUE | TRUE | Shared | *Sporosarcina* | Planococcaceae |
| ASV624 | TRUE | TRUE | Shared | *UCG-005* | Oscillospiraceae |
| ASV63 | TRUE | TRUE | Shared | *Pseudomonas* | Pseudomonadaceae |
| ASV636 | TRUE | TRUE | Shared | *unknown* | unknown |
| ASV638 | TRUE | TRUE | Shared | *unknown* | Bacillaceae |
| ASV64 | TRUE | TRUE | Shared | *Planococcus* | Planococcaceae |
| ASV642 | TRUE | TRUE | Shared | *unknown* | Bacillaceae |
| ASV644 | TRUE | TRUE | Shared | *Ornithinimicrobium* | Intrasporangiaceae |
| ASV645 | TRUE | TRUE | Shared | *unknown* | unknown |
| ASV65 | TRUE | TRUE | Shared | *Atopostipes* | Carnobacteriaceae |
| ASV652 | TRUE | TRUE | Shared | *C1-B045* | Porticoccaceae |
| ASV655 | TRUE | TRUE | Shared | *Brevundimonas* | Caulobacteraceae |
| ASV659 | TRUE | TRUE | Shared | *unknown* | Nitriliruptoraceae |
| ASV66 | TRUE | TRUE | Shared | *Salinicoccus* | Staphylococcaceae |
| ASV661 | TRUE | TRUE | Shared | *[Eubacterium] nodatum group* | Anaerovoracaceae |
| ASV668 | TRUE | TRUE | Shared | *unknown* | Longimicrobiaceae |
| ASV669 | TRUE | TRUE | Shared | *Luteococcus* | Propionibacteriaceae |
| ASV67 | TRUE | TRUE | Shared | *unknown* | Bacillaceae |
| ASV672 | TRUE | TRUE | Shared | *Flaviflexus* | Actinomycetaceae |
| ASV674 | TRUE | TRUE | Shared | *Stenotrophomonas* | Xanthomonadaceae |
| ASV68 | TRUE | TRUE | Shared | *unknown* | Planococcaceae |
| ASV682 | TRUE | TRUE | Shared | *Frisingicoccus* | Lachnospiraceae |
| ASV686 | TRUE | TRUE | Shared | *Nocardioides* | Nocardioidaceae |
| ASV691 | TRUE | TRUE | Shared | *unknown* | Microbacteriaceae |
| ASV695 | TRUE | TRUE | Shared | *UCG-005* | Oscillospiraceae |
| ASV7 | TRUE | TRUE | Shared | *Corynebacterium* | Corynebacteriaceae |
| ASV71 | TRUE | TRUE | Shared | *Oceanobacillus* | Bacillaceae |
| ASV718 | TRUE | TRUE | Shared | *Garicola* | Micrococcaceae |
| ASV719 | TRUE | TRUE | Shared | *Alistipes* | Rikenellaceae |
| ASV72 | TRUE | TRUE | Shared | *Corynebacterium* | Corynebacteriaceae |
| ASV724 | TRUE | TRUE | Shared | *unknown* | unknown |
| ASV73 | TRUE | TRUE | Shared | *Aliidiomarina* | Idiomarinaceae |
| ASV736 | TRUE | TRUE | Shared | *unknown* | Bacillaceae |
| ASV74 | TRUE | TRUE | Shared | *Brachybacterium* | Dermabacteraceae |
| ASV742 | TRUE | TRUE | Shared | *Corynebacterium* | Corynebacteriaceae |
| ASV747 | TRUE | TRUE | Shared | *Alkalibacterium* | Carnobacteriaceae |
| ASV75 | TRUE | TRUE | Shared | *Lysobacter* | Xanthomonadaceae |
| ASV760 | TRUE | TRUE | Shared | *Ornithinimicrobium* | Intrasporangiaceae |
| ASV764 | TRUE | TRUE | Shared | *unknown* | Bacillaceae |
| ASV77 | TRUE | TRUE | Shared | *Salinicoccus* | Staphylococcaceae |
| ASV770 | TRUE | TRUE | Shared | *Phascolarctobacterium* | Acidaminococcaceae |
| ASV777 | TRUE | TRUE | Shared | *Brevibacterium* | Brevibacteriaceae |
| ASV78 | TRUE | TRUE | Shared | *Pseudomonas* | Pseudomonadaceae |
| ASV781 | TRUE | TRUE | Shared | *Mailhella* | Desulfovibrionaceae |
| ASV789 | TRUE | TRUE | Shared | *Oceanobacillus* | Bacillaceae |
| ASV79 | TRUE | TRUE | Shared | *unknown* | Bacillaceae |
| ASV795 | TRUE | TRUE | Shared | *Verticiella* | Alcaligenaceae |
| ASV796 | TRUE | TRUE | Shared | *unknown* | Balneolaceae |
| ASV8 | TRUE | TRUE | Shared | *Romboutsia* | Peptostreptococcaceae |
| ASV81 | TRUE | TRUE | Shared | *Salinicoccus* | Staphylococcaceae |
| ASV818 | TRUE | TRUE | Shared | *Dietzia* | Dietziaceae |
| ASV819 | TRUE | TRUE | Shared | *Ornithinimicrobium* | Intrasporangiaceae |
| ASV83 | TRUE | TRUE | Shared | *UCG-005* | Oscillospiraceae |
| ASV834 | TRUE | TRUE | Shared | *Acinetobacter* | Moraxellaceae |
| ASV842 | TRUE | TRUE | Shared | *Halomonas* | Halomonadaceae |
| ASV849 | TRUE | TRUE | Shared | *Bacteroides* | Bacteroidaceae |
| ASV85 | TRUE | TRUE | Shared | *Corynebacterium* | Corynebacteriaceae |
| ASV853 | TRUE | TRUE | Shared | *Irregularibacter* | Garciellaceae |
| ASV855 | TRUE | TRUE | Shared | *unknown* | unknown |
| ASV86 | TRUE | TRUE | Shared | *UCG-005* | Oscillospiraceae |
| ASV863 | TRUE | TRUE | Shared | *Taibaiella* | Chitinophagaceae |
| ASV867 | TRUE | TRUE | Shared | *Sphingobacterium* | Sphingobacteriaceae |
| ASV87 | TRUE | TRUE | Shared | *unknown* | Cellvibrionaceae |
| ASV874 | TRUE | TRUE | Shared | *unknown* | unknown |
| ASV876 | TRUE | TRUE | Shared | *unknown* | Cellvibrionaceae |
| ASV88 | TRUE | TRUE | Shared | *Corynebacterium* | Corynebacteriaceae |
| ASV881 | TRUE | TRUE | Shared | *unknown* | unknown |
| ASV886 | TRUE | TRUE | Shared | *Halomonas* | Halomonadaceae |
| ASV895 | TRUE | TRUE | Shared | *Antricoccus* | Geodermatophilaceae |
| ASV9 | TRUE | TRUE | Shared | *Dietzia* | Dietziaceae |
| ASV90 | TRUE | TRUE | Shared | *Solibacillus* | Planococcaceae |
| ASV91 | TRUE | TRUE | Shared | *unknown* | Planococcaceae |
| ASV92 | TRUE | TRUE | Shared | *Pseudomonas* | Pseudomonadaceae |
| ASV93 | TRUE | TRUE | Shared | *Aliicoccus* | Staphylococcaceae |
| ASV94 | TRUE | TRUE | Shared | *Enteractinococcus* | Micrococcaceae |
| ASV940 | TRUE | TRUE | Shared | *Paenalcaligenes* | Alcaligenaceae |
| ASV958 | TRUE | TRUE | Shared | *Amphibacillus* | Bacillaceae |
| ASV97 | TRUE | TRUE | Shared | *Pseudomonas* | Pseudomonadaceae |
| ASV970 | TRUE | TRUE | Shared | *unknown* | Nitriliruptoraceae |
| ASV976 | TRUE | TRUE | Shared | *Acetitomaculum* | Lachnospiraceae |
| ASV98 | TRUE | TRUE | Shared | *Alkalibacterium* | Carnobacteriaceae |
| ASV984 | TRUE | TRUE | Shared | *Bacteroides* | Bacteroidaceae |
| ASV991 | TRUE | TRUE | Shared | *Nesterenkonia* | Micrococcaceae |
| ASV999 | TRUE | TRUE | Shared | *unknown* | p-251-o5 |
| ASV1008 | TRUE | FALSE | Only dry | *unknown* | [Eubacterium] coprostanoligenes group |
| ASV1016 | TRUE | FALSE | Only dry | *unknown* | unknown |
| ASV1019 | TRUE | FALSE | Only dry | *Oceanobacillus* | Bacillaceae |
| ASV1026 | TRUE | FALSE | Only dry | *unknown* | Bacillaceae |
| ASV1029 | TRUE | FALSE | Only dry | *Marinimicrobium* | Cellvibrionaceae |
| ASV1030 | TRUE | FALSE | Only dry | *unknown* | UCG-010 |
| ASV1034 | TRUE | FALSE | Only dry | *Amphibacillus* | Bacillaceae |
| ASV1040 | TRUE | FALSE | Only dry | *Pseudactinotalea* | Cellulomonadaceae |
| ASV1046 | TRUE | FALSE | Only dry | *Wenzhouxiangella* | Unknown Family |
| ASV1050 | TRUE | FALSE | Only dry | *unknown* | Bacillaceae |
| ASV1053 | TRUE | FALSE | Only dry | *Dietzia* | Dietziaceae |
| ASV1056 | TRUE | FALSE | Only dry | *Amphibacillus* | Bacillaceae |
| ASV1060 | TRUE | FALSE | Only dry | *Acetitomaculum* | Lachnospiraceae |
| ASV1063 | TRUE | FALSE | Only dry | *unknown* | UCG-010 |
| ASV107 | TRUE | FALSE | Only dry | *Bifidobacterium* | Bifidobacteriaceae |
| ASV1072 | TRUE | FALSE | Only dry | *UCG-005* | Oscillospiraceae |
| ASV1080 | TRUE | FALSE | Only dry | *Amphibacillus* | Bacillaceae |
| ASV1095 | TRUE | FALSE | Only dry | *Acholeplasma* | Acholeplasmataceae |
| ASV10951 | TRUE | FALSE | Only dry | *Lachnospiraceae FCS020 group* | Lachnospiraceae |
| ASV10960 | TRUE | FALSE | Only dry | *unknown* | unknown |
| ASV10965 | TRUE | FALSE | Only dry | *Alistipes* | Rikenellaceae |
| ASV1101 | TRUE | FALSE | Only dry | *Rikenellaceae RC9 gut group* | Rikenellaceae |
| ASV11047 | TRUE | FALSE | Only dry | *unknown* | UCG-010 |
| ASV1110 | TRUE | FALSE | Only dry | *Alistipes* | Rikenellaceae |
| ASV1118 | TRUE | FALSE | Only dry | *Methanobrevibacter* | Methanobacteriaceae |
| ASV11199 | TRUE | FALSE | Only dry | *Facklamia* | Aerococcaceae |
| ASV1120 | TRUE | FALSE | Only dry | *Cellvibrio* | Cellvibrionaceae |
| ASV1133 | TRUE | FALSE | Only dry | *Comamonas* | Comamonadaceae |
| ASV1139 | TRUE | FALSE | Only dry | *Corynebacterium* | Corynebacteriaceae |
| ASV1140 | TRUE | FALSE | Only dry | *Alistipes* | Rikenellaceae |
| ASV1143 | TRUE | FALSE | Only dry | *unknown* | UCG-010 |
| ASV1151 | TRUE | FALSE | Only dry | *Alloprevotella* | Prevotellaceae |
| ASV1153 | TRUE | FALSE | Only dry | *unknown* | unknown |
| ASV1160 | TRUE | FALSE | Only dry | *Halomonas* | Halomonadaceae |
| ASV1162 | TRUE | FALSE | Only dry | *unknown* | unknown |
| ASV11621 | TRUE | FALSE | Only dry | *Anaerovorax* | Anaerovoracaceae |
| ASV1166 | TRUE | FALSE | Only dry | *UCG-005* | Oscillospiraceae |
| ASV11694 | TRUE | FALSE | Only dry | *unknown* | Ruminococcaceae |
| ASV11703 | TRUE | FALSE | Only dry | *unknown* | Bacillaceae |
| ASV1172 | TRUE | FALSE | Only dry | *unknown* | unknown |
| ASV1175 | TRUE | FALSE | Only dry | *Bacteroides* | Bacteroidaceae |
| ASV1177 | TRUE | FALSE | Only dry | *Garicola* | Micrococcaceae |
| ASV1178 | TRUE | FALSE | Only dry | *unknown* | unknown |
| ASV1179 | TRUE | FALSE | Only dry | *TM7a* | Saccharimonadaceae |
| ASV1183 | TRUE | FALSE | Only dry | *Jeotgalibaca* | Carnobacteriaceae |
| ASV1185 | TRUE | FALSE | Only dry | *Idiomarina* | Idiomarinaceae |
| ASV11871 | TRUE | FALSE | Only dry | *unknown* | unknown |
| ASV1193 | TRUE | FALSE | Only dry | *NK4A214 group* | Oscillospiraceae |
| ASV11936 | TRUE | FALSE | Only dry | *unknown* | Paenibacillaceae |
| ASV1194 | TRUE | FALSE | Only dry | *TM7a* | Saccharimonadaceae |
| ASV1196 | TRUE | FALSE | Only dry | *Cellulosilyticum* | Lachnospiraceae |
| ASV11968 | TRUE | FALSE | Only dry | *Christensenellaceae R-7 group* | Christensenellaceae |
| ASV11984 | TRUE | FALSE | Only dry | *unknown* | unknown |
| ASV1199 | TRUE | FALSE | Only dry | *Prevotellaceae Ga6A1 group* | Prevotellaceae |
| ASV1204 | TRUE | FALSE | Only dry | *Ruminococcus* | Ruminococcaceae |
| ASV1208 | TRUE | FALSE | Only dry | *unknown* | unknown |
| ASV1224 | TRUE | FALSE | Only dry | *Ornithinibacillus* | Bacillaceae |
| ASV1225 | TRUE | FALSE | Only dry | *UCG-005* | Oscillospiraceae |
| ASV12261 | TRUE | FALSE | Only dry | *unknown* | unknown |
| ASV1229 | TRUE | FALSE | Only dry | *unknown* | Oscillospiraceae |
| ASV12309 | TRUE | FALSE | Only dry | *NK4A214 group* | Oscillospiraceae |
| ASV1234 | TRUE | FALSE | Only dry | *Rikenellaceae RC9 gut group* | Rikenellaceae |
| ASV1244 | TRUE | FALSE | Only dry | *unknown* | Bacillaceae |
| ASV1246 | TRUE | FALSE | Only dry | *unknown* | Ruminococcaceae |
| ASV1250 | TRUE | FALSE | Only dry | *unknown* | unknown |
| ASV1256 | TRUE | FALSE | Only dry | *Prevotellaceae UCG-003* | Prevotellaceae |
| ASV1259 | TRUE | FALSE | Only dry | *Acinetobacter* | Moraxellaceae |
| ASV12594 | TRUE | FALSE | Only dry | *unknown* | UCG-010 |
| ASV12598 | TRUE | FALSE | Only dry | *Alistipes* | Rikenellaceae |
| ASV1260 | TRUE | FALSE | Only dry | *Lysinibacillus* | Planococcaceae |
| ASV12616 | TRUE | FALSE | Only dry | *Ignatzschineria* | Wohlfahrtiimonadaceae |
| ASV12628 | TRUE | FALSE | Only dry | *unknown* | unknown |
| ASV1273 | TRUE | FALSE | Only dry | *unknown* | Guggenheimella |
| ASV1275 | TRUE | FALSE | Only dry | *Rikenellaceae RC9 gut group* | Rikenellaceae |
| ASV1285 | TRUE | FALSE | Only dry | *Prevotellaceae UCG-003* | Prevotellaceae |
| ASV12860 | TRUE | FALSE | Only dry | *Aliicoccus* | Staphylococcaceae |
| ASV1291 | TRUE | FALSE | Only dry | *Bacteroides* | Bacteroidaceae |
| ASV1303 | TRUE | FALSE | Only dry | *Oceanobacillus* | Bacillaceae |
| ASV1305 | TRUE | FALSE | Only dry | *Vibrio* | Vibrionaceae |
| ASV1306 | TRUE | FALSE | Only dry | *Prevotellaceae UCG-003* | Prevotellaceae |
| ASV1308 | TRUE | FALSE | Only dry | *Aliicoccus* | Staphylococcaceae |
| ASV1311 | TRUE | FALSE | Only dry | *Solibacillus* | Planococcaceae |
| ASV1313 | TRUE | FALSE | Only dry | *UCG-005* | Oscillospiraceae |
| ASV1317 | TRUE | FALSE | Only dry | *Prevotella* | Prevotellaceae |
| ASV1318 | TRUE | FALSE | Only dry | *Prevotellaceae UCG-003* | Prevotellaceae |
| ASV1320 | TRUE | FALSE | Only dry | *unknown* | [Eubacterium] coprostanoligenes group |
| ASV13203 | TRUE | FALSE | Only dry | *Dietzia* | Dietziaceae |
| ASV1321 | TRUE | FALSE | Only dry | *unknown* | Vibrionaceae |
| ASV1328 | TRUE | FALSE | Only dry | *Succinivibrio* | Succinivibrionaceae |
| ASV1330 | TRUE | FALSE | Only dry | *unknown* | UCG-010 |
| ASV1344 | TRUE | FALSE | Only dry | *Rikenellaceae RC9 gut group* | Rikenellaceae |
| ASV1351 | TRUE | FALSE | Only dry | *Prevotellaceae UCG-003* | Prevotellaceae |
| ASV13537 | TRUE | FALSE | Only dry | *Alistipes* | Rikenellaceae |
| ASV1354 | TRUE | FALSE | Only dry | *Rikenellaceae RC9 gut group* | Rikenellaceae |
| ASV13544 | TRUE | FALSE | Only dry | *unknown* | Desulfocapsaceae |
| ASV1355 | TRUE | FALSE | Only dry | *Acholeplasma* | Acholeplasmataceae |
| ASV1357 | TRUE | FALSE | Only dry | *Oscillibacter* | Oscillospiraceae |
| ASV13595 | TRUE | FALSE | Only dry | *Family XIII AD3011 group* | Anaerovoracaceae |
| ASV13613 | TRUE | FALSE | Only dry | *unknown* | unknown |
| ASV1363 | TRUE | FALSE | Only dry | *unknown* | Lachnospiraceae |
| ASV1369 | TRUE | FALSE | Only dry | *Atopostipes* | Carnobacteriaceae |
| ASV1370 | TRUE | FALSE | Only dry | *unknown* | UCG-010 |
| ASV1385 | TRUE | FALSE | Only dry | *Pygmaiobacter* | Ruminococcaceae |
| ASV1396 | TRUE | FALSE | Only dry | *unknown* | Paludibacteraceae |
| ASV1399 | TRUE | FALSE | Only dry | *Acinetobacter* | Moraxellaceae |
| ASV140 | TRUE | FALSE | Only dry | *unknown* | Bacillaceae |
| ASV1400 | TRUE | FALSE | Only dry | *Haloplasma* | Haloplasmataceae |
| ASV1409 | TRUE | FALSE | Only dry | *unknown* | F082 |
| ASV1411 | TRUE | FALSE | Only dry | *Alistipes* | Rikenellaceae |
| ASV1415 | TRUE | FALSE | Only dry | *unknown* | Lachnospiraceae |
| ASV14159 | TRUE | FALSE | Only dry | *unknown* | UCG-010 |
| ASV14215 | TRUE | FALSE | Only dry | *Odoribacter* | Marinifilaceae |
| ASV14237 | TRUE | FALSE | Only dry | *unknown* | Corynebacteriaceae |
| ASV14238 | TRUE | FALSE | Only dry | *unknown* | unknown |
| ASV1426 | TRUE | FALSE | Only dry | *unknown* | UCG-010 |
| ASV1427 | TRUE | FALSE | Only dry | *unknown* | unknown |
| ASV1428 | TRUE | FALSE | Only dry | *unknown* | Alkaliphilus |
| ASV1438 | TRUE | FALSE | Only dry | *unknown* | Lachnospiraceae |
| ASV1441 | TRUE | FALSE | Only dry | *Prevotellaceae UCG-004* | Prevotellaceae |
| ASV1450 | TRUE | FALSE | Only dry | *unknown* | [Eubacterium] coprostanoligenes group |
| ASV1457 | TRUE | FALSE | Only dry | *Rikenellaceae RC9 gut group* | Rikenellaceae |
| ASV146 | TRUE | FALSE | Only dry | *Bacillus* | Bacillaceae |
| ASV1463 | TRUE | FALSE | Only dry | *Providencia* | Morganellaceae |
| ASV14637 | TRUE | FALSE | Only dry | *unknown* | Oscillospiraceae |
| ASV1472 | TRUE | FALSE | Only dry | *Ignatzschineria* | Wohlfahrtiimonadaceae |
| ASV14722 | TRUE | FALSE | Only dry | *Yaniella* | Micrococcaceae |
| ASV1473 | TRUE | FALSE | Only dry | *Alloprevotella* | Prevotellaceae |
| ASV1474 | TRUE | FALSE | Only dry | *NK4A214 group* | Oscillospiraceae |
| ASV1483 | TRUE | FALSE | Only dry | *unknown* | Bacillaceae |
| ASV1491 | TRUE | FALSE | Only dry | *Bacteroides* | Bacteroidaceae |
| ASV1492 | TRUE | FALSE | Only dry | *Dietzia* | Dietziaceae |
| ASV1495 | TRUE | FALSE | Only dry | *unknown* | Bacillaceae |
| ASV1502 | TRUE | FALSE | Only dry | *unknown* | Rhodobacteraceae |
| ASV15030 | TRUE | FALSE | Only dry | *unknown* | unknown |
| ASV15063 | TRUE | FALSE | Only dry | *unknown* | Bradymonadaceae |
| ASV1507 | TRUE | FALSE | Only dry | *unknown* | UCG-010 |
| ASV15070 | TRUE | FALSE | Only dry | *Aliicoccus* | Staphylococcaceae |
| ASV1508 | TRUE | FALSE | Only dry | *Gracilibacillus* | Bacillaceae |
| ASV15080 | TRUE | FALSE | Only dry | *unknown* | unknown |
| ASV1510 | TRUE | FALSE | Only dry | *Bacteroides* | Bacteroidaceae |
| ASV15163 | TRUE | FALSE | Only dry | *unknown* | p-2534-18B5 gut group |
| ASV1538 | TRUE | FALSE | Only dry | *Bacteroides* | Bacteroidaceae |
| ASV15468 | TRUE | FALSE | Only dry | *unknown* | unknown |
| ASV15491 | TRUE | FALSE | Only dry | *unknown* | Staphylococcaceae |
| ASV15542 | TRUE | FALSE | Only dry | *Atopostipes* | Carnobacteriaceae |
| ASV15546 | TRUE | FALSE | Only dry | *unknown* | p-251-o5 |
| ASV1557 | TRUE | FALSE | Only dry | *Bacteroides* | Bacteroidaceae |
| ASV15680 | TRUE | FALSE | Only dry | *unknown* | unknown |
| ASV15695 | TRUE | FALSE | Only dry | *Salinicoccus* | Staphylococcaceae |
| ASV1573 | TRUE | FALSE | Only dry | *unknown* | Oscillospiraceae |
| ASV1577 | TRUE | FALSE | Only dry | *Christensenellaceae R-7 group* | Christensenellaceae |
| ASV1585 | TRUE | FALSE | Only dry | *Bacteroides* | Bacteroidaceae |
| ASV1586 | TRUE | FALSE | Only dry | *Gulosibacter* | Microbacteriaceae |
| ASV1587 | TRUE | FALSE | Only dry | *unknown* | Sphingobacteriaceae |
| ASV1594 | TRUE | FALSE | Only dry | *Cellvibrio* | Cellvibrionaceae |
| ASV1606 | TRUE | FALSE | Only dry | *unknown* | Lachnospiraceae |
| ASV1614 | TRUE | FALSE | Only dry | *unknown* | UCG-010 |
| ASV162 | TRUE | FALSE | Only dry | *unknown* | Bacillaceae |
| ASV1628 | TRUE | FALSE | Only dry | *unknown* | UCG-010 |
| ASV1630 | TRUE | FALSE | Only dry | *Blautia* | Lachnospiraceae |
| ASV1631 | TRUE | FALSE | Only dry | *Prevotellaceae UCG-004* | Prevotellaceae |
| ASV1648 | TRUE | FALSE | Only dry | *GWE2-31-10* | Spirochaetaceae |
| ASV1649 | TRUE | FALSE | Only dry | *unknown* | UCG-010 |
| ASV16611 | TRUE | FALSE | Only dry | *unknown* | Lachnospiraceae |
| ASV1663 | TRUE | FALSE | Only dry | *unknown* | unknown |
| ASV1668 | TRUE | FALSE | Only dry | *unknown* | UCG-010 |
| ASV1669 | TRUE | FALSE | Only dry | *unknown* | UCG-010 |
| ASV1680 | TRUE | FALSE | Only dry | *unknown* | unknown |
| ASV1681 | TRUE | FALSE | Only dry | *Faecalitalea* | Erysipelotrichaceae |
| ASV1686 | TRUE | FALSE | Only dry | *Bacteroides* | Bacteroidaceae |
| ASV1691 | TRUE | FALSE | Only dry | *Rikenellaceae RC9 gut group* | Rikenellaceae |
| ASV16914 | TRUE | FALSE | Only dry | *Bacteroides* | Bacteroidaceae |
| ASV16931 | TRUE | FALSE | Only dry | *unknown* | unknown |
| ASV16936 | TRUE | FALSE | Only dry | *Rikenellaceae RC9 gut group* | Rikenellaceae |
| ASV1702 | TRUE | FALSE | Only dry | *Victivallis* | Victivallaceae |
| ASV1703 | TRUE | FALSE | Only dry | *Rikenellaceae RC9 gut group* | Rikenellaceae |
| ASV1713 | TRUE | FALSE | Only dry | *unknown* | Nitriliruptoraceae |
| ASV1714 | TRUE | FALSE | Only dry | *Bacteroides* | Bacteroidaceae |
| ASV1717 | TRUE | FALSE | Only dry | *Candidatus Stoquefichus* | Erysipelatoclostridiaceae |
| ASV1729 | TRUE | FALSE | Only dry | *unknown* | Cellvibrionaceae |
| ASV1738 | TRUE | FALSE | Only dry | *Bacteroides* | Bacteroidaceae |
| ASV1746 | TRUE | FALSE | Only dry | *Ornithinibacillus* | Bacillaceae |
| ASV1755 | TRUE | FALSE | Only dry | *unknown* | Muribaculaceae |
| ASV1765 | TRUE | FALSE | Only dry | *Ruminiclostridium* | Hungateiclostridiaceae |
| ASV1776 | TRUE | FALSE | Only dry | *unknown* | UCG-010 |
| ASV1778 | TRUE | FALSE | Only dry | *Aequorivita* | Flavobacteriaceae |
| ASV1780 | TRUE | FALSE | Only dry | *Oceanobacillus* | Bacillaceae |
| ASV1785 | TRUE | FALSE | Only dry | *Verticiella* | Alcaligenaceae |
| ASV17884 | TRUE | FALSE | Only dry | *unknown* | unknown |
| ASV1795 | TRUE | FALSE | Only dry | *unknown* | Bacillaceae |
| ASV1797 | TRUE | FALSE | Only dry | *Erysipelatoclostridium* | Erysipelatoclostridiaceae |
| ASV1802 | TRUE | FALSE | Only dry | *Pusillimonas* | Alcaligenaceae |
| ASV18105 | TRUE | FALSE | Only dry | *unknown* | unknown |
| ASV18120 | TRUE | FALSE | Only dry | *Aliicoccus* | Staphylococcaceae |
| ASV1813 | TRUE | FALSE | Only dry | *Rikenellaceae RC9 gut group* | Rikenellaceae |
| ASV1815 | TRUE | FALSE | Only dry | *unknown* | Carnobacteriaceae |
| ASV1828 | TRUE | FALSE | Only dry | *Rikenellaceae RC9 gut group* | Rikenellaceae |
| ASV1838 | TRUE | FALSE | Only dry | *Akkermansia* | Akkermansiaceae |
| ASV1850 | TRUE | FALSE | Only dry | *Bacillus* | Bacillaceae |
| ASV1862 | TRUE | FALSE | Only dry | *Prevotellaceae UCG-004* | Prevotellaceae |
| ASV1868 | TRUE | FALSE | Only dry | *Aerococcus* | Aerococcaceae |
| ASV18974 | TRUE | FALSE | Only dry | *Yaniella* | Micrococcaceae |
| ASV1903 | TRUE | FALSE | Only dry | *unknown* | Prevotellaceae |
| ASV1908 | TRUE | FALSE | Only dry | *unknown* | unknown |
| ASV1915 | TRUE | FALSE | Only dry | *Family XIII AD3011 group* | Anaerovoracaceae |
| ASV19303 | TRUE | FALSE | Only dry | *unknown* | unknown |
| ASV19403 | TRUE | FALSE | Only dry | *unknown* | unknown |
| ASV1941 | TRUE | FALSE | Only dry | *Oceanobacillus* | Bacillaceae |
| ASV1944 | TRUE | FALSE | Only dry | *Treponema* | Spirochaetaceae |
| ASV1956 | TRUE | FALSE | Only dry | *Alistipes* | Rikenellaceae |
| ASV1957 | TRUE | FALSE | Only dry | *unknown* | Bacillaceae |
| ASV1973 | TRUE | FALSE | Only dry | *Anaerovibrio* | Selenomonadaceae |
| ASV1986 | TRUE | FALSE | Only dry | *p-1088-a5 gut group* | Pirellulaceae |
| ASV19987 | TRUE | FALSE | Only dry | *unknown* | unknown |
| ASV2000 | TRUE | FALSE | Only dry | *unknown* | Bacillaceae |
| ASV20034 | TRUE | FALSE | Only dry | *unknown* | Planococcaceae |
| ASV2013 | TRUE | FALSE | Only dry | *unknown* | Oscillospiraceae |
| ASV2016 | TRUE | FALSE | Only dry | *Oceanobacillus* | Bacillaceae |
| ASV20183 | TRUE | FALSE | Only dry | *unknown* | unknown |
| ASV202 | TRUE | FALSE | Only dry | *unknown* | Planococcaceae |
| ASV20200 | TRUE | FALSE | Only dry | *Aliicoccus* | Staphylococcaceae |
| ASV20332 | TRUE | FALSE | Only dry | *unknown* | UCG-010 |
| ASV20352 | TRUE | FALSE | Only dry | *Aliidiomarina* | Idiomarinaceae |
| ASV2036 | TRUE | FALSE | Only dry | *Erysipelothrix* | Erysipelotrichaceae |
| ASV2045 | TRUE | FALSE | Only dry | *Prevotella* | Prevotellaceae |
| ASV2048 | TRUE | FALSE | Only dry | *Rikenellaceae RC9 gut group* | Rikenellaceae |
| ASV2056 | TRUE | FALSE | Only dry | *TM7a* | Saccharimonadaceae |
| ASV2073 | TRUE | FALSE | Only dry | *unknown* | unknown |
| ASV2086 | TRUE | FALSE | Only dry | *unknown* | [Eubacterium] coprostanoligenes group |
| ASV2087 | TRUE | FALSE | Only dry | *Pelagibacterium* | Devosiaceae |
| ASV2088 | TRUE | FALSE | Only dry | *unknown* | UCG-010 |
| ASV2097 | TRUE | FALSE | Only dry | *unknown* | Lachnospiraceae |
| ASV210 | TRUE | FALSE | Only dry | *Solibacillus* | Planococcaceae |
| ASV21018 | TRUE | FALSE | Only dry | *unknown* | Staphylococcaceae |
| ASV21155 | TRUE | FALSE | Only dry | *unknown* | Micrococcaceae |
| ASV2116 | TRUE | FALSE | Only dry | *Salinimicrobium* | Flavobacteriaceae |
| ASV2141 | TRUE | FALSE | Only dry | *Porphyromonas* | Porphyromonadaceae |
| ASV2143 | TRUE | FALSE | Only dry | *unknown* | unknown |
| ASV21512 | TRUE | FALSE | Only dry | *unknown* | unknown |
| ASV21516 | TRUE | FALSE | Only dry | *unknown* | unknown |
| ASV21537 | TRUE | FALSE | Only dry | *unknown* | unknown |
| ASV2162 | TRUE | FALSE | Only dry | *Prevotellaceae UCG-004* | Prevotellaceae |
| ASV2167 | TRUE | FALSE | Only dry | *unknown* | unknown |
| ASV217 | TRUE | FALSE | Only dry | *UCG-005* | Oscillospiraceae |
| ASV218 | TRUE | FALSE | Only dry | *unknown* | [Eubacterium] coprostanoligenes group |
| ASV2191 | TRUE | FALSE | Only dry | *Amphibacillus* | Bacillaceae |
| ASV2195 | TRUE | FALSE | Only dry | *Ulvibacter* | Flavobacteriaceae |
| ASV2198 | TRUE | FALSE | Only dry | *unknown* | Cyclobacteriaceae |
| ASV2199 | TRUE | FALSE | Only dry | *unknown* | UCG-010 |
| ASV220 | TRUE | FALSE | Only dry | *unknown* | Xanthomonadaceae |
| ASV2213 | TRUE | FALSE | Only dry | *unknown* | unknown |
| ASV2220 | TRUE | FALSE | Only dry | *unknown* | [Eubacterium] coprostanoligenes group |
| ASV2233 | TRUE | FALSE | Only dry | *unknown* | vadinBE97 |
| ASV2245 | TRUE | FALSE | Only dry | *unknown* | p-2534-18B5 gut group |
| ASV2254 | TRUE | FALSE | Only dry | *Acinetobacter* | Moraxellaceae |
| ASV2270 | TRUE | FALSE | Only dry | *C1-B045* | Porticoccaceae |
| ASV2273 | TRUE | FALSE | Only dry | *Alloprevotella* | Prevotellaceae |
| ASV2278 | TRUE | FALSE | Only dry | *Monoglobus* | Monoglobaceae |
| ASV2285 | TRUE | FALSE | Only dry | *Rikenellaceae RC9 gut group* | Rikenellaceae |
| ASV2291 | TRUE | FALSE | Only dry | *unknown* | UCG-010 |
| ASV22977 | TRUE | FALSE | Only dry | *UCG-005* | Oscillospiraceae |
| ASV23007 | TRUE | FALSE | Only dry | *[Eubacterium] tenue group* | Peptostreptococcaceae |
| ASV23019 | TRUE | FALSE | Only dry | *unknown* | Intrasporangiaceae |
| ASV23024 | TRUE | FALSE | Only dry | *Salinimicrobium* | Flavobacteriaceae |
| ASV23081 | TRUE | FALSE | Only dry | *unknown* | unknown |
| ASV23153 | TRUE | FALSE | Only dry | *unknown* | unknown |
| ASV23171 | TRUE | FALSE | Only dry | *unknown* | Xanthomonadaceae |
| ASV23172 | TRUE | FALSE | Only dry | *unknown* | Ruminococcaceae |
| ASV2321 | TRUE | FALSE | Only dry | *unknown* | UCG-010 |
| ASV23235 | TRUE | FALSE | Only dry | *unknown* | unknown |
| ASV2336 | TRUE | FALSE | Only dry | *unknown* | unknown |
| ASV23397 | TRUE | FALSE | Only dry | *unknown* | unknown |
| ASV2350 | TRUE | FALSE | Only dry | *Ornithobacterium* | Weeksellaceae |
| ASV2365 | TRUE | FALSE | Only dry | *Aliidiomarina* | Idiomarinaceae |
| ASV2376 | TRUE | FALSE | Only dry | *Rikenellaceae RC9 gut group* | Rikenellaceae |
| ASV2377 | TRUE | FALSE | Only dry | *Bacillus* | Bacillaceae |
| ASV2381 | TRUE | FALSE | Only dry | *Sphingobacterium* | Sphingobacteriaceae |
| ASV2387 | TRUE | FALSE | Only dry | *Christensenellaceae R-7 group* | Christensenellaceae |
| ASV2406 | TRUE | FALSE | Only dry | *Rikenellaceae RC9 gut group* | Rikenellaceae |
| ASV2434 | TRUE | FALSE | Only dry | *Prevotellaceae UCG-004* | Prevotellaceae |
| ASV2451 | TRUE | FALSE | Only dry | *unknown* | unknown |
| ASV246 | TRUE | FALSE | Only dry | *Corynebacterium* | Corynebacteriaceae |
| ASV2469 | TRUE | FALSE | Only dry | *unknown* | Oscillospiraceae |
| ASV249 | TRUE | FALSE | Only dry | *Akkermansia* | Akkermansiaceae |
| ASV2496 | TRUE | FALSE | Only dry | *Bacteroides* | Bacteroidaceae |
| ASV2507 | TRUE | FALSE | Only dry | *Incertae Sedis* | Ruminococcaceae |
| ASV2508 | TRUE | FALSE | Only dry | *unknown* | Lachnospiraceae |
| ASV2515 | TRUE | FALSE | Only dry | *unknown* | UCG-010 |
| ASV2530 | TRUE | FALSE | Only dry | *unknown* | [Eubacterium] coprostanoligenes group |
| ASV2543 | TRUE | FALSE | Only dry | *Rikenellaceae RC9 gut group* | Rikenellaceae |
| ASV255 | TRUE | FALSE | Only dry | *UCG-005* | Oscillospiraceae |
| ASV2557 | TRUE | FALSE | Only dry | *unknown* | unknown |
| ASV2565 | TRUE | FALSE | Only dry | *Pseudohongiella* | Pseudohongiellaceae |
| ASV2571 | TRUE | FALSE | Only dry | *Fastidiosipila* | Hungateiclostridiaceae |
| ASV2573 | TRUE | FALSE | Only dry | *Rikenellaceae RC9 gut group* | Rikenellaceae |
| ASV2585 | TRUE | FALSE | Only dry | *unknown* | Bacillaceae |
| ASV2603 | TRUE | FALSE | Only dry | *Rikenellaceae RC9 gut group* | Rikenellaceae |
| ASV2604 | TRUE | FALSE | Only dry | *UCG-005* | Oscillospiraceae |
| ASV26111 | TRUE | FALSE | Only dry | *unknown* | Staphylococcaceae |
| ASV26197 | TRUE | FALSE | Only dry | *Salinicoccus* | Staphylococcaceae |
| ASV26199 | TRUE | FALSE | Only dry | *unknown* | unknown |
| ASV2625 | TRUE | FALSE | Only dry | *Gordonia* | Nocardiaceae |
| ASV2626 | TRUE | FALSE | Only dry | *unknown* | UCG-010 |
| ASV26270 | TRUE | FALSE | Only dry | *Yaniella* | Micrococcaceae |
| ASV2628 | TRUE | FALSE | Only dry | *unknown* | Muribaculaceae |
| ASV2630 | TRUE | FALSE | Only dry | *unknown* | unknown |
| ASV26346 | TRUE | FALSE | Only dry | *unknown* | unknown |
| ASV26454 | TRUE | FALSE | Only dry | *Mailhella* | Desulfovibrionaceae |
| ASV2647 | TRUE | FALSE | Only dry | *Christensenellaceae R-7 group* | Christensenellaceae |
| ASV26555 | TRUE | FALSE | Only dry | *unknown* | unknown |
| ASV2658 | TRUE | FALSE | Only dry | *unknown* | Bacillaceae |
| ASV26583 | TRUE | FALSE | Only dry | *unknown* | Staphylococcaceae |
| ASV26613 | TRUE | FALSE | Only dry | *unknown* | unknown |
| ASV2672 | TRUE | FALSE | Only dry | *unknown* | UCG-010 |
| ASV2681 | TRUE | FALSE | Only dry | *unknown* | Lachnospiraceae |
| ASV2688 | TRUE | FALSE | Only dry | *unknown* | Bacteroidales UCG-001 |
| ASV2700 | TRUE | FALSE | Only dry | *Oceanobacillus* | Bacillaceae |
| ASV2713 | TRUE | FALSE | Only dry | *Rikenellaceae RC9 gut group* | Rikenellaceae |
| ASV272 | TRUE | FALSE | Only dry | *Altererythrobacter* | Sphingomonadaceae |
| ASV2746 | TRUE | FALSE | Only dry | *Candidatus Soleaferrea* | Ruminococcaceae |
| ASV2759 | TRUE | FALSE | Only dry | *unknown* | unknown |
| ASV2784 | TRUE | FALSE | Only dry | *Alloprevotella* | Prevotellaceae |
| ASV2800 | TRUE | FALSE | Only dry | *Erysipelothrix* | Erysipelotrichaceae |
| ASV2818 | TRUE | FALSE | Only dry | *Bacteroides* | Bacteroidaceae |
| ASV28302 | TRUE | FALSE | Only dry | *unknown* | UCG-010 |
| ASV284 | TRUE | FALSE | Only dry | *unknown* | Carnobacteriaceae |
| ASV2852 | TRUE | FALSE | Only dry | *Chelativorans* | Rhizobiaceae |
| ASV2853 | TRUE | FALSE | Only dry | *[Anaerorhabdus] furcosa group* | Erysipelotrichaceae |
| ASV28538 | TRUE | FALSE | Only dry | *NK4A214 group* | Oscillospiraceae |
| ASV2869 | TRUE | FALSE | Only dry | *Acholeplasma* | Acholeplasmataceae |
| ASV28711 | TRUE | FALSE | Only dry | *unknown* | unknown |
| ASV2876 | TRUE | FALSE | Only dry | *unknown* | Muribaculaceae |
| ASV28837 | TRUE | FALSE | Only dry | *unknown* | unknown |
| ASV28842 | TRUE | FALSE | Only dry | *unknown* | unknown |
| ASV2887 | TRUE | FALSE | Only dry | *unknown* | unknown |
| ASV28941 | TRUE | FALSE | Only dry | *unknown* | Idiomarinaceae |
| ASV28967 | TRUE | FALSE | Only dry | *Acholeplasma* | Acholeplasmataceae |
| ASV28968 | TRUE | FALSE | Only dry | *UCG-005* | Oscillospiraceae |
| ASV29047 | TRUE | FALSE | Only dry | *unknown* | unknown |
| ASV29149 | TRUE | FALSE | Only dry | *unknown* | Staphylococcaceae |
| ASV29173 | TRUE | FALSE | Only dry | *unknown* | unknown |
| ASV2919 | TRUE | FALSE | Only dry | *unknown* | UCG-010 |
| ASV2933 | TRUE | FALSE | Only dry | *unknown* | F082 |
| ASV2941 | TRUE | FALSE | Only dry | *unknown* | Muribaculaceae |
| ASV2943 | TRUE | FALSE | Only dry | *unknown* | unknown |
| ASV2954 | TRUE | FALSE | Only dry | *Lachnospiraceae AC2044 group* | Lachnospiraceae |
| ASV2961 | TRUE | FALSE | Only dry | *Christensenellaceae R-7 group* | Christensenellaceae |
| ASV2978 | TRUE | FALSE | Only dry | *Frisingicoccus* | Lachnospiraceae |
| ASV2997 | TRUE | FALSE | Only dry | *unknown* | Lachnospiraceae |
| ASV3021 | TRUE | FALSE | Only dry | *unknown* | unknown |
| ASV30368 | TRUE | FALSE | Only dry | *Yaniella* | Micrococcaceae |
| ASV3037 | TRUE | FALSE | Only dry | *Ignavigranum* | Aerococcaceae |
| ASV3041 | TRUE | FALSE | Only dry | *Alistipes* | Rikenellaceae |
| ASV3048 | TRUE | FALSE | Only dry | *Christensenellaceae R-7 group* | Christensenellaceae |
| ASV30758 | TRUE | FALSE | Only dry | *Salinicoccus* | Staphylococcaceae |
| ASV3077 | TRUE | FALSE | Only dry | *Luteimonas* | Xanthomonadaceae |
| ASV3079 | TRUE | FALSE | Only dry | *unknown* | UCG-010 |
| ASV30949 | TRUE | FALSE | Only dry | *unknown* | unknown |
| ASV30990 | TRUE | FALSE | Only dry | *unknown* | UCG-010 |
| ASV3104 | TRUE | FALSE | Only dry | *unknown* | unknown |
| ASV3111 | TRUE | FALSE | Only dry | *Rikenellaceae RC9 gut group* | Rikenellaceae |
| ASV313 | TRUE | FALSE | Only dry | *Truepera* | Trueperaceae |
| ASV3220 | TRUE | FALSE | Only dry | *unknown* | Balneolaceae |
| ASV324 | TRUE | FALSE | Only dry | *UCG-005* | Oscillospiraceae |
| ASV3244 | TRUE | FALSE | Only dry | *unknown* | Barnesiellaceae |
| ASV326 | TRUE | FALSE | Only dry | *UCG-005* | Oscillospiraceae |
| ASV3268 | TRUE | FALSE | Only dry | *unknown* | UCG-010 |
| ASV3288 | TRUE | FALSE | Only dry | *Marvinbryantia* | Lachnospiraceae |
| ASV329 | TRUE | FALSE | Only dry | *UCG-005* | Oscillospiraceae |
| ASV3310 | TRUE | FALSE | Only dry | *Lachnoclostridium* | Lachnospiraceae |
| ASV33215 | TRUE | FALSE | Only dry | *unknown* | Lachnospiraceae |
| ASV3322 | TRUE | FALSE | Only dry | *Salinimicrobium* | Flavobacteriaceae |
| ASV334 | TRUE | FALSE | Only dry | *Alistipes* | Rikenellaceae |
| ASV3345 | TRUE | FALSE | Only dry | *Comamonas* | Comamonadaceae |
| ASV33573 | TRUE | FALSE | Only dry | *unknown* | unknown |
| ASV3411 | TRUE | FALSE | Only dry | *Rikenellaceae RC9 gut group* | Rikenellaceae |
| ASV3412 | TRUE | FALSE | Only dry | *unknown* | Paludibacteraceae |
| ASV3427 | TRUE | FALSE | Only dry | *unknown* | UCG-010 |
| ASV34595 | TRUE | FALSE | Only dry | *unknown* | Devosiaceae |
| ASV34726 | TRUE | FALSE | Only dry | *Brumimicrobium* | Crocinitomicaceae |
| ASV34735 | TRUE | FALSE | Only dry | *unknown* | Micrococcaceae |
| ASV3483 | TRUE | FALSE | Only dry | *unknown* | UCG-010 |
| ASV34883 | TRUE | FALSE | Only dry | *unknown* | [Eubacterium] coprostanoligenes group |
| ASV34918 | TRUE | FALSE | Only dry | *unknown* | unknown |
| ASV3499 | TRUE | FALSE | Only dry | *unknown* | unknown |
| ASV3512 | TRUE | FALSE | Only dry | *unknown* | Micrococcaceae |
| ASV3542 | TRUE | FALSE | Only dry | *Alistipes* | Rikenellaceae |
| ASV357 | TRUE | FALSE | Only dry | *Salinicoccus* | Staphylococcaceae |
| ASV35718 | TRUE | FALSE | Only dry | *unknown* | Flavobacteriaceae |
| ASV3574 | TRUE | FALSE | Only dry | *Taibaiella* | Chitinophagaceae |
| ASV3589 | TRUE | FALSE | Only dry | *Lacticigenium* | Carnobacteriaceae |
| ASV35959 | TRUE | FALSE | Only dry | *unknown* | unknown |
| ASV35972 | TRUE | FALSE | Only dry | *Christensenellaceae R-7 group* | Christensenellaceae |
| ASV36151 | TRUE | FALSE | Only dry | *unknown* | unknown |
| ASV36196 | TRUE | FALSE | Only dry | *Garicola* | Micrococcaceae |
| ASV363 | TRUE | FALSE | Only dry | *Bacteroides* | Bacteroidaceae |
| ASV3648 | TRUE | FALSE | Only dry | *Alistipes* | Rikenellaceae |
| ASV365 | TRUE | FALSE | Only dry | *Solibacillus* | Planococcaceae |
| ASV366 | TRUE | FALSE | Only dry | *UCG-005* | Oscillospiraceae |
| ASV367 | TRUE | FALSE | Only dry | *unknown* | Pirellulaceae |
| ASV3677 | TRUE | FALSE | Only dry | *Anaeroplasma* | Acholeplasmataceae |
| ASV368 | TRUE | FALSE | Only dry | *UCG-005* | Oscillospiraceae |
| ASV3697 | TRUE | FALSE | Only dry | *[Eubacterium] siraeum group* | Ruminococcaceae |
| ASV37084 | TRUE | FALSE | Only dry | *IMCC26207* | Microtrichaceae |
| ASV37134 | TRUE | FALSE | Only dry | *unknown* | Muribaculaceae |
| ASV372 | TRUE | FALSE | Only dry | *Christensenellaceae R-7 group* | Christensenellaceae |
| ASV3752 | TRUE | FALSE | Only dry | *Virgibacillus* | Bacillaceae |
| ASV3762 | TRUE | FALSE | Only dry | *unknown* | unknown |
| ASV3775 | TRUE | FALSE | Only dry | *dgA-11 gut group* | Rikenellaceae |
| ASV3790 | TRUE | FALSE | Only dry | *GWE2-31-10* | Spirochaetaceae |
| ASV3807 | TRUE | FALSE | Only dry | *Glutamicibacter* | Micrococcaceae |
| ASV381 | TRUE | FALSE | Only dry | *unknown* | Lachnospiraceae |
| ASV3830 | TRUE | FALSE | Only dry | *Garicola* | Micrococcaceae |
| ASV3846 | TRUE | FALSE | Only dry | *Rikenellaceae RC9 gut group* | Rikenellaceae |
| ASV3851 | TRUE | FALSE | Only dry | *unknown* | UCG-010 |
| ASV3863 | TRUE | FALSE | Only dry | *unknown* | Lachnospiraceae |
| ASV389 | TRUE | FALSE | Only dry | *UCG-005* | Oscillospiraceae |
| ASV3984 | TRUE | FALSE | Only dry | *Alistipes* | Rikenellaceae |
| ASV3985 | TRUE | FALSE | Only dry | *unknown* | Cyclobacteriaceae |
| ASV4023 | TRUE | FALSE | Only dry | *unknown* | Paenibacillaceae |
| ASV4071 | TRUE | FALSE | Only dry | *Marvinbryantia* | Lachnospiraceae |
| ASV408 | TRUE | FALSE | Only dry | *unknown* | UCG-010 |
| ASV4126 | TRUE | FALSE | Only dry | *unknown* | Bacillaceae |
| ASV4142 | TRUE | FALSE | Only dry | *unknown* | Muribaculaceae |
| ASV4159 | TRUE | FALSE | Only dry | *unknown* | unknown |
| ASV4174 | TRUE | FALSE | Only dry | *Acholeplasma* | Acholeplasmataceae |
| ASV4234 | TRUE | FALSE | Only dry | *Selenomonas* | Selenomonadaceae |
| ASV4239 | TRUE | FALSE | Only dry | *Acinetobacter* | Moraxellaceae |
| ASV425 | TRUE | FALSE | Only dry | *Nocardioides* | Nocardioidaceae |
| ASV4257 | TRUE | FALSE | Only dry | *unknown* | Muribaculaceae |
| ASV4286 | TRUE | FALSE | Only dry | *dgA-11 gut group* | Rikenellaceae |
| ASV4319 | TRUE | FALSE | Only dry | *Clostridium sensu stricto 1* | Clostridiaceae |
| ASV4323 | TRUE | FALSE | Only dry | *unknown* | UCG-010 |
| ASV4343 | TRUE | FALSE | Only dry | *Alistipes* | Rikenellaceae |
| ASV4356 | TRUE | FALSE | Only dry | *Sphingobacterium* | Sphingobacteriaceae |
| ASV437 | TRUE | FALSE | Only dry | *UCG-005* | Oscillospiraceae |
| ASV4373 | TRUE | FALSE | Only dry | *Bacteroides* | Bacteroidaceae |
| ASV4391 | TRUE | FALSE | Only dry | *unknown* | unknown |
| ASV4397 | TRUE | FALSE | Only dry | *unknown* | p-251-o5 |
| ASV4399 | TRUE | FALSE | Only dry | *unknown* | Ruminococcaceae |
| ASV443 | TRUE | FALSE | Only dry | *Campylobacter* | Campylobacteraceae |
| ASV4529 | TRUE | FALSE | Only dry | *unknown* | unknown |
| ASV4530 | TRUE | FALSE | Only dry | *unknown* | unknown |
| ASV456 | TRUE | FALSE | Only dry | *Jonesia* | Jonesiaceae |
| ASV457 | TRUE | FALSE | Only dry | *Clostridium sensu stricto 1* | Clostridiaceae |
| ASV4615 | TRUE | FALSE | Only dry | *EMP-G18* | Acholeplasmataceae |
| ASV467 | TRUE | FALSE | Only dry | *Bacteroides* | Bacteroidaceae |
| ASV4705 | TRUE | FALSE | Only dry | *unknown* | Lachnospiraceae |
| ASV480 | TRUE | FALSE | Only dry | *UCG-009* | Butyricicoccaceae |
| ASV481 | TRUE | FALSE | Only dry | *unknown* | [Eubacterium] coprostanoligenes group |
| ASV4856 | TRUE | FALSE | Only dry | *Rikenellaceae RC9 gut group* | Rikenellaceae |
| ASV491 | TRUE | FALSE | Only dry | *Prevotella* | Prevotellaceae |
| ASV4941 | TRUE | FALSE | Only dry | *Vibrio* | Vibrionaceae |
| ASV496 | TRUE | FALSE | Only dry | *Acholeplasma* | Acholeplasmataceae |
| ASV5004 | TRUE | FALSE | Only dry | *Coprococcus* | Lachnospiraceae |
| ASV505 | TRUE | FALSE | Only dry | *Salinicoccus* | Staphylococcaceae |
| ASV509 | TRUE | FALSE | Only dry | *Lachnospiraceae AC2044 group* | Lachnospiraceae |
| ASV5124 | TRUE | FALSE | Only dry | *unknown* | UCG-010 |
| ASV5127 | TRUE | FALSE | Only dry | *Lachnospiraceae AC2044 group* | Lachnospiraceae |
| ASV5128 | TRUE | FALSE | Only dry | *TM7a* | Saccharimonadaceae |
| ASV516 | TRUE | FALSE | Only dry | *Aliidiomarina* | Idiomarinaceae |
| ASV518 | TRUE | FALSE | Only dry | *Corynebacterium* | Corynebacteriaceae |
| ASV5188 | TRUE | FALSE | Only dry | *Antricoccus* | Geodermatophilaceae |
| ASV519 | TRUE | FALSE | Only dry | *unknown* | unknown |
| ASV521 | TRUE | FALSE | Only dry | *unknown* | [Eubacterium] coprostanoligenes group |
| ASV5217 | TRUE | FALSE | Only dry | *Kurthia* | Planococcaceae |
| ASV5270 | TRUE | FALSE | Only dry | *Alkalibacterium* | Carnobacteriaceae |
| ASV5312 | TRUE | FALSE | Only dry | *Rikenellaceae RC9 gut group* | Rikenellaceae |
| ASV538 | TRUE | FALSE | Only dry | *Amphibacillus* | Bacillaceae |
| ASV5385 | TRUE | FALSE | Only dry | *Corynebacterium* | Corynebacteriaceae |
| ASV544 | TRUE | FALSE | Only dry | *Bacteroides* | Bacteroidaceae |
| ASV5506 | TRUE | FALSE | Only dry | *unknown* | UCG-010 |
| ASV552 | TRUE | FALSE | Only dry | *Prevotellaceae UCG-003* | Prevotellaceae |
| ASV5575 | TRUE | FALSE | Only dry | *Marinospirillum* | Halomonadaceae |
| ASV558 | TRUE | FALSE | Only dry | *Planktosalinus* | Flavobacteriaceae |
| ASV559 | TRUE | FALSE | Only dry | *unknown* | [Eubacterium] coprostanoligenes group |
| ASV5608 | TRUE | FALSE | Only dry | *Alistipes* | Rikenellaceae |
| ASV566 | TRUE | FALSE | Only dry | *Flavobacterium* | Flavobacteriaceae |
| ASV570 | TRUE | FALSE | Only dry | *Ruminobacter* | Succinivibrionaceae |
| ASV5740 | TRUE | FALSE | Only dry | *Pusillimonas* | Alcaligenaceae |
| ASV582 | TRUE | FALSE | Only dry | *UCG-005* | Oscillospiraceae |
| ASV583 | TRUE | FALSE | Only dry | *unknown* | Lachnospiraceae |
| ASV584 | TRUE | FALSE | Only dry | *Rikenellaceae RC9 gut group* | Rikenellaceae |
| ASV5852 | TRUE | FALSE | Only dry | *unknown* | Oscillospiraceae |
| ASV5880 | TRUE | FALSE | Only dry | *unknown* | Oscillospiraceae |
| ASV5921 | TRUE | FALSE | Only dry | *Papillibacter* | Oscillospiraceae |
| ASV5929 | TRUE | FALSE | Only dry | *unknown* | Ruminococcaceae |
| ASV593 | TRUE | FALSE | Only dry | *Lacticigenium* | Carnobacteriaceae |
| ASV5977 | TRUE | FALSE | Only dry | *Rikenellaceae RC9 gut group* | Rikenellaceae |
| ASV6018 | TRUE | FALSE | Only dry | *Streptomyces* | Streptomycetaceae |
| ASV6019 | TRUE | FALSE | Only dry | *Flavobacterium* | Flavobacteriaceae |
| ASV606 | TRUE | FALSE | Only dry | *UCG-005* | Oscillospiraceae |
| ASV6061 | TRUE | FALSE | Only dry | *C1-B045* | Porticoccaceae |
| ASV6070 | TRUE | FALSE | Only dry | *unknown* | Ruminococcaceae |
| ASV6078 | TRUE | FALSE | Only dry | *unknown* | unknown |
| ASV610 | TRUE | FALSE | Only dry | *UCG-005* | Oscillospiraceae |
| ASV612 | TRUE | FALSE | Only dry | *Marinimicrobium* | Cellvibrionaceae |
| ASV613 | TRUE | FALSE | Only dry | *Oceanobacillus* | Bacillaceae |
| ASV614 | TRUE | FALSE | Only dry | *Fermentimonas* | Dysgonomonadaceae |
| ASV620 | TRUE | FALSE | Only dry | *unknown* | unknown |
| ASV623 | TRUE | FALSE | Only dry | *unknown* | Lachnospiraceae |
| ASV6245 | TRUE | FALSE | Only dry | *unknown* | Bacillaceae |
| ASV627 | TRUE | FALSE | Only dry | *unknown* | Lachnospiraceae |
| ASV629 | TRUE | FALSE | Only dry | *unknown* | Ruminococcaceae |
| ASV6296 | TRUE | FALSE | Only dry | *unknown* | [Eubacterium] coprostanoligenes group |
| ASV630 | TRUE | FALSE | Only dry | *Facklamia* | Aerococcaceae |
| ASV633 | TRUE | FALSE | Only dry | *Atopostipes* | Carnobacteriaceae |
| ASV6337 | TRUE | FALSE | Only dry | *unknown* | unknown |
| ASV6377 | TRUE | FALSE | Only dry | *unknown* | Ruminococcaceae |
| ASV643 | TRUE | FALSE | Only dry | *Prevotella* | Prevotellaceae |
| ASV6433 | TRUE | FALSE | Only dry | *unknown* | Lachnospiraceae |
| ASV6446 | TRUE | FALSE | Only dry | *Rikenellaceae RC9 gut group* | Rikenellaceae |
| ASV648 | TRUE | FALSE | Only dry | *Rikenellaceae RC9 gut group* | Rikenellaceae |
| ASV6528 | TRUE | FALSE | Only dry | *Christensenellaceae R-7 group* | Christensenellaceae |
| ASV653 | TRUE | FALSE | Only dry | *Treponema* | Spirochaetaceae |
| ASV6594 | TRUE | FALSE | Only dry | *unknown* | Ruminococcaceae |
| ASV663 | TRUE | FALSE | Only dry | *unknown* | Lachnospiraceae |
| ASV6661 | TRUE | FALSE | Only dry | *unknown* | Sphingomonadaceae |
| ASV670 | TRUE | FALSE | Only dry | *Monoglobus* | Monoglobaceae |
| ASV675 | TRUE | FALSE | Only dry | *UCG-002* | Oscillospiraceae |
| ASV676 | TRUE | FALSE | Only dry | *Aliidiomarina* | Idiomarinaceae |
| ASV6802 | TRUE | FALSE | Only dry | *Marinobacter* | Marinobacteraceae |
| ASV681 | TRUE | FALSE | Only dry | *unknown* | Bradymonadaceae |
| ASV6810 | TRUE | FALSE | Only dry | *Oceanobacillus* | Bacillaceae |
| ASV6814 | TRUE | FALSE | Only dry | *unknown* | unknown |
| ASV6819 | TRUE | FALSE | Only dry | *dgA-11 gut group* | Rikenellaceae |
| ASV687 | TRUE | FALSE | Only dry | *Atopostipes* | Carnobacteriaceae |
| ASV688 | TRUE | FALSE | Only dry | *Rhodobaculum* | Rhodobacteraceae |
| ASV694 | TRUE | FALSE | Only dry | *Aliidiomarina* | Idiomarinaceae |
| ASV696 | TRUE | FALSE | Only dry | *TM7a* | Saccharimonadaceae |
| ASV703 | TRUE | FALSE | Only dry | *unknown* | JG30-KF-CM45 |
| ASV709 | TRUE | FALSE | Only dry | *TM7a* | Saccharimonadaceae |
| ASV714 | TRUE | FALSE | Only dry | *unknown* | Bacteroidales RF16 group |
| ASV7196 | TRUE | FALSE | Only dry | *Aliidiomarina* | Idiomarinaceae |
| ASV7211 | TRUE | FALSE | Only dry | *Anaerosporobacter* | Lachnospiraceae |
| ASV722 | TRUE | FALSE | Only dry | *Agathobacter* | Lachnospiraceae |
| ASV728 | TRUE | FALSE | Only dry | *Amphibacillus* | Bacillaceae |
| ASV729 | TRUE | FALSE | Only dry | *Marinimicrobium* | Cellvibrionaceae |
| ASV731 | TRUE | FALSE | Only dry | *unknown* | Lachnospiraceae |
| ASV733 | TRUE | FALSE | Only dry | *unknown* | [Eubacterium] coprostanoligenes group |
| ASV7358 | TRUE | FALSE | Only dry | *Citricoccus* | Micrococcaceae |
| ASV741 | TRUE | FALSE | Only dry | *unknown* | Muribaculaceae |
| ASV744 | TRUE | FALSE | Only dry | *Family XIII AD3011 group* | Anaerovoracaceae |
| ASV7461 | TRUE | FALSE | Only dry | *[Eubacterium] nodatum group* | Anaerovoracaceae |
| ASV750 | TRUE | FALSE | Only dry | *Prevotellaceae UCG-003* | Prevotellaceae |
| ASV7679 | TRUE | FALSE | Only dry | *Monoglobus* | Monoglobaceae |
| ASV772 | TRUE | FALSE | Only dry | *Acholeplasma* | Acholeplasmataceae |
| ASV7736 | TRUE | FALSE | Only dry | *unknown* | unknown |
| ASV778 | TRUE | FALSE | Only dry | *unknown* | unknown |
| ASV779 | TRUE | FALSE | Only dry | *Rikenellaceae RC9 gut group* | Rikenellaceae |
| ASV7844 | TRUE | FALSE | Only dry | *unknown* | Micrococcaceae |
| ASV786 | TRUE | FALSE | Only dry | *Brumimicrobium* | Crocinitomicaceae |
| ASV7865 | TRUE | FALSE | Only dry | *Erysipelotrichaceae UCG-007* | Erysipelotrichaceae |
| ASV787 | TRUE | FALSE | Only dry | *C1-B045* | Porticoccaceae |
| ASV791 | TRUE | FALSE | Only dry | *Prevotellaceae UCG-001* | Prevotellaceae |
| ASV794 | TRUE | FALSE | Only dry | *Bacteroides* | Bacteroidaceae |
| ASV7967 | TRUE | FALSE | Only dry | *[Eubacterium] tenue group* | Peptostreptococcaceae |
| ASV797 | TRUE | FALSE | Only dry | *UCG-005* | Oscillospiraceae |
| ASV807 | TRUE | FALSE | Only dry | *Escherichia-Shigella* | Enterobacteriaceae |
| ASV809 | TRUE | FALSE | Only dry | *unknown* | Tannerellaceae |
| ASV8094 | TRUE | FALSE | Only dry | *unknown* | UCG-010 |
| ASV810 | TRUE | FALSE | Only dry | *Bacteroides* | Bacteroidaceae |
| ASV8120 | TRUE | FALSE | Only dry | *unknown* | JG30-KF-CM45 |
| ASV815 | TRUE | FALSE | Only dry | *Jeotgalicoccus* | Staphylococcaceae |
| ASV8180 | TRUE | FALSE | Only dry | *UCG-005* | Oscillospiraceae |
| ASV822 | TRUE | FALSE | Only dry | *Rikenellaceae RC9 gut group* | Rikenellaceae |
| ASV824 | TRUE | FALSE | Only dry | *unknown* | Bradymonadaceae |
| ASV825 | TRUE | FALSE | Only dry | *unknown* | unknown |
| ASV8250 | TRUE | FALSE | Only dry | *Alkalibacterium* | Carnobacteriaceae |
| ASV827 | TRUE | FALSE | Only dry | *Atopostipes* | Carnobacteriaceae |
| ASV828 | TRUE | FALSE | Only dry | *Vibrio* | Vibrionaceae |
| ASV833 | TRUE | FALSE | Only dry | *unknown* | unknown |
| ASV835 | TRUE | FALSE | Only dry | *unknown* | UCG-010 |
| ASV841 | TRUE | FALSE | Only dry | *UCG-005* | Oscillospiraceae |
| ASV858 | TRUE | FALSE | Only dry | *Prevotellaceae UCG-003* | Prevotellaceae |
| ASV866 | TRUE | FALSE | Only dry | *Pseudogracilibacillus* | Bacillaceae |
| ASV870 | TRUE | FALSE | Only dry | *Marinobacter* | Marinobacteraceae |
| ASV8702 | TRUE | FALSE | Only dry | *unknown* | [Eubacterium] coprostanoligenes group |
| ASV871 | TRUE | FALSE | Only dry | *unknown* | Oscillospiraceae |
| ASV873 | TRUE | FALSE | Only dry | *Coprococcus* | Lachnospiraceae |
| ASV875 | TRUE | FALSE | Only dry | *unknown* | Ruminococcaceae |
| ASV879 | TRUE | FALSE | Only dry | *Bacteroides* | Bacteroidaceae |
| ASV880 | TRUE | FALSE | Only dry | *Prevotellaceae UCG-004* | Prevotellaceae |
| ASV882 | TRUE | FALSE | Only dry | *Prevotellaceae UCG-003* | Prevotellaceae |
| ASV8905 | TRUE | FALSE | Only dry | *unknown* | Bacillaceae |
| ASV892 | TRUE | FALSE | Only dry | *Comamonas* | Comamonadaceae |
| ASV899 | TRUE | FALSE | Only dry | *Halomonas* | Halomonadaceae |
| ASV9004 | TRUE | FALSE | Only dry | *unknown* | Fibrobacteraceae |
| ASV903 | TRUE | FALSE | Only dry | *Bacteroides* | Bacteroidaceae |
| ASV906 | TRUE | FALSE | Only dry | *unknown* | Paludibacteraceae |
| ASV908 | TRUE | FALSE | Only dry | *Bacteroides* | Bacteroidaceae |
| ASV909 | TRUE | FALSE | Only dry | *unknown* | Streptomycetaceae |
| ASV913 | TRUE | FALSE | Only dry | *Candidatus Soleaferrea* | Ruminococcaceae |
| ASV916 | TRUE | FALSE | Only dry | *Alistipes* | Rikenellaceae |
| ASV922 | TRUE | FALSE | Only dry | *Bacteroides* | Bacteroidaceae |
| ASV923 | TRUE | FALSE | Only dry | *unknown* | [Eubacterium] coprostanoligenes group |
| ASV9251 | TRUE | FALSE | Only dry | *Alloprevotella* | Prevotellaceae |
| ASV9339 | TRUE | FALSE | Only dry | *unknown* | [Eubacterium] coprostanoligenes group |
| ASV937 | TRUE | FALSE | Only dry | *Tissierella* | Family XI |
| ASV9372 | TRUE | FALSE | Only dry | *unknown* | UCG-010 |
| ASV938 | TRUE | FALSE | Only dry | *Mailhella* | Desulfovibrionaceae |
| ASV9461 | TRUE | FALSE | Only dry | *W5053* | Family XI |
| ASV9478 | TRUE | FALSE | Only dry | *Rikenellaceae RC9 gut group* | Rikenellaceae |
| ASV953 | TRUE | FALSE | Only dry | *Monoglobus* | Monoglobaceae |
| ASV959 | TRUE | FALSE | Only dry | *unknown* | unknown |
| ASV9627 | TRUE | FALSE | Only dry | *[Eubacterium] hallii group* | Lachnospiraceae |
| ASV9779 | TRUE | FALSE | Only dry | *unknown* | Sphingobacteriaceae |
| ASV981 | TRUE | FALSE | Only dry | *Lachnospiraceae UCG-001* | Lachnospiraceae |
| ASV985 | TRUE | FALSE | Only dry | *Alistipes* | Rikenellaceae |
| ASV988 | TRUE | FALSE | Only dry | *Christensenellaceae R-7 group* | Christensenellaceae |
| ASV992 | TRUE | FALSE | Only dry | *Christensenellaceae R-7 group* | Christensenellaceae |
| ASV996 | TRUE | FALSE | Only dry | *Amphibacillus* | Bacillaceae |
| ASV9960 | TRUE | FALSE | Only dry | *unknown* | unknown |
| ASV9966 | TRUE | FALSE | Only dry | *unknown* | Idiomarinaceae |
| ASV998 | TRUE | FALSE | Only dry | *Prevotellaceae UCG-003* | Prevotellaceae |
| ASV10000 | FALSE | TRUE | Only wet | *Monoglobus* | Monoglobaceae |
| ASV1009 | FALSE | TRUE | Only wet | *Pseudoxanthomonas* | Xanthomonadaceae |
| ASV1013 | FALSE | TRUE | Only wet | *Bacillus* | Bacillaceae |
| ASV1014 | FALSE | TRUE | Only wet | *unknown* | Longimicrobiaceae |
| ASV1035 | FALSE | TRUE | Only wet | *unknown* | Bacillaceae |
| ASV1037 | FALSE | TRUE | Only wet | *unknown* | Xanthomonadaceae |
| ASV10484 | FALSE | TRUE | Only wet | *unknown* | Muribaculaceae |
| ASV1061 | FALSE | TRUE | Only wet | *Gracilibacillus* | Bacillaceae |
| ASV10699 | FALSE | TRUE | Only wet | *unknown* | [Eubacterium] coprostanoligenes group |
| ASV1074 | FALSE | TRUE | Only wet | *unknown* | Lachnospiraceae |
| ASV1076 | FALSE | TRUE | Only wet | *Paenibacillus* | Paenibacillaceae |
| ASV10989 | FALSE | TRUE | Only wet | *Brevibacillus* | Brevibacillaceae |
| ASV1099 | FALSE | TRUE | Only wet | *Atopostipes* | Carnobacteriaceae |
| ASV11023 | FALSE | TRUE | Only wet | *unknown* | Bacillaceae |
| ASV1106 | FALSE | TRUE | Only wet | *unknown* | unknown |
| ASV1116 | FALSE | TRUE | Only wet | *unknown* | Marinococcaceae |
| ASV112 | FALSE | TRUE | Only wet | *Ornithinibacillus* | Bacillaceae |
| ASV1123 | FALSE | TRUE | Only wet | *Ornithinibacillus* | Bacillaceae |
| ASV1124 | FALSE | TRUE | Only wet | *Pseudomonas* | Pseudomonadaceae |
| ASV11271 | FALSE | TRUE | Only wet | *Prevotellaceae UCG-001* | Prevotellaceae |
| ASV11276 | FALSE | TRUE | Only wet | *Acholeplasma* | Acholeplasmataceae |
| ASV1129 | FALSE | TRUE | Only wet | *Sinibacillus* | Bacillaceae |
| ASV11440 | FALSE | TRUE | Only wet | *unknown* | unknown |
| ASV11673 | FALSE | TRUE | Only wet | *Akkermansia* | Akkermansiaceae |
| ASV11679 | FALSE | TRUE | Only wet | *unknown* | unknown |
| ASV1170 | FALSE | TRUE | Only wet | *Pseudogracilibacillus* | Bacillaceae |
| ASV1176 | FALSE | TRUE | Only wet | *unknown* | Bacillaceae |
| ASV1191 | FALSE | TRUE | Only wet | *unknown* | Paenibacillaceae |
| ASV11928 | FALSE | TRUE | Only wet | *Oceanobacillus* | Bacillaceae |
| ASV11989 | FALSE | TRUE | Only wet | *Alistipes* | Rikenellaceae |
| ASV120 | FALSE | TRUE | Only wet | *unknown* | Fodinicurvataceae |
| ASV12004 | FALSE | TRUE | Only wet | *unknown* | Rikenellaceae |
| ASV1215 | FALSE | TRUE | Only wet | *unknown* | unknown |
| ASV123 | FALSE | TRUE | Only wet | *unknown* | Bacillaceae |
| ASV12327 | FALSE | TRUE | Only wet | *Pseudoxanthomonas* | Xanthomonadaceae |
| ASV1239 | FALSE | TRUE | Only wet | *Brevibacillus* | Brevibacillaceae |
| ASV125 | FALSE | TRUE | Only wet | *Oceanobacillus* | Bacillaceae |
| ASV1252 | FALSE | TRUE | Only wet | *Cellvibrio* | Cellvibrionaceae |
| ASV1263 | FALSE | TRUE | Only wet | *Alloiococcus* | Carnobacteriaceae |
| ASV1268 | FALSE | TRUE | Only wet | *Gracilibacillus* | Bacillaceae |
| ASV1279 | FALSE | TRUE | Only wet | *unknown* | Propionibacteriaceae |
| ASV1281 | FALSE | TRUE | Only wet | *Gracilibacillus* | Bacillaceae |
| ASV1283 | FALSE | TRUE | Only wet | *unknown* | Paenibacillaceae |
| ASV129 | FALSE | TRUE | Only wet | *unknown* | Bacillaceae |
| ASV1295 | FALSE | TRUE | Only wet | *unknown* | Paenibacillaceae |
| ASV1298 | FALSE | TRUE | Only wet | *Alkalibacterium* | Carnobacteriaceae |
| ASV1300 | FALSE | TRUE | Only wet | *Rikenellaceae RC9 gut group* | Rikenellaceae |
| ASV13182 | FALSE | TRUE | Only wet | *Pseudomonas* | Pseudomonadaceae |
| ASV1324 | FALSE | TRUE | Only wet | *Treponema* | Spirochaetaceae |
| ASV134 | FALSE | TRUE | Only wet | *Marinococcus* | Marinococcaceae |
| ASV1343 | FALSE | TRUE | Only wet | *unknown* | MWH-CFBk5 |
| ASV1345 | FALSE | TRUE | Only wet | *Salipaludibacillus* | Salisediminibacteriaceae |
| ASV135 | FALSE | TRUE | Only wet | *unknown* | Bacillaceae |
| ASV1356 | FALSE | TRUE | Only wet | *Ornithinibacillus* | Bacillaceae |
| ASV137 | FALSE | TRUE | Only wet | *Gracilibacillus* | Bacillaceae |
| ASV1371 | FALSE | TRUE | Only wet | *Atopostipes* | Carnobacteriaceae |
| ASV1373 | FALSE | TRUE | Only wet | *unknown* | Cellvibrionaceae |
| ASV1375 | FALSE | TRUE | Only wet | *Oceanobacillus* | Bacillaceae |
| ASV1392 | FALSE | TRUE | Only wet | *unknown* | unknown |
| ASV1403 | FALSE | TRUE | Only wet | *Oceanobacillus* | Bacillaceae |
| ASV1416 | FALSE | TRUE | Only wet | *Paenibacillus* | Paenibacillaceae |
| ASV14207 | FALSE | TRUE | Only wet | *UCG-009* | Butyricicoccaceae |
| ASV14209 | FALSE | TRUE | Only wet | *Stenotrophomonas* | Xanthomonadaceae |
| ASV144 | FALSE | TRUE | Only wet | *Ornithinibacillus* | Bacillaceae |
| ASV14607 | FALSE | TRUE | Only wet | *unknown* | unknown |
| ASV14683 | FALSE | TRUE | Only wet | *Marinimicrobium* | Cellvibrionaceae |
| ASV14732 | FALSE | TRUE | Only wet | *unknown* | Victivallaceae |
| ASV1485 | FALSE | TRUE | Only wet | *[Eubacterium] brachy group* | Anaerovoracaceae |
| ASV1499 | FALSE | TRUE | Only wet | *unknown* | Eggerthellaceae |
| ASV150 | FALSE | TRUE | Only wet | *DSSF69* | Sphingomonadaceae |
| ASV15046 | FALSE | TRUE | Only wet | *Monoglobus* | Monoglobaceae |
| ASV1515 | FALSE | TRUE | Only wet | *Oceanobacillus* | Bacillaceae |
| ASV1517 | FALSE | TRUE | Only wet | *Saccharopolyspora* | Pseudonocardiaceae |
| ASV152 | FALSE | TRUE | Only wet | *Hahella* | Hahellaceae |
| ASV1523 | FALSE | TRUE | Only wet | *Vulgatibacter* | Vulgatibacteraceae |
| ASV1527 | FALSE | TRUE | Only wet | *Ornithinibacillus* | Bacillaceae |
| ASV1541 | FALSE | TRUE | Only wet | *unknown* | [Eubacterium] coprostanoligenes group |
| ASV1549 | FALSE | TRUE | Only wet | *unknown* | unknown |
| ASV15492 | FALSE | TRUE | Only wet | *unknown* | Peptostreptococcaceae |
| ASV15608 | FALSE | TRUE | Only wet | *unknown* | unknown |
| ASV1566 | FALSE | TRUE | Only wet | *Natronococcus* | Haloferacaceae |
| ASV15751 | FALSE | TRUE | Only wet | *unknown* | unknown |
| ASV159 | FALSE | TRUE | Only wet | *Oceanobacillus* | Bacillaceae |
| ASV1596 | FALSE | TRUE | Only wet | *unknown* | Longimicrobiaceae |
| ASV1608 | FALSE | TRUE | Only wet | *Devosia* | Devosiaceae |
| ASV1613 | FALSE | TRUE | Only wet | *Halobacillus* | Bacillaceae |
| ASV1617 | FALSE | TRUE | Only wet | *unknown* | BIrii41 |
| ASV1619 | FALSE | TRUE | Only wet | *Aequorivita* | Flavobacteriaceae |
| ASV1633 | FALSE | TRUE | Only wet | *unknown* | F082 |
| ASV1637 | FALSE | TRUE | Only wet | *Kroppenstedtia* | Thermoactinomycetaceae |
| ASV1641 | FALSE | TRUE | Only wet | *Tessaracoccus* | Propionibacteriaceae |
| ASV1654 | FALSE | TRUE | Only wet | *Thermobacillus* | Paenibacillaceae |
| ASV16651 | FALSE | TRUE | Only wet | *Sphingobacterium* | Sphingobacteriaceae |
| ASV16662 | FALSE | TRUE | Only wet | *unknown* | unknown |
| ASV1667 | FALSE | TRUE | Only wet | *Bacillus* | Bacillaceae |
| ASV1676 | FALSE | TRUE | Only wet | *Pusillimonas* | Alcaligenaceae |
| ASV1677 | FALSE | TRUE | Only wet | *Alkalibacterium* | Carnobacteriaceae |
| ASV168 | FALSE | TRUE | Only wet | *Ornithinibacillus* | Bacillaceae |
| ASV1685 | FALSE | TRUE | Only wet | *Haloplasma* | Haloplasmataceae |
| ASV170 | FALSE | TRUE | Only wet | *Saccharomonospora* | Pseudonocardiaceae |
| ASV1701 | FALSE | TRUE | Only wet | *unknown* | Rhodothermaceae |
| ASV171 | FALSE | TRUE | Only wet | *unknown* | unknown |
| ASV1716 | FALSE | TRUE | Only wet | *unknown* | Cyclobacteriaceae |
| ASV1777 | FALSE | TRUE | Only wet | *unknown* | Marinococcaceae |
| ASV1783 | FALSE | TRUE | Only wet | *unknown* | Paenibacillaceae |
| ASV179 | FALSE | TRUE | Only wet | *unknown* | Bacillaceae |
| ASV17977 | FALSE | TRUE | Only wet | *unknown* | Planococcaceae |
| ASV17985 | FALSE | TRUE | Only wet | *Sphingobacterium* | Sphingobacteriaceae |
| ASV1804 | FALSE | TRUE | Only wet | *Coprococcus* | Lachnospiraceae |
| ASV1806 | FALSE | TRUE | Only wet | *Thermoactinomyces* | Thermoactinomycetaceae |
| ASV1807 | FALSE | TRUE | Only wet | *Bhargavaea* | Planococcaceae |
| ASV18157 | FALSE | TRUE | Only wet | *unknown* | Clostridiaceae |
| ASV18167 | FALSE | TRUE | Only wet | *Turicibacter* | Erysipelotrichaceae |
| ASV1826 | FALSE | TRUE | Only wet | *unknown* | unknown |
| ASV188 | FALSE | TRUE | Only wet | *unknown* | Bacillaceae |
| ASV1881 | FALSE | TRUE | Only wet | *unknown* | MWH-CFBk5 |
| ASV1888 | FALSE | TRUE | Only wet | *Paenibacillus* | Paenibacillaceae |
| ASV1907 | FALSE | TRUE | Only wet | *Bacillus* | Bacillaceae |
| ASV19292 | FALSE | TRUE | Only wet | *unknown* | unknown |
| ASV193 | FALSE | TRUE | Only wet | *Halomonas* | Halomonadaceae |
| ASV1936 | FALSE | TRUE | Only wet | *Salinimicrobium* | Flavobacteriaceae |
| ASV1937 | FALSE | TRUE | Only wet | *Georgenia* | Bogoriellaceae |
| ASV19418 | FALSE | TRUE | Only wet | *unknown* | Rhodocyclaceae |
| ASV19421 | FALSE | TRUE | Only wet | *unknown* | unknown |
| ASV1949 | FALSE | TRUE | Only wet | *unknown* | UCG-010 |
| ASV1954 | FALSE | TRUE | Only wet | *Pseudohongiella* | Pseudohongiellaceae |
| ASV197 | FALSE | TRUE | Only wet | *unknown* | Pseudomonadaceae |
| ASV198 | FALSE | TRUE | Only wet | *Cellvibrio* | Cellvibrionaceae |
| ASV1994 | FALSE | TRUE | Only wet | *[Ruminococcus] gauvreauii group* | Lachnospiraceae |
| ASV20236 | FALSE | TRUE | Only wet | *unknown* | unknown |
| ASV20248 | FALSE | TRUE | Only wet | *Atopostipes* | Carnobacteriaceae |
| ASV2032 | FALSE | TRUE | Only wet | *Ruminococcus* | Ruminococcaceae |
| ASV20400 | FALSE | TRUE | Only wet | *unknown* | [Eubacterium] coprostanoligenes group |
| ASV2078 | FALSE | TRUE | Only wet | *unknown* | Bradymonadaceae |
| ASV2079 | FALSE | TRUE | Only wet | *unknown* | Microbacteriaceae |
| ASV2103 | FALSE | TRUE | Only wet | *Sinibacillus* | Bacillaceae |
| ASV2108 | FALSE | TRUE | Only wet | *Allorhizobium-Neorhizobium-Pararhizobium-Rhizobium* | Rhizobiaceae |
| ASV21169 | FALSE | TRUE | Only wet | *unknown* | unknown |
| ASV212 | FALSE | TRUE | Only wet | *Truepera* | Trueperaceae |
| ASV21228 | FALSE | TRUE | Only wet | *Aliicoccus* | Staphylococcaceae |
| ASV2136 | FALSE | TRUE | Only wet | *unknown* | Bradymonadaceae |
| ASV21386 | FALSE | TRUE | Only wet | *unknown* | unknown |
| ASV214 | FALSE | TRUE | Only wet | *Parapusillimonas* | Alcaligenaceae |
| ASV2142 | FALSE | TRUE | Only wet | *Thiomicrorhabdus* | Thiomicrospiraceae |
| ASV21422 | FALSE | TRUE | Only wet | *unknown* | Bacillaceae |
| ASV21431 | FALSE | TRUE | Only wet | *unknown* | Staphylococcaceae |
| ASV21575 | FALSE | TRUE | Only wet | *unknown* | Corynebacteriaceae |
| ASV216 | FALSE | TRUE | Only wet | *Aminobacter* | Rhizobiaceae |
| ASV2166 | FALSE | TRUE | Only wet | *Methylophaga* | Methylophagaceae |
| ASV2201 | FALSE | TRUE | Only wet | *unknown* | Alkaliphilus |
| ASV2237 | FALSE | TRUE | Only wet | *unknown* | Muribaculaceae |
| ASV2252 | FALSE | TRUE | Only wet | *Novibacillus* | Thermoactinomycetaceae |
| ASV2262 | FALSE | TRUE | Only wet | *unknown* | Weeksellaceae |
| ASV2275 | FALSE | TRUE | Only wet | *Novibacillus* | Thermoactinomycetaceae |
| ASV228 | FALSE | TRUE | Only wet | *Thermobacillus* | Paenibacillaceae |
| ASV229 | FALSE | TRUE | Only wet | *Georgenia* | Bogoriellaceae |
| ASV23260 | FALSE | TRUE | Only wet | *Sphingobacterium* | Sphingobacteriaceae |
| ASV23276 | FALSE | TRUE | Only wet | *unknown* | unknown |
| ASV23291 | FALSE | TRUE | Only wet | *unknown* | Bacillaceae |
| ASV2330 | FALSE | TRUE | Only wet | *Virgibacillus* | Bacillaceae |
| ASV234 | FALSE | TRUE | Only wet | *unknown* | Bacillaceae |
| ASV235 | FALSE | TRUE | Only wet | *Lysobacter* | Xanthomonadaceae |
| ASV2388 | FALSE | TRUE | Only wet | *unknown* | Alcaligenaceae |
| ASV23976 | FALSE | TRUE | Only wet | *unknown* | Lachnospiraceae |
| ASV23992 | FALSE | TRUE | Only wet | *unknown* | unknown |
| ASV240 | FALSE | TRUE | Only wet | *unknown* | JG30-KF-CM45 |
| ASV2403 | FALSE | TRUE | Only wet | *unknown* | Cellvibrionaceae |
| ASV244 | FALSE | TRUE | Only wet | *Paucisalibacillus* | Bacillaceae |
| ASV248 | FALSE | TRUE | Only wet | *Thermobacillus* | Paenibacillaceae |
| ASV2492 | FALSE | TRUE | Only wet | *unknown* | Alkaliphilus |
| ASV2509 | FALSE | TRUE | Only wet | *unknown* | Bacillaceae |
| ASV2528 | FALSE | TRUE | Only wet | *Acholeplasma* | Acholeplasmataceae |
| ASV2559 | FALSE | TRUE | Only wet | *unknown* | Paenibacillaceae |
| ASV257 | FALSE | TRUE | Only wet | *unknown* | Bacillaceae |
| ASV26011 | FALSE | TRUE | Only wet | *unknown* | Bacillaceae |
| ASV2602 | FALSE | TRUE | Only wet | *Cellvibrio* | Cellvibrionaceae |
| ASV26035 | FALSE | TRUE | Only wet | *unknown* | Ruminococcaceae |
| ASV2610 | FALSE | TRUE | Only wet | *unknown* | UCG-010 |
| ASV2629 | FALSE | TRUE | Only wet | *Oscillibacter* | Oscillospiraceae |
| ASV26403 | FALSE | TRUE | Only wet | *unknown* | Staphylococcaceae |
| ASV2649 | FALSE | TRUE | Only wet | *unknown* | Bacillaceae |
| ASV266 | FALSE | TRUE | Only wet | *Bacillus* | Bacillaceae |
| ASV26689 | FALSE | TRUE | Only wet | *unknown* | unknown |
| ASV26750 | FALSE | TRUE | Only wet | *unknown* | unknown |
| ASV2685 | FALSE | TRUE | Only wet | *unknown* | Bacillaceae |
| ASV2699 | FALSE | TRUE | Only wet | *unknown* | Lachnospiraceae |
| ASV2775 | FALSE | TRUE | Only wet | *unknown* | LWQ8 |
| ASV2786 | FALSE | TRUE | Only wet | *Gracilibacillus* | Bacillaceae |
| ASV2802 | FALSE | TRUE | Only wet | *unknown* | unknown |
| ASV2830 | FALSE | TRUE | Only wet | *unknown* | Bacillaceae |
| ASV28463 | FALSE | TRUE | Only wet | *unknown* | unknown |
| ASV28497 | FALSE | TRUE | Only wet | *unknown* | Idiomarinaceae |
| ASV2884 | FALSE | TRUE | Only wet | *Treponema* | Spirochaetaceae |
| ASV2911 | FALSE | TRUE | Only wet | *Vibrio* | Vibrionaceae |
| ASV29237 | FALSE | TRUE | Only wet | *Flaviflexus* | Actinomycetaceae |
| ASV29241 | FALSE | TRUE | Only wet | *unknown* | Carnobacteriaceae |
| ASV29243 | FALSE | TRUE | Only wet | *unknown* | Bacillaceae |
| ASV2926 | FALSE | TRUE | Only wet | *unknown* | Limnochordaceae |
| ASV2963 | FALSE | TRUE | Only wet | *unknown* | Sandaracinaceae |
| ASV2979 | FALSE | TRUE | Only wet | *unknown* | [Eubacterium] coprostanoligenes group |
| ASV2982 | FALSE | TRUE | Only wet | *unknown* | UCG-010 |
| ASV300 | FALSE | TRUE | Only wet | *Piscibacillus* | Bacillaceae |
| ASV3010 | FALSE | TRUE | Only wet | *unknown* | Bacillaceae |
| ASV3019 | FALSE | TRUE | Only wet | *Akkermansia* | Akkermansiaceae |
| ASV303 | FALSE | TRUE | Only wet | *Pusillimonas* | Alcaligenaceae |
| ASV30596 | FALSE | TRUE | Only wet | *unknown* | unknown |
| ASV30632 | FALSE | TRUE | Only wet | *unknown* | unknown |
| ASV30837 | FALSE | TRUE | Only wet | *unknown* | Ruminococcaceae |
| ASV309 | FALSE | TRUE | Only wet | *Pusillimonas* | Alcaligenaceae |
| ASV31 | FALSE | TRUE | Only wet | *unknown* | unknown |
| ASV3106 | FALSE | TRUE | Only wet | *unknown* | Bacillaceae |
| ASV3108 | FALSE | TRUE | Only wet | *Comamonas* | Comamonadaceae |
| ASV31119 | FALSE | TRUE | Only wet | *Atopostipes* | Carnobacteriaceae |
| ASV31124 | FALSE | TRUE | Only wet | *unknown* | Carnobacteriaceae |
| ASV3116 | FALSE | TRUE | Only wet | *Anaerobacillus* | Bacillaceae |
| ASV3117 | FALSE | TRUE | Only wet | *Monoglobus* | Monoglobaceae |
| ASV3179 | FALSE | TRUE | Only wet | *Truepera* | Trueperaceae |
| ASV319 | FALSE | TRUE | Only wet | *unknown* | Bacillaceae |
| ASV3208 | FALSE | TRUE | Only wet | *Guyparkeria* | Thioalkalibacteraceae |
| ASV32423 | FALSE | TRUE | Only wet | *unknown* | UCG-010 |
| ASV325 | FALSE | TRUE | Only wet | *unknown* | unknown |
| ASV3261 | FALSE | TRUE | Only wet | *unknown* | Paenibacillaceae |
| ASV3280 | FALSE | TRUE | Only wet | *Steroidobacter* | Steroidobacteraceae |
| ASV3309 | FALSE | TRUE | Only wet | *unknown* | Bacillaceae |
| ASV333 | FALSE | TRUE | Only wet | *Bacillus* | Bacillaceae |
| ASV3332 | FALSE | TRUE | Only wet | *Haloplasma* | Haloplasmataceae |
| ASV33472 | FALSE | TRUE | Only wet | *unknown* | unknown |
| ASV33489 | FALSE | TRUE | Only wet | *unknown* | UCG-010 |
| ASV33497 | FALSE | TRUE | Only wet | *unknown* | Micrococcaceae |
| ASV335 | FALSE | TRUE | Only wet | *unknown* | Bacillaceae |
| ASV3356 | FALSE | TRUE | Only wet | *unknown* | Paenibacillaceae |
| ASV3393 | FALSE | TRUE | Only wet | *unknown* | unknown |
| ASV34 | FALSE | TRUE | Only wet | *Bhargavaea* | Planococcaceae |
| ASV3402 | FALSE | TRUE | Only wet | *unknown* | unknown |
| ASV3471 | FALSE | TRUE | Only wet | *unknown* | Bacillaceae |
| ASV34817 | FALSE | TRUE | Only wet | *unknown* | Ruminococcaceae |
| ASV34970 | FALSE | TRUE | Only wet | *Cryomorpha* | Cryomorphaceae |
| ASV3509 | FALSE | TRUE | Only wet | *Ureibacillus* | Bacillaceae |
| ASV353 | FALSE | TRUE | Only wet | *Lysinibacillus* | Planococcaceae |
| ASV3532 | FALSE | TRUE | Only wet | *Gracilibacillus* | Bacillaceae |
| ASV3543 | FALSE | TRUE | Only wet | *Oceanobacillus* | Bacillaceae |
| ASV358 | FALSE | TRUE | Only wet | *Dietzia* | Dietziaceae |
| ASV3583 | FALSE | TRUE | Only wet | *unknown* | Paenibacillaceae |
| ASV3585 | FALSE | TRUE | Only wet | *Globicatella* | Aerococcaceae |
| ASV3602 | FALSE | TRUE | Only wet | *unknown* | unknown |
| ASV36255 | FALSE | TRUE | Only wet | *unknown* | Barnesiellaceae |
| ASV3681 | FALSE | TRUE | Only wet | *unknown* | Haloferacaceae |
| ASV3700 | FALSE | TRUE | Only wet | *Anaerobacillus* | Bacillaceae |
| ASV3756 | FALSE | TRUE | Only wet | *unknown* | Alcaligenaceae |
| ASV3801 | FALSE | TRUE | Only wet | *[Eubacterium] siraeum group* | Ruminococcaceae |
| ASV38193 | FALSE | TRUE | Only wet | *Nesterenkonia* | Micrococcaceae |
| ASV383 | FALSE | TRUE | Only wet | *unknown* | Bacillaceae |
| ASV395 | FALSE | TRUE | Only wet | *Altererythrobacter* | Sphingomonadaceae |
| ASV409 | FALSE | TRUE | Only wet | *Ornithinibacillus* | Bacillaceae |
| ASV412 | FALSE | TRUE | Only wet | *Virgibacillus* | Bacillaceae |
| ASV4317 | FALSE | TRUE | Only wet | *Aliidiomarina* | Idiomarinaceae |
| ASV4345 | FALSE | TRUE | Only wet | *Pelagibacterium* | Devosiaceae |
| ASV4395 | FALSE | TRUE | Only wet | *Natronococcus* | Haloferacaceae |
| ASV440 | FALSE | TRUE | Only wet | *Paenibacillus* | Paenibacillaceae |
| ASV447 | FALSE | TRUE | Only wet | *Lentibacillus* | Bacillaceae |
| ASV449 | FALSE | TRUE | Only wet | *Thermobacillus* | Paenibacillaceae |
| ASV450 | FALSE | TRUE | Only wet | *Pseudomonas* | Pseudomonadaceae |
| ASV4511 | FALSE | TRUE | Only wet | *Pseudomonas* | Pseudomonadaceae |
| ASV4513 | FALSE | TRUE | Only wet | *Prevotellaceae UCG-003* | Prevotellaceae |
| ASV462 | FALSE | TRUE | Only wet | *unknown* | Bacillaceae |
| ASV4636 | FALSE | TRUE | Only wet | *Flavobacterium* | Flavobacteriaceae |
| ASV4665 | FALSE | TRUE | Only wet | *Brevibacterium* | Brevibacteriaceae |
| ASV4681 | FALSE | TRUE | Only wet | *unknown* | unknown |
| ASV48 | FALSE | TRUE | Only wet | *Domibacillus* | Planococcaceae |
| ASV4815 | FALSE | TRUE | Only wet | *NK4A214 group* | Oscillospiraceae |
| ASV483 | FALSE | TRUE | Only wet | *Novibacillus* | Thermoactinomycetaceae |
| ASV488 | FALSE | TRUE | Only wet | *Salinicoccus* | Staphylococcaceae |
| ASV4898 | FALSE | TRUE | Only wet | *unknown* | Muribaculaceae |
| ASV497 | FALSE | TRUE | Only wet | *Bacillus* | Bacillaceae |
| ASV50 | FALSE | TRUE | Only wet | *Ornithinibacillus* | Bacillaceae |
| ASV5015 | FALSE | TRUE | Only wet | *unknown* | Bacillaceae |
| ASV5105 | FALSE | TRUE | Only wet | *unknown* | unknown |
| ASV514 | FALSE | TRUE | Only wet | *unknown* | Cellvibrionaceae |
| ASV5186 | FALSE | TRUE | Only wet | *unknown* | Rhodobacteraceae |
| ASV5228 | FALSE | TRUE | Only wet | *Prevotella* | Prevotellaceae |
| ASV5318 | FALSE | TRUE | Only wet | *unknown* | Lachnospiraceae |
| ASV5320 | FALSE | TRUE | Only wet | *unknown* | unknown |
| ASV535 | FALSE | TRUE | Only wet | *Georgenia* | Bogoriellaceae |
| ASV5386 | FALSE | TRUE | Only wet | *unknown* | Bacillaceae |
| ASV5395 | FALSE | TRUE | Only wet | *unknown* | Rhodobacteraceae |
| ASV54 | FALSE | TRUE | Only wet | *Melghirimyces* | Thermoactinomycetaceae |
| ASV5417 | FALSE | TRUE | Only wet | *Ruminobacter* | Succinivibrionaceae |
| ASV542 | FALSE | TRUE | Only wet | *Monoglobus* | Monoglobaceae |
| ASV548 | FALSE | TRUE | Only wet | *Pusillimonas* | Alcaligenaceae |
| ASV5616 | FALSE | TRUE | Only wet | *Sporosarcina* | Planococcaceae |
| ASV5646 | FALSE | TRUE | Only wet | *unknown* | unknown |
| ASV5660 | FALSE | TRUE | Only wet | *Bacillus* | Bacillaceae |
| ASV5666 | FALSE | TRUE | Only wet | *Cellvibrio* | Cellvibrionaceae |
| ASV574 | FALSE | TRUE | Only wet | *Salinicoccus* | Staphylococcaceae |
| ASV577 | FALSE | TRUE | Only wet | *Ureibacillus* | Bacillaceae |
| ASV5783 | FALSE | TRUE | Only wet | *unknown* | Bacillaceae |
| ASV595 | FALSE | TRUE | Only wet | *Thermobifida* | Nocardiopsaceae |
| ASV603 | FALSE | TRUE | Only wet | *Turicibacter* | Erysipelotrichaceae |
| ASV604 | FALSE | TRUE | Only wet | *unknown* | unknown |
| ASV6170 | FALSE | TRUE | Only wet | *Lentibacillus* | Bacillaceae |
| ASV619 | FALSE | TRUE | Only wet | *Virgibacillus* | Bacillaceae |
| ASV6190 | FALSE | TRUE | Only wet | *unknown* | UCG-010 |
| ASV625 | FALSE | TRUE | Only wet | *Gracilibacillus* | Bacillaceae |
| ASV628 | FALSE | TRUE | Only wet | *Blastopirellula* | Pirellulaceae |
| ASV6299 | FALSE | TRUE | Only wet | *unknown* | Bacillaceae |
| ASV6306 | FALSE | TRUE | Only wet | *Saccharomonospora* | Pseudonocardiaceae |
| ASV6341 | FALSE | TRUE | Only wet | *unknown* | UCG-010 |
| ASV6375 | FALSE | TRUE | Only wet | *Oceanobacillus* | Bacillaceae |
| ASV6438 | FALSE | TRUE | Only wet | *Ornithinibacillus* | Bacillaceae |
| ASV6440 | FALSE | TRUE | Only wet | *unknown* | unknown |
| ASV646 | FALSE | TRUE | Only wet | *Truepera* | Trueperaceae |
| ASV6501 | FALSE | TRUE | Only wet | *unknown* | Bacillaceae |
| ASV6576 | FALSE | TRUE | Only wet | *unknown* | Ruminococcaceae |
| ASV658 | FALSE | TRUE | Only wet | *Pusillimonas* | Alcaligenaceae |
| ASV664 | FALSE | TRUE | Only wet | *Longispora* | Micromonosporaceae |
| ASV665 | FALSE | TRUE | Only wet | *unknown* | Bacillaceae |
| ASV6674 | FALSE | TRUE | Only wet | *Prevotella* | Prevotellaceae |
| ASV685 | FALSE | TRUE | Only wet | *Pseudomonas* | Pseudomonadaceae |
| ASV689 | FALSE | TRUE | Only wet | *Lysinibacillus* | Planococcaceae |
| ASV6909 | FALSE | TRUE | Only wet | *Garicola* | Micrococcaceae |
| ASV692 | FALSE | TRUE | Only wet | *unknown* | Bacillaceae |
| ASV699 | FALSE | TRUE | Only wet | *unknown* | Oscillospiraceae |
| ASV70 | FALSE | TRUE | Only wet | *unknown* | Bacillaceae |
| ASV7092 | FALSE | TRUE | Only wet | *Candidatus Saccharimonas* | Saccharimonadaceae |
| ASV715 | FALSE | TRUE | Only wet | *unknown* | Alcaligenaceae |
| ASV7274 | FALSE | TRUE | Only wet | *unknown* | Bacillaceae |
| ASV7547 | FALSE | TRUE | Only wet | *unknown* | Longimicrobiaceae |
| ASV7599 | FALSE | TRUE | Only wet | *unknown* | UCG-010 |
| ASV769 | FALSE | TRUE | Only wet | *Saccharopolyspora* | Pseudonocardiaceae |
| ASV7832 | FALSE | TRUE | Only wet | *[Anaerorhabdus] furcosa group* | Erysipelotrichaceae |
| ASV788 | FALSE | TRUE | Only wet | *Salinicoccus* | Staphylococcaceae |
| ASV7977 | FALSE | TRUE | Only wet | *unknown* | Oscillospiraceae |
| ASV80 | FALSE | TRUE | Only wet | *Saccharomonospora* | Pseudonocardiaceae |
| ASV800 | FALSE | TRUE | Only wet | *unknown* | Bacillaceae |
| ASV802 | FALSE | TRUE | Only wet | *Gracilibacillus* | Bacillaceae |
| ASV803 | FALSE | TRUE | Only wet | *Ornithinimicrobium* | Intrasporangiaceae |
| ASV804 | FALSE | TRUE | Only wet | *unknown* | unknown |
| ASV806 | FALSE | TRUE | Only wet | *Aminobacter* | Rhizobiaceae |
| ASV8102 | FALSE | TRUE | Only wet | *Rikenellaceae RC9 gut group* | Rikenellaceae |
| ASV814 | FALSE | TRUE | Only wet | *Allobacillus* | Bacillaceae |
| ASV82 | FALSE | TRUE | Only wet | *Lysinibacillus* | Planococcaceae |
| ASV8264 | FALSE | TRUE | Only wet | *Methanobrevibacter* | Methanobacteriaceae |
| ASV829 | FALSE | TRUE | Only wet | *Oceanobacillus* | Bacillaceae |
| ASV8317 | FALSE | TRUE | Only wet | *Pusillimonas* | Alcaligenaceae |
| ASV837 | FALSE | TRUE | Only wet | *Actinomadura* | Thermomonosporaceae |
| ASV839 | FALSE | TRUE | Only wet | *Bacteroides* | Bacteroidaceae |
| ASV843 | FALSE | TRUE | Only wet | *unknown* | Rhizobiaceae |
| ASV847 | FALSE | TRUE | Only wet | *Truepera* | Trueperaceae |
| ASV850 | FALSE | TRUE | Only wet | *unknown* | Rhodobacteraceae |
| ASV860 | FALSE | TRUE | Only wet | *Bacillus* | Bacillaceae |
| ASV8614 | FALSE | TRUE | Only wet | *Erysipelothrix* | Erysipelotrichaceae |
| ASV8791 | FALSE | TRUE | Only wet | *unknown* | Bacillaceae |
| ASV8809 | FALSE | TRUE | Only wet | *Clostridium sensu stricto 1* | Clostridiaceae |
| ASV89 | FALSE | TRUE | Only wet | *Lysobacter* | Xanthomonadaceae |
| ASV890 | FALSE | TRUE | Only wet | *unknown* | UCG-010 |
| ASV8910 | FALSE | TRUE | Only wet | *unknown* | Bacillaceae |
| ASV896 | FALSE | TRUE | Only wet | *unknown* | Intrasporangiaceae |
| ASV9066 | FALSE | TRUE | Only wet | *Candidatus Saccharimonas* | Saccharimonadaceae |
| ASV9070 | FALSE | TRUE | Only wet | *unknown* | unknown |
| ASV9073 | FALSE | TRUE | Only wet | *dgA-11 gut group* | Rikenellaceae |
| ASV915 | FALSE | TRUE | Only wet | *unknown* | MWH-CFBk5 |
| ASV921 | FALSE | TRUE | Only wet | *Ornithinimicrobium* | Intrasporangiaceae |
| ASV924 | FALSE | TRUE | Only wet | *Melghirimyces* | Thermoactinomycetaceae |
| ASV930 | FALSE | TRUE | Only wet | *unknown* | Nitriliruptoraceae |
| ASV941 | FALSE | TRUE | Only wet | *unknown* | Sphingobacteriaceae |
| ASV9454 | FALSE | TRUE | Only wet | *Natronococcus* | Haloferacaceae |
| ASV947 | FALSE | TRUE | Only wet | *Pusillimonas* | Alcaligenaceae |
| ASV949 | FALSE | TRUE | Only wet | *unknown* | Paenibacillaceae |
| ASV95 | FALSE | TRUE | Only wet | *Salinimicrobium* | Flavobacteriaceae |
| ASV956 | FALSE | TRUE | Only wet | *Oceanobacillus* | Bacillaceae |
| ASV9606 | FALSE | TRUE | Only wet | *Ruminobacter* | Succinivibrionaceae |
| ASV9629 | FALSE | TRUE | Only wet | *unknown* | Flavobacteriaceae |
| ASV968 | FALSE | TRUE | Only wet | *unknown* | unknown |
| ASV977 | FALSE | TRUE | Only wet | *unknown* | Prevotellaceae |
| ASV986 | FALSE | TRUE | Only wet | *unknown* | Bacillaceae |
| ASV987 | FALSE | TRUE | Only wet | *unknown* | UCG-010 |
| ASV997 | FALSE | TRUE | Only wet | *unknown* | Bacillaceae |
| ASV9998 | FALSE | TRUE | Only wet | *unknown* | unknown |

*Supplementary Table 3: Functional annotation of significant genes (Figures 8–10)*

| Gene / Feature | Protein / Enzyme | Main Function | Stress / Environmental Relevance |
| --- | --- | --- | --- |
| **ytfJ** | Stress response protein YtfJ | Predicted membrane protein; induced under osmotic and heat stress in *Bacillus* spp. | General stress adaptation |
| **spmA**, **spmB** | Spore maturation proteins A & B | Small acid-soluble proteins stabilizing spore cortex; important in spore heat resistance | Heat and desiccation tolerance |
| **mecA** | ClpC–MecA adaptor protein | Regulatory adaptor for ClpC protease; mediates degradation of misfolded proteins | Protein quality control under heat or oxidative stress |
| **argB** | Acetylglutamate kinase | Arginine biosynthesis | Metabolic enzyme; can be induced under osmotic stress |
| **yhcF** | Stress response membrane protein YhcF | Putative chaperone-like protein involved in envelope stress | General stress response |
| **anti-sigma B factor antagonist** | RsbV (anti-σ^B antagonist) | Regulates activation of σ^B, the general stress sigma factor | Master regulator of general stress (including heat and osmotic) |
| **DUF2621** | Conserved hypothetical protein (domain of unknown function 2621) | Often co-located with oxidative stress operons | Putative stress-related role |
| **cccA** | Cation efflux system protein CccA | Ferric iron exporter | Iron homeostasis, protection against oxidative stress |
| **yqiQ** | Universal stress protein-like | Small cytoplasmic protein induced under multiple stresses | Osmotic/oxidative/heat stress protection |
| **lmbE** | N-acetylglucosaminidase | Cell-wall remodeling enzyme | Structural adaptation under growth or stress |
| **rpsL / rplE / rplL / rpIM** | Ribosomal proteins (S12, L5, L7/L12, L13) | Protein synthesis machinery | Expression shifts under heat and oxidative stress; translation regulation |
| **rpoC** | RNA polymerase β′ subunit | Transcription core enzyme | Upregulated during transcriptional stress control |
| **pdxS** | Pyridoxal 5′-phosphate synthase subunit | Vitamin B₆ biosynthesis | Cofactor in antioxidant defense reactions |
| **ftsZ** | Cell division protein FtsZ | Septum formation (Z-ring) | Sensitive to oxidative and temperature stress |
| **tspO** | Tryptophan-rich sensory protein | Membrane stress sensor linked to redox control | Stress-sensing and membrane potential regulation |
| **superoxide dismutase (sodA)** | Superoxide dismutase | Converts O₂⁻ radicals to H₂O₂ | Antioxidant enzyme, oxidative stress protection |
| **cosR** | Copper-sensing transcriptional repressor | Regulates copper resistance operons | Heavy-metal and oxidative stress response |
| **regX3** | Two-component system regulator | Controls phosphate assimilation and oxidative stress genes | Nutrient/oxidative stress control |
| **csoR** | Copper-sensing repressor (CsoR family) | Regulates copper efflux and detox genes | Oxidative/metal stress |
| **acoB**, **accA**, **fabA** | Acetoin dehydrogenase, Acetyl-CoA carboxylase, 3-hydroxydecanoyl-ACP dehydratase–isomerase | Carbon and lipid metabolism | Membrane remodeling under stress (*fab*A), carbon flow adjustment |
| **glyA** | Serine hydroxymethyltransferase | One-carbon metabolism | Redox balance; linked to energy stress |
| **glnA** | Glutamine synthetase | Ammonia assimilation | Nitrogen and osmotic balance under stress |
| **nirA** | Ferredoxin-nitrite reductase | Nitrate → ammonia reduction | Denitrification / nitrogen metabolism |
| **zupT** | Zinc transporter | Zn²⁺ import (ZIP family) | Metal homeostasis; oxidative stress resilience |
| **ppk** | Polyphosphate kinase | Polyphosphate synthesis | Energy storage; stress survival mechanism |
| **thiG** | Thiazole synthase | Thiamine (vitamin B₁) biosynthesis | Cofactor supply for oxidative stress enzymes |
| **csoR / istB** | Copper resistance and integrase ATPase | Metal detoxification / genome mobility | Oxidative and environmental stress |
| **rri1** | 16S rRNA processing factor | Ribosome biogenesis | Translation regulation under stress |
| **caiD** | Crotonobetaine hydratase | Carnitine metabolism | Compatible solute and osmoprotection |
| **autoproteolytic hydrolase / hydrolase / oxidoreductase** | Enzyme families | Protein turnover / redox balance | General stress-induced catalytic activity |
| **A0A1B2YM59 / BVRB_xxxxx** | Hypothetical or annotated proteins | Various (mostly oxidative or osmotic) | Potential novel stress-related functions |

References

Ahlmann-Eltze, C. and Patil, I. (2021) ggsignif: R Package for Displaying Significance Brackets for “ggplot2.” *PsyArxiv*.

Barrett T, Dowle M, Srinivasan A, Gorecki J, Chirico M, Hocking T, et al. (2025) data.table: Extension of “data.frame”. R package version 1.17.99.

Callahan, B.J., McMurdie, P.J., and Holmes, S.P. (2017) Exact sequence variants should replace operational taxonomic units in marker-gene data analysis. *The ISME Journal 2017 11:12* **11**: 2639–2643.

Callahan, B.J., McMurdie, P.J., Rosen, M.J., Han, A.W., Johnson, A.J.A., and Holmes, S.P. (2016) DADA2: High-resolution sample inference from Illumina amplicon data. *Nat Methods* **13**: 581–583.

Hadley Wickham (216AD) ggplot2: Elegant Graphics for Data Analysis. *J R Stat Soc Ser A Stat Soc* **174**: 245–246.

Liu, C., Cui, Y., Li, X., and Yao, M. (2021) microeco: an {R} package for data mining in microbial community ecology. *FEMS Microbiol Ecol* **97**: 255.

McMurdie, P.J. and Holmes, S. (2013) phyloseq: An R Package for Reproducible Interactive Analysis and Graphics of Microbiome Census Data. *PLoS One* **8**: e61217.

Philippou Katerina, Phokas Alexandros, Savvides Andreas M., Pissaridou Panayiota, Alexandrou Anna, Sparaggis Dionysios, et al. (2026) Impact of Simulated Summer Rainfall on GHG and Ammonia Emissions from Manure in Cyprus semi-arid conditions: A Comparative Study of Dairy Cows and Goats. *Environmental Management*. UNDER REVISIONS

Wickham, H., Averick, M., Bryan, J., Chang, W., McGowan, L., François, R., et al. (2019) Welcome to the Tidyverse. *J Open Source Softw* **4**: 1686.

Wickham H, Pedersen T, and Seidel D (2025) scales: Scale Functions for Visualization. R package version 1.4.0.
